# Presenilin‐1 mutation position influences amyloidosis, small vessel disease, and dementia with disease stage

**DOI:** 10.1002/alz.13729

**Published:** 2024-02-21

**Authors:** Nelly Joseph‐Mathurin, Rebecca L. Feldman, Ruijin Lu, Zahra Shirzadi, Carmen Toomer, Junie R. Saint Clair, Yinjiao Ma, Nicole S. McKay, Jeremy F. Strain, Collin Kilgore, Karl A. Friedrichsen, Charles D. Chen, Brian A. Gordon, Gengsheng Chen, Russ C. Hornbeck, Parinaz Massoumzadeh, Austin A. McCullough, Qing Wang, Yan Li, Guoqiao Wang, Sarah J. Keefe, Stephanie A. Schultz, Carlos Cruchaga, Gregory M. Preboske, Clifford R. Jack, Jorge J. Llibre‐Guerra, Ricardo F. Allegri, Beau M. Ances, Sarah B. Berman, William S. Brooks, David M. Cash, Gregory S. Day, Nick C. Fox, Michael Fulham, Bernardino Ghetti, Keith A. Johnson, Mathias Jucker, William E. Klunk, Christian la Fougère, Johannes Levin, Yoshiki Niimi, Hwamee Oh, Richard J. Perrin, Gerald Reischl, John M. Ringman, Andrew J. Saykin, Peter R. Schofield, Yi Su, Charlene Supnet‐Bell, Jonathan Vöglein, Igor Yakushev, Adam M. Brickman, John C. Morris, Eric McDade, Chengjie Xiong, Randall J. Bateman, Jasmeer P. Chhatwal, Tammie L. S. Benzinger, for the Dominantly Inherited Alzheimer Network

**Affiliations:** ^1^ Washington University School of Medicine in Saint Louis St. Louis Missouri USA; ^2^ Massachusetts General Hospital Brigham and Women's Hospital Harvard Medical School Boston Massachusetts USA; ^3^ Keck School of Medicine University of Southern California Los Angeles California USA; ^4^ Meharry School of Medicine Meharry College Nashville Tennessee USA; ^5^ Mayo Clinic Rochester Minnesota USA; ^6^ Institute for Neurological Research FLENI Montañeses Buenos Aires Argentina; ^7^ University of Pittsburgh Medical Center Pittsburgh Pennsylvania USA; ^8^ Neuroscience Research Australia Sydney NSW Australia; ^9^ University of New South Wales Sydney NSW Australia; ^10^ UK Dementia Research Institute and Dementia Research Centre, UCL Queen Square Institute of Neurology University College London London UK; ^11^ Mayo Clinic Jacksonville Florida USA; ^12^ Royal Prince Alfred Hospital Camperdown NSW Australia; ^13^ Indiana University School of Medicine Indianapolis Indiana USA; ^14^ German Center for Neurodegenerative Diseases (DZNE) Tübingen Germany; ^15^ Hertie Institute for Clinical Brain Research University of Tübingen Tübingen Germany; ^16^ University hospital Tübingen Tübingen Germany; ^17^ German Center for Neurodegenerative Diseases (DZNE) Munich Germany; ^18^ Ludwig Maximilian University of Munich Munich Germany; ^19^ Munich Cluster for Systems Neurology (SyNergy) Munich Germany; ^20^ The University of Tokyo Bunkyo City Tokyo Japan; ^21^ Brown University Providence Rhode Island USA; ^22^ Banner Alzheimer Institute Banner Health Phoenix Arizona USA; ^23^ Department of Nuclear Medicine Technical University of Munich Munich Germany; ^24^ Columbia University Medical Center New York New York USA

**Keywords:** autosomal dominant Alzheimer's disease (ADAD), cerebral amyloid angiopathy (CAA), codon 200, dominantly inherited Alzheimer's disease (DIAD), microbleeds, microhemorrhages, peak width of skeletonized mean diffusivity (PSMD), PiB‐PET, presenilin‐1, PSEN1, small vessel disease (SVD), white matter hyperintensity (WMH)

## Abstract

**INTRODUCTION:**

Amyloidosis, including cerebral amyloid angiopathy, and markers of small vessel disease (SVD) vary across dominantly inherited Alzheimer's disease (DIAD) presenilin‐1 (*PSEN1)* mutation carriers. We investigated how mutation position relative to codon 200 (pre‐/postcodon 200) influences these pathologic features and dementia at different stages.

**METHODS:**

Individuals from families with known *PSEN1* mutations (*n* = 393) underwent neuroimaging and clinical assessments. We cross‐sectionally evaluated regional Pittsburgh compound B‐positron emission tomography uptake, magnetic resonance imaging markers of SVD (diffusion tensor imaging‐based white matter injury, white matter hyperintensity volumes, and microhemorrhages), and cognition.

**RESULTS:**

Postcodon 200 carriers had lower amyloid burden in all regions but worse markers of SVD and worse Clinical Dementia Rating^®^ scores compared to precodon 200 carriers as a function of estimated years to symptom onset. Markers of SVD partially mediated the mutation position effects on clinical measures.

**DISCUSSION:**

We demonstrated the genotypic variability behind spatiotemporal amyloidosis, SVD, and clinical presentation in DIAD, which may inform patient prognosis and clinical trials.

**Highlights:**

- Mutation position influences Aβ burden, SVD, and dementia.
- *PSEN1* pre‐200 group had stronger associations between Aβ burden and disease stage.
- *PSEN1* post‐200 group had stronger associations between SVD markers and disease stage.
- *PSEN1* post‐200 group had worse dementia score than pre‐200 in late disease stage.
- Diffusion tensor imaging‐based SVD markers mediated mutation position effects on dementia in the late stage.

## 1 INTRODUCTION

Symptoms in dominantly inherited Alzheimer's disease (DIAD) typically present before age 65, with a mean onset around 45 years.^1^ This familial form is characterized by mutations in genes involved in amyloid beta (Aβ) metabolism: Aβ precursor protein (*APP*), presenilin‐1 (*PSEN1)*, and presenilin‐2 (*PSEN2*). Mutations in these genes alter the amount or nature of Aβ peptides leading to increasing accumulation and deposition of Aβ in the brain parenchyma or vessels. These findings are central to the Aβ hypothesis of AD, which posits that Aβ accumulation initiates the pathogenic cascade leading to neurodegeneration.^2^ DIAD shows patterns of disease progression similar to those seen in sporadic late onset AD (LOAD), with an initial accumulation of Aβ, followed by a decrease in cerebral metabolism, then brain atrophy^3^ and tau pathology.^4^ However, mutation‐dependent variability is observed in the biomarker and clinical presentations of DIAD. The age at symptom onset (AAO) varies within the DIAD population,^1^ and some pathogenic variants are associated with specific phenotypes such as marked small vessel disease (SVD) abnormalities,^5^ parkinsonism,^6^ or spastic paraparesis.^7^ Recent work from our group also described variable Aβ burden and accumulation as a function of mutation location in *APP*, *PSEN1*, and *PSEN2*.^8^

Pathogenic *PSEN1* gene mutations affect nearly 80% of known DIAD families.^9^ To date, about 308 of such mutations have been reported (www.alzforum.org/mutations/psen‐1), with strong evidence that mutation location within the gene influences AD pathogenesis. Neuropathology work from Mann and colleagues previously described two different amyloidosis phenotypes in *PSEN1* mutation carriers, with the mutations postcodon 200 likely to have severe cored plaques and cerebral amyloid angiopathy (CAA).^10^ Several studies, including from our group, have further investigated the association between disease presentation and genotype using pre‐/postcodon 200 grouping and reported differences in imaging phenotypes and clinical presentations.^8^, ^11^, ^12^, ^13^ For instance, imaging and neuropathology studies from Ryan et al. reported more severe white matter lesions in individuals with postcodon 200 *PSEN1* mutations compared to precodon 200, suggesting more severe SVD in the former.^11^, ^14^ Our group reported higher cortical and striatal Aβ burden and steeper accumulation in individuals with precodon 200 *PSEN1* mutations compared to postcodon 200.^8^

These previous studies clearly established that *PSEN1* mutation position influenced Aβ deposition and white matter lesions. However, it remains unclear whether mutation location relative to codon 200 influences how these imaging features present regionally and as a function of disease stage and whether mutation location could directly or indirectly influence clinical presentations. Moreover, other imaging markers of SVD including microhemorrhages have not yet been thoroughly studied. We used the large dataset of the Dominantly Inherited Alzheimer Network (DIAN) observational study to investigate, using a cross‐sectional approach, the influence of *PSEN1* mutation position with disease stage on (i) the topography of Aβ burden as measured in vivo by 11C‐Pittsburgh compound B (PiB) positron emission tomography (PET); (ii) the presence and topography of SVD as estimated with established neuroimaging metrics of CAA, white matter hyperintensity (WMH) volumes, and microhemorrhages^15^, ^16^, ^17^ and with novel diffusion imaging‐based metrics of white matter injury^18^, ^19^; and (iii) cognition and clinical presentation.

## 2 METHODS

### 2.1 Participants

The DIAN study enrolls individuals from families with a known DIAD mutation in the *APP*, *PSEN1*, or *PSEN2* gene. We used the 15th DIAN data freeze (January 2009–June 2020) and identified, out of 583 participants, 418 participants from families with *PSEN1* mutations who had undergone magnetic resonance imaging (MRI), PET, genetic, and clinical assessments. We evaluated 393 individuals whose imaging data passed quality‐controlled evaluations (see Figure S1 in Supplemental Material for data selection details). Among them, 148 were mutation non‐carriers (NCs) and 245 were mutation carriers (MCs) of a pathogenic *PSEN1* variant, including 83 with their family mutation precodon 200 (pre‐200 MC) and 162 with their family mutation postcodon 200 (post‐200 MC). Each DIAN site's Institutional Review Board approved all study procedures. All participants or their caregivers provided written informed consent approved by their local institution's review board. Standardized clinical and imaging assessments were obtained according to DIAN study protocols.

### 2.2 Clinical and cognitive assessments

The Clinical Dementia Rating^®^ (CDR^®^) defines the absence of dementia as CDR 0 (asymptomatic) and the presence of clinical impairment with a score greater than zero (CDR > 0).^20^ The CDR sum of boxes (CDR‐SB) score, based on the sum of CDR subscales, was used as a more continuous measure of disease stage.^21^, ^22^ Disease stage was also estimated using the estimated years to symptom onset (EYO) calculated at the individual level.^1^ EYO was defined as the participant's age at each assessment minus the estimated age of symptom onset for their specific family mutation when asymptomatic or, if symptomatic, minus their age at onset, determined by clinicians using comprehensive questionnaires and other clinical measures to trace back when the participant first became symptomatic. By design, fewer individuals entering the study are symptomatic, and their disease duration may be estimated with EYO. For each DIAN participant, the AAO is defined according to the specific variant mean age of onset or parental age of symptom onset (in case the specific variant mean age of onset is unknown).

**RESEARCH IN CONTEXT**

1. **Systematic review**: The literature was reviewed using PubMed and appropriately cited. *PSEN1* mutations account for the majority (∼80%) of dominantly inherited Alzheimer's disease (DIAD). Previous studies reported a distinct profile of pathologies influenced by *PSEN1* mutation position relative to codon 200.
2. **Interpretation**: We observed elevated Aβ burden starting in posterior cortical regions between Estimated Years to symptom Onset (EYO) −20 and −15 and elevated white matter lesions in posterior periventricular areas between EYO −10 and 0 in postcodon 200; in precodon 200, Aβ burden was widespread between EYO −10 and −5 years without pronounced white matter lesions. Whereas clinical presentation of both groups appeared similar, worse impairment, partially mediated by SVD, was observed in postcodon 200 in late disease stages.
3. **Future directions**: This study demonstrates the influence of mutation position on the progression of DIAD and provides important insights into the design of future clinical trials.

Global cognition was estimated with a composite score based on *z*‐score average of the Mini‐Mental State Examination (MMSE)^23^ and three other tests of episodic memory, complex attention, and processing speed.^24^ Other clinical, vital, and vascular‐related variables included systolic and diastolic blood pressure, mean arterial blood pressure (MAP), hypertension, hypercholesterolemia, diabetes, stroke history, and Hachinski ischemia score.^25^ Variables from clinician diagnosis and neurological examination were also reviewed for specific disease presentation in both carrier groups (eg, visuospatial, language, and motor deficits, comorbidities) following the criteria of the Uniform Data Set (UDS) protocols.^26^ Motor and behavioral deficits and age of onset related to clinical decline were based on clinical assessment and reports using UDS forms B9 and D1. The assessment of motor symptoms included reports of abnormal gait, tremor, fall, and/or slowness. Clinicians were blinded to the exact family mutation and to the non‐carrier/carrier status of the participants when proceeding with clinical assessments.

### 2.3 Genotyping and codon grouping

All DIAN participants underwent genetic analyses to assess DIAD family pedigrees, including details on the *PSEN1* mutation (eg, missense, insertion), and apolipoprotein E (*APOE)* genotype. *PSEN1* coding sequences were evaluated for missense, nonsense, and splice‐site sequence variants with standard procedures, as previously described.^27^ Mutations were classified as pre‐ or postcodon 200. A deletion of exon 9 was classified as a mutation postcodon 200 and a deletion of intron 4 as a mutation precodon 200. The *APOE* genotype was used to define *APOE* ɛ4 status as the presence (*APOE* ɛ4 carrier) or absence (*APOE* ɛ4 non‐carrier) of an *APOE* ɛ4 allele.

### 2.4 Imaging acquisition protocol

Participants underwent MRI and PET sessions using standardized procedures for all DIAN sites as previously described.^28^, ^29^ Accelerated magnetization‐prepared rapid acquisition with gradient echo (MPRAGE) was acquired with repetition time (TR)/ echo time (TE) = 2300/52.95 ms and resolution = 1.0 × 1.0 × 1.2 mm^3^. T2‐weighted fluid‐attenuated inversion recovery (FLAIR) was acquired at TR/TE = 9000/90 ms and resolution = 0.86 × 0.86 × 5.0 mm^3^. Two‐dimensional EPI diffusion tensor imaging (DTI) was performed at TR/TE = 8100/87 ms, resolution = 2.5 × 2.5 × 2.5 mm^3^, number of directions = 64, b‐values = 0, and 1000 s/mm^2^. A T2*‐weighted or susceptibility‐weighted imaging (SWI) sequence was acquired with TR/TE = 650/20 ms, and resolution = 0.8 × 0.8 × 4 mm^3^ or TR/TE = 28/20 ms, and resolution = 0.7 × 0.7 × 2mm^3^, respectively. ^11^C‐PiB PET was acquired with a 70‐min scan starting at injection or a 30‐min scan beginning 40 min following the injection of an ∼13‐mCi bolus of ^11^C‐PiB.

### 2.5 Image processing

Volumetric segmentation and cortical reconstruction were performed using FreeSurfer version 5.3‐HCP (https://surfer.nmr.mgh.harvard.edu/) and PET scans were processed using the PET Unified Pipeline (PUP).^30^, ^31^ The data were partial volume corrected using a regional spread function technique and standardized uptake value ratios (SUVRs) were calculated from the 40‐ to 70‐min time window after tracer injection using the cerebellar cortex as a reference region.^32^ SUVRs from the lateral orbitofrontal, medial orbitofrontal, middle temporal, precuneus, rostral middle frontal, superior frontal, and superior temporal cortices from both the left and right hemispheres were averaged to define the mean cortical Aβ SUVR with a threshold of 1.42 used to define PiB‐PET positivity.^30^ Regional Aβ SUVR evaluations included 34 cortical and six subcortical FreeSurfer regions of interest (ROIs).

The presence and count of microhemorrhages in the entire brain, as well as in frontal, parietal, temporal, and occipital areas, were reviewed and reported by author CRJ, a trained radiologist, from T2* or SWI sequences, using a previously described approach.^28^, ^33^ Note that the sequence type did not influence results in previous DIAN studies,^28^ and the proportion of SWI/T2* was similar among groups in the current study (Table 1). Total WMH volumes were extracted from FLAIR images using the lesion growth algorithm of the segmentation toolbox in Statistical Parametric Mapping (SPM) version 8^34^ for all participants. A subset of participants studied in a previous data freeze (Figure S1 and Table S1) had additional subregional WMH volumes and/or global white matter injury metrics from DTI, peak width of skeletonized mean diffusivity (PSMD), as a novel imaging marker for SVD previously validated in ADNI.^18^ Subregional WMH volumes included total periventricular (PV) WMH, anterior PV, posterior PV, and deep (DWMH) and were calculated with a semi‐automatic segmentation approach^35^, ^36^ involving manual segmentation and quality control assessment performed by authors JFS and CK. Subregional WMH volumes were registered to the common Montreal Neurological Institute template. PSMD metrics and maps were generated using a publicly available script (http://www.psmd‐marker.com/), as previously described.^19^, ^37^ In brief, the DTI scans were preprocessed for eddy current and motion correction, then tensor‐fitted for data skeletonization and histogram analysis using FSL version 6.0.1 tools (eg, Tract‐Based Spatial Statistics and fractional anisotropy template). All PSMD maps were quality controlled. Of 337 processed images, 190 generated PSMD maps that passed quality control, and the metrics were subsequently included in the analyses.

**TABLE 1:** Baseline characteristics of participants.

Demographics	NC	Pre‐200 MC	Post‐200 MC	*p* value
*N* (%)	148 (37.7)	83 (21.1)	162 (41.2)	−
Age, mean (SD), years	35.8 (10.6)	35.5 (9.3)	38.7 (11.6)^a^	0.02
Female, *n* (%)	83 (56.1)	47 (56.6)	94 (58.0)	0.94
Education, mean (SD), years	15.1 (2.7)	14.8 (3.2)	14.2 (2.9)^a^	0.04
*APOE ɛ4* carriers, *n* (%)	43 (29.1)	16 (19.3)	54 (33.3)	0.07
**Familial AAO, mean (SD), years**	47.2 (7.3)	**44.0 (9.0)** ^b^, ^c^	**46.8 (7.2)**	0.005
EYO, mean (SD), years	−11.01 (11.6)	−8.0 (11.0)	−7.6 (11.0)^a^	0.02
EYO > 0, *n* (%)	23 (15.5)	29 (34.9)^b^	53 (32.7)^b^	<0.001
Asymptomatic, *n* (% of EYO > 0)	–	2 (6.9)	0 (0)	–
**CDR > 0, *n* (%)** ^h^	7 (4.7)	**28 (33.7)** ^d^	**62 (38.3)**	<0.001
CDR‐SB, mean (SD)^h^	0.04 (0.18)	1.10 (2.02)^a^	1.67 (3.53)^e^	<0.001
MMSE, mean (SD)^h^	29.0 (1.2)	27.0 (4.3)^b^	26.4 (5.8)^e^	<0.001
Cognitive composite, mean (SD)^h^	0.06 (0.47)	−0.38 (1.03)^b^	−0.50 (0.92)^e^	<0.001
**PiB‐PET positive, *n* (%)** ^h^	0 (0)	**36 (43.4)** ^d^	**41 (25.3)**	0.009
**Mean cortical PiB SUVR, mean (SD)** ^h^	1.0 (0.1)	**2.2 (1.3)** ^e^, ^f^	**1.8 (0.9)** ^e^	<0.001
SWI/sequence type for reads, *n* (%)	64 (43.2)	44 (55.7)	71 (43.8)	0.15
Stroke history, *n* (%)^g^	1 (0.6)	0 (0)	3 (1.9)	0.71
Systolic blood pressure, mean (SD), mmHg^g^	122.6 (16.7)	121.8 (14.2)	123.2 (13.9)	0.92
Diastolic blood pressure, mean (SD), mmHg^g^	76.1 (10.3)	73.7 (10.7)	76.3 (9.8)	0.18
Mean arterial blood pressure, mean (SD), mmHg^g^	91.6 (11.5)	89.7 (11.0)	91.9 (9.9)	0.39
Hachinski ischemia score, mean (SD)^g^	0.18 (0.45)	0.18 (0.65)	0.45 (1.22)	0.05
Hypertension history, *n* (%)^g^	23 (19.4)	3 (2.6)^a^	19 (12.7)	0.02
Hypercholesterolemia, *n* (%)^g^	16 (10.8)	5 (6.0)	25 (15.4)	0.34
Diabetes, *n* (%)^g^	5 (3.4)	2 (2.4)	2 (1.2)	0.28
Seizures, *n* (%)^g^	2 (1.4)	4 (4.8)	6 (3.7)	0.32
Abnormal gait, *n* (%)^g^	5 (3.4)	4 (4.8)	17 (10.5)	0.15
Tremor, *n* (%)^g^	7 (4.7)	5 (6.0)	9 (5.6)	0.88

*Note*: For the NC group, EYO is based on the age of the individual at visit and age at onset of their MC parent and AAO is based on the reported mean onset for the familial mutations shared by their families.

Variables and data in bold indicate significant differences between pre‐200 and post‐200 MC on post‐hoc tests.

Abbreviations: NC, non‐carriers; MC, mutation carrier; APOE ɛ4, apolipoprotein‐E allele ɛ4; EYO, estimated years to symptom onset; AAO, age at onset; CDR‐SB, Clinical Dementia Rating Sum of Boxes; MMSE, Mini‐Mental State Examination; PiB, Pittsburgh compound B; PET, positron emission tomography; SUVR, standardized uptake value ratio.

^a^ < .05: Significantly different from NC.

^b^ < .005: Significantly different from NC.

^c^ < .05: Pre‐200 significantly different from post‐200 MC (bold).

^d^ < .005: Pre‐200 significantly different from post‐200 MC (bold).

^e^ < .0005: Significantly different from NC.

^f^ < .0005: Pre‐200 significantly different from post‐200 MC (bold).

^g^ Comparison adjusted for age.

^h^ Comparison adjusted for age and age at onset.

### 2.6 Statistical analyses

All statistical approaches in the study employed R version 3.6.2 (www.R‐project.org/) or SAS 9.4 (SAS Institute Inc., Cary, NC). The model accuracy at each step of the analyses was evaluated to ensure no violations of underlying assumptions for the methods used. For the main analyses, PiB SUVRs, PSMD, PVWMH, anterior PV, and posterior PV variables were normally distributed, and total WMH volumes were log‐transformed to approximate a normal distribution before running the models. Deep WMH and microhemorrhage count followed a bimodal zero‐inflated distribution and were analyzed with two‐part zero‐inflated negative binomial mixed models (see details in Supplemental Material). For PiB SUVR analyses, tests for multiple regions or multiple time intervals were adjusted with the Benjamini‐Hochberg method to control for false discovery rate (FDR), and all thresholds for significance were defined at an adjusted *p* < 0.05 for the PiB SUVR analyses. Owing to the small numbers of regions evaluated in regional WMH or microhemorrhage analyses (*n* = 4 and 5, respectively) and the exploratory nature of our research question, no corrections for multiple regions were applied in the main report (unadjusted and adjusted *p* values are detailed in Supplemental Material). Missing values were considered missing at random. Sensitivity analyses were performed to assess the concordance of models with or without age or AAO as covariates (see details in Supplementary Material).

#### 2.6.1 Comparisons of evaluated groups

Demographics of the cross‐sectional cohort were compared among NCs, pre‐200 MCs, and post‐200 MCs using analysis of variance and Tukey honestly significant difference (HSD) post hoc tests for continuous variables and chi‐squared (χ^2^) tests for categorical variables. To account for the family AAO, ANCOVA/Tukey HSD, and logistic regression/Wald χ^2^ tests with age at onset as a covariate were performed to compare clinical, cognition, and neuroimaging measures. Due to the small sample size, clinical presentations, summarized from clinician diagnosis and neurological examination, were compared between carrier groups using *t* test, and χ^2^ tests or Fisher's exact tests, as appropriate.

#### 2.6.2 Models to assess mutation effect on PiB and SVD markers with EYO

Linear mixed effect (LME) models evaluated the effect of *PSEN1* mutation position on cross‐sectional relationships between regional PiB uptake and EYO for each ROI. Familial AAO, *APOE* ɛ4 status, sex, and education were accounted for in the model, and family cluster was included as a random effect. LME or negative binomial mixed effect models were used where appropriate based on the variable distribution (normal or bimodal zero‐inflated) to evaluate the effect of *PSEN1* mutation position on volumes of white matter lesions and the white matter injury metric and included *APOE* ɛ4 status, sex, education, MAP, and age instead of AAO as covariates to account for age‐related vascular factors, with family cluster as a random effect.

#### 2.6.3 Approach to assessing divergence of groups in PiB, SVD, and clinical measures

To investigate when each MC group became abnormal compared to NCs in regional PiB uptake, regional WMH volumes, PSMD, and clinical and cognitive measures, we compared mean variables estimated from the corresponding models per EYO categories. To best capture changes in disease course, EYO categories used a 5‐year range if no subgroup size was smaller than 5; otherwise a 10‐year range was used (Supplementary Material). For regional microhemorrhage count, due to the small number of individuals with non‐zero data, models utilized continuous EYO and reported estimates at EYO = −15, −10, −5, and 0.5 years. Covariates and random variables were the same for models with EYO as a categorical or continuous variable. For the clinical variables we used AAO, *APOE* ɛ4 status, sex, and education as covariates, and family cluster as a random effect (Supplemental Material).

#### 2.6.4 Mediation analysis

Lastly, we investigated whether the mutation position with disease stage influenced dementia as measured by CDR‐SB through the indirect effect of regional PiB uptake or SVD as a mediator. Mediation analyses were based on LME and were performed independently in carriers using the complete set of non‐missing data for each PiB region (*n* = 40, corrected for multiple comparisons) and for each metric of SVD significantly influenced by the mutation position as the mediator. In the latter analysis, SVD variables were log‐transformed. EYO was treated as continuous and effects conditional on EYO were evaluated at EYO = −15, −10, −5, 0, 0.5, and 1 years. Three close time points at EYO ≥ 0 were used to capture effects in late stages, while conditional effects were not evaluated beyond EYO = 1 due to small sample size. We used the mediation R package^38^ for the analyses utilizing a Bayesian approach. A sampled Markov chain Monte Carlo (*n* = 1000) was used to estimate posterior distribution and 95% confidence interval (CI) of the models (Supplemental Material).

## 3 RESULTS

### 3.1 Participant characteristics

The three groups (NC, pre‐200 MC, and post‐200 MC) were similar in male/female and *APOE* ɛ4 carrier proportions but different in age, EYO, and family AAO (Table 1). Among carriers, the *PSEN1* pre‐200 group had a younger mean AAO compared to the *PSEN1* post‐200 group (AAO of 44.0 ± 9.0 years vs 46.8 ± 7.2 years, *p <* 0.05). The pre‐200 group had higher proportions of PiB+ but lower proportions of symptomatic participants compared to post‐200 (Table 1). The symptomatic individuals had an estimated disease duration of EYO 3.2 ± 2.6 years in the pre‐200 MC years and 2.4 ± 6.1 years in post‐200 MC (*p =* 0.83). The MC groups were, however, similar in CDR‐SB, MMSE, and cognitive composite measures. Regarding vascular‐related variables, the three groups differed in prevalence of hypertension history, with the pre‐200 MC group showing the lowest percentage (19% for NC, 3% for pre‐200 MC, and 13% for post‐200 MC, *p =* 0.02, Table 1), and tended to differ in Hachinski ischemic score, with the post‐200 MC group showing the highest mean score (0.18 ± 0.45 for NC, 0.18 ± 0.65 for pre‐200 MC, and 0.45 ± 1.22 for post‐200 MC, *p =* 0.05, Table 1). The potentially confounding variables such as age, AAO, and vascular risk factors were accounted for in the models investigating mutation position effects on regional amyloidosis and SVD cross‐sectional associations with EYO.

### 3.2 Regional PiB uptake and spatiotemporal pattern across disease stages

**FIGURE 1:**
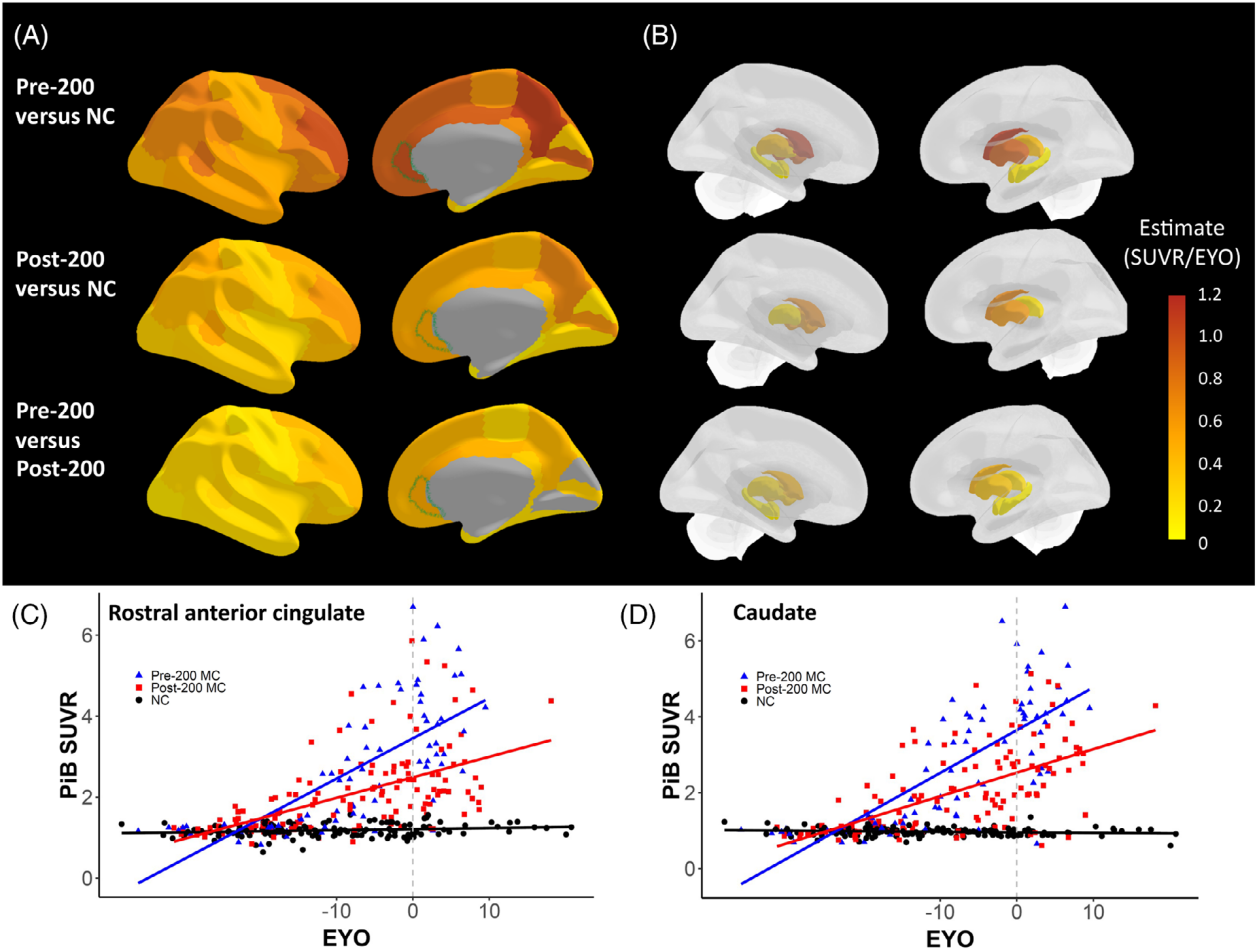
Cross‐sectional regional PiB uptake as a function of EYO. (A and B) Brain maps displaying significant different slope estimates between PiB SUVR and EYO for 34 cortical (A) and subcortical (B) FreeSurfer regions in pre‐200 carriers versus non‐carriers (top row), post‐200 carriers versus non‐carriers (middle row), and pre‐200 versus post‐200 (bottom row). The rostral anterior cingulate (green outline) and caudate showed the strongest divergence between pre‐ and post‐200. (C and D) Plots of cross‐sectional PiB uptake as a function of EYO for pre‐200 carriers (blue triangle), post‐200 carriers (red square), and non‐carriers (black circle) in rostral anterior cingulate (C) and in caudate (D). Color bar = estimates from LME model of PiB SUVR per EYO. EYO, estimated years to symptom onset; LME, linear mixed effect; NC, non‐carriers; MC, mutation carrier; PiB, Pittsburgh compound B; SUVR, standardized uptake value ratio.

Compared to the NC group, pre‐ and post‐200 MC shared eight out of their top 10 regions with the strongest associations of PiB uptake with EYO: precuneus, rostral middle frontal, rostral anterior frontal, frontal pole, medial orbitofrontal, and pericalcarine cortical regions and the caudate and putamen for subcortical regions, suggesting similar regional patterns of progression of Aβ burden (Figure 1, Table S2). Moreover, areas with the least effect were in the hippocampus and entorhinal cortex in the pre‐200 group, and these regions were the only ones not changing significantly with EYO in the post‐200 carriers, compared to NC. Pre‐200 MC showed greater levels of PiB uptake as a function of EYO compared to post‐200 for all regions, except for the cuneus and lingual cortex, suggesting different rates of Aβ accumulation in most cortical and subcortical areas for the same disease duration time (Table S2). The cortical and subcortical regions with the strongest effects were the rostral anterior cingulate and caudate, respectively (Table S1 and Figure 1).

**FIGURE 2:**
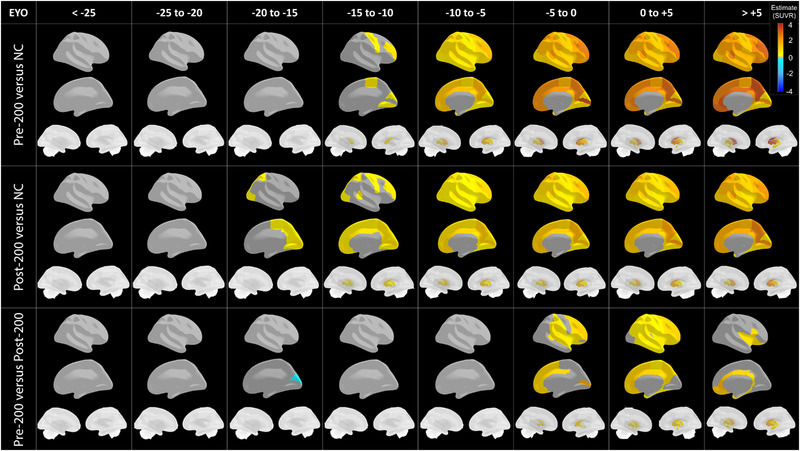
Topographical and temporal progression profiles of regional Aβ burden accumulation in pre‐ and post‐200 MC groups per 5‐year range of EYO. Brain maps displaying regional PiB uptake estimates for pre‐ and post‐200 MC groups compared to non‐carriers and pre‐ compared to post‐200 group (third row), split by EYO ranges of 5 years. Colored regions are significantly different in corresponding 5‐year range, and regions in gray are not significantly different after multiple comparisons. The post‐200 MC group starts to show abnormal accumulation in the occipital cortex between −20 and −15 EYO, while pre‐200 shows abnormal burden in occipital and frontal areas around −15 to −10 EYO but display higher values past −5 EYO and beyond. Color bar = estimates of regional mean PiB SUVR. EYO, estimated years to symptom onset; NC, non‐carriers; PiB, Pittsburgh compound B; SUVR, standardized uptake value ratio.

To investigate differences in the spatiotemporal pattern of Aβ cross‐sectional accumulation, we compared regional uptake based on EYO intervals of 5 years (Figure 2, Table S3a–c). Compared to NC, pre‐200 carriers started to show significant Aβ burden in the caudate and six cortical regions, including occipital (lingual and cuneus) and mostly frontal (paracentral, precentral, rostral middle frontal, and frontal pole) between EYO −15 and −10 and expanded to all regions throughout the brain between EYO −10 and −5 (Table S3a). Post‐200 carriers showed significant Aβ burden in seven cortical regions, including all four occipital (lateral occipital, lingual, cuneus, and pericalcarine) and parieto‐frontal (precuneus, superior parietal, and paracentral) regions between EYO −20 and −15 compared to NCs, which expanded to all medial frontal areas between EYO −15 and −10, then throughout the brain by EYO −10 −5 (Table S3b). Compared to each other, post‐200 showed higher uptake in the cuneus between EYO −20 and −15, then no difference in regional, until between EYO −5 and +5, where pre‐200 had higher Aβ burden in an increasing number of regions (19 then 35), particularly in frontal and temporal areas. For EYO > 5, this difference was seen in fewer regions, as the pre‐200 plateau and post‐200 reached similar levels (Figure 2 and Table S3c).

### 3.3 Pattern of progression of markers of SVD

#### 3.3.1 PSMD metric

**FIGURE 3:**
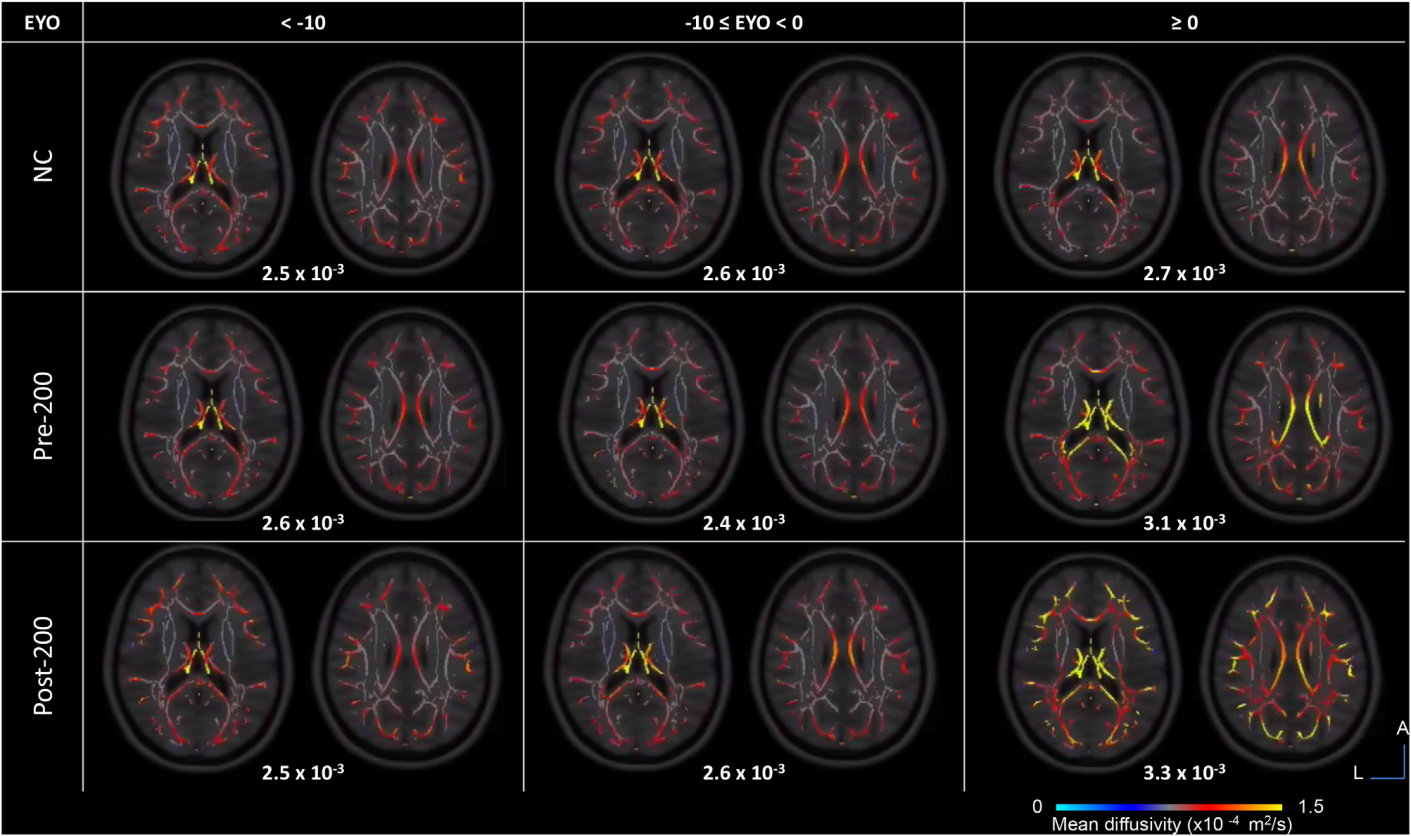
PSMD maps in NCs and pre‐ and post‐200 carriers per 10‐year range of EYO. Axial sections representing averaged mean diffusivity maps for each group per ranges of 10 years. High mean diffusivity in yellow reflects more injury in white matter. Late stages EYO ≥ 0 show higher mean diffusivity in carrier groups. The corresponding average PSMD values per group are indicated in reference (units in square millimeters per second [mm^2^/s]). EYO, estimated years to symptom onset; NC, non‐carrier; PSMD, peak width of skeletonized mean diffusivity; SUVR, standardized uptake value ratio. Color bar = mean diffusivity. Image orientations: L, left; A, anterior.

**FIGURE 4:**
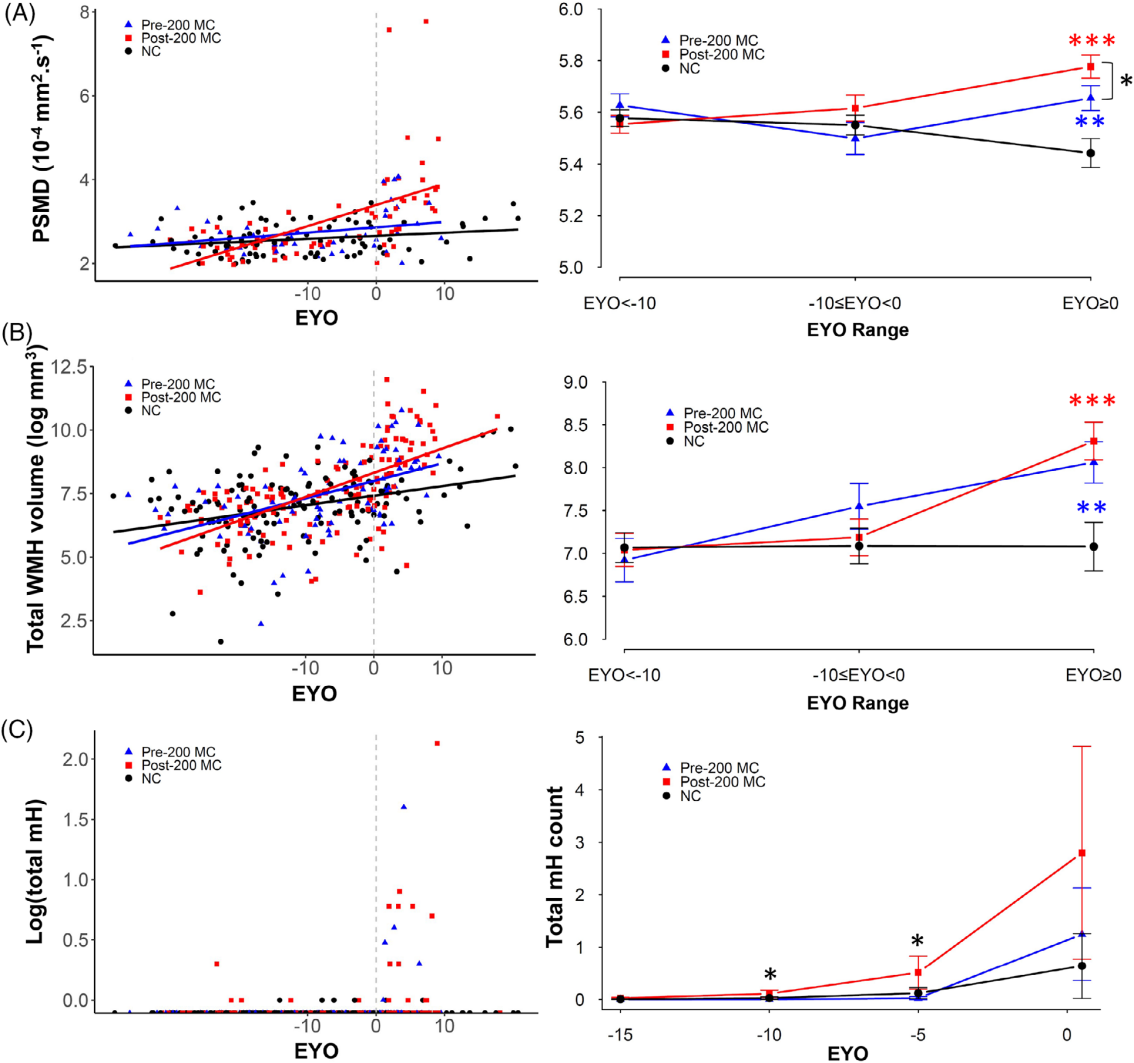
Cross‐sectional progression of global imaging markers of SVD in NC and pre‐ and post‐200 MC groups. (A) Scatterplot of raw PSMD values as a function of EYO (left panel) and plots of LME mean estimates of PSMD for pre‐200 carriers (blue triangle), post‐200 carriers (red square), and non‐carriers (black circle) per 10‐year range of EYO (right panel). Post‐200 carriers have significantly higher injury with advanced EYO compared to NCs and pre‐200 MC. (B) Scatterplot of log‐transformed total WMH volume values as a function of EYO (left panel) and plots of LME mean estimates of log‐transformed total WMH volume per 10‐year range of EYO (right panel). Both pre‐ and post‐200 MC show larger volumes compared to NC at EYO≥0. (C) Scatterplot of log‐transformed total mH count (dark red horizontal line denotes 5 mH) as a function of EYO (left panel), and plots of negative binomial mixed effect models mean estimates of total count of mHs broken down by EYO −15, −10, −5, and 0. Post‐200 MC have more mHs compared to pre‐200 MC at EYO = −10 and EYO = −5 years. EYO, estimated years to symptom onset; LME, linear mixed effect; mH, microhemorrhage; MC, mutation carrier; NC, non‐carrier; PSMD, peak width of skeletonized mean diffusivity; PVWMH, periventricular white matter hyperintensity; SVD, small vessel disease; WMH, white matter hyperintensity. Significance levels: *p < 0.05; **p  < 0.005, ***p < 0.0005 in blue for pre‐200 versus NC, in red for post‐200 versus NC, and in black for pre‐200 versus post‐200 carriers.

PSMD was significantly higher with higher EYO for post‐200 carriers compared to NCs (estimates = 0.01 ± 0.002 10^−4^ mm^2^.s^−1^/year, *p* < 0.0001) but not for pre‐200 carriers compared to NCs (estimates = 0.003 ± 0.003 10^−4^ mm^2^.s^−1^/year, *p* = 0.28). The post‐200 carrier group had more white matter injury with higher EYO compared to pre‐200 carrier group (estimates = 0.01 ± 0.003 10^−4^ mm^2^.s^−1^/year, *p* < 0.0005; Table S4). Figure 3 illustrates average PSMD maps per EYO range of 10 years, showing high values in later stages for both carrier groups. Analyses by EYO category revealed that the differences between pre‐ and post‐200 mainly occurred with EYO≥0 (estimated mean difference = 0.12 ± 0.06 10^−4^ mm^2^.s^−1^/year, *p* < 0.05; Figure 4A and Table S5a).

#### 3.3.2 Total and regional WMH volumes

Compared to NCs, post‐200 MC showed larger total and regional volumes as a function of EYO, except for anterior PV and deep areas (eg, total WMH: estimates = 0.05 ± 0.01 mm^3^/year, *p* < 0.0001; PVWMH: estimates = 61.5 ± 27.4 mm^3^/year, *p* < 0.05; Table S5a, b in supporting information), while pre‐200 MC had significantly larger WMH volumes with EYO only for the total volume of WMH (total WMH: estimates = 0.03 ± 0.01 mm^3^/year, *p* < 0.05; PVWMH: estimates = −3.9 ± 30.0 mm^3^/year, *p* = 0.9; Table S5a, b in supporting information). While these associations were not significantly different between pre‐ and post‐200 MC for total WMH volumes (estimates = 0.02 ± 0.02 mm^3^/year, *p* = 0.27), they were for PVWMH volumes (estimates = 65.3 ± 31.8 mm^3^/year, *p* < 0.05) and tended to be for posterior PV (estimates = 39.4 ± 20.1 mm^3^/year, *p* = 0.05) with post‐200 having stronger associations. These differences were not observed in anterior PV or DWMH areas (Tables S4 and S6a, respectively).

**FIGURE 5:**
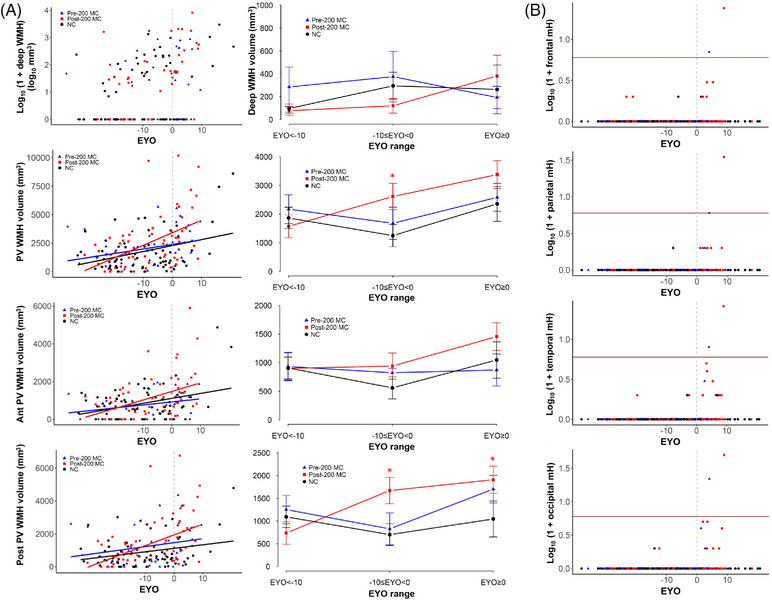
Cross‐sectional progression of regional imaging markers of SVD in non‐carrier, pre‐ and post‐200 MC groups. (A) Scatterplot of regional WMH volumes as a function of EYO (left panel) and plots of LME mean estimates (middle panel) of deep, total periventricular, anterior, and posterior periventricular (top to bottom) WMH for pre‐200 carriers (blue triangle), post‐200 carriers (red square), and NCs (black circle) by per 10‐year range of EYO. Deep WMH is presented as log (1 + *x*) to facilitate visualization. (B) Scatterplot of regional mH count as a function of EYO (right panel) for frontal, parietal, temporal, and occipital (top to bottom). Counts are presented as log (1 + *x*) to facilitate visualization and dark red horizontal lines denote five mHs. EYO, estimated years to symptom onset; mH, microhemorrhage; NC, non‐carrier; MC, mutation carrier; PVWMH, periventricular white matter hyperintensity; SVD, small vessel disease; WMH, white matter hyperintensity. Significance levels: *p < 0.05, **p < 0.005, ***p < 0.0005 in blue for pre‐200 versus NC, in red for post‐200 versus NC, and in black for pre‐200 versus post‐200 carriers.

Analysis by EYO category of a 10‐year range showed that both pre‐ and post‐200 had larger total WMH volumes compared to NCs only at EYO ≥ 0 (pre‐200 MC vs NC, estimates = 1.0 ± 0.3 log10 mm^3^, *p* < 0.005 and post‐200 MC vs NC, estimates = 1.2 ± 0.3 log 10 mm^3^, *p* < 0.0001; Figure 4B and Table S5a). When evaluating WMH subregions, compared to NCs, the post‐200 MC group showed significantly larger total and posterior PV WMH volumes at EYO −10 to 0 (estimated mean difference = 1366 ± 570 mm^3^, *p* value < 0.05 and estimated mean difference = 970 ± 361 mm^3^, *p* value < 0.05, for PVWMH and posterior PV, respectively), but not larger anterior PV nor deep WMH volumes (Figure 5, and Table S5b in supporting information). Pre‐200 MC did not have larger white matter lesions as measured by WMH volumes in any regions at any disease stage compared to NC (Table S5b and S6b).

#### 3.3.3 Total and regional microhemorrhages

Prevalence and counts of microhemorrhages are summarized in Table S7. The mutation position did not significantly influence the odds of having microhemorrhages (as opposed to not have any) in any brain regions but influenced their total count overall (Figures 4 and 5 and Table S8 in supporting information). We observed an effect of the mutation position on the total number of microhemorrhages in the brain as a function of EYO, with post‐200 carriers showing higher numbers of microhemorrhages at EYO −10 and EYO −5 compared to pre‐200 carriers (estimated mean difference = 0.006 ± 0.009, *p* value < 0.05 and 0.047 ± 0.043, *p* value < 0.05, respectively, Figure 4C and Table S8). The models run without controlling for age were consistent with the presented results.

### 3.4 Position mutation and clinical presentations

#### 3.4.1 Onset and prevalence in clinical presentations

Table 2 summarizes the clinical diagnoses, neurological exam findings, CDR domains and cognitive decline presentation among groups. The onset of clinical symptoms occurred at a younger age in the pre‐200 MC group compared to the post‐200 MCs, regardless of whether the symptoms were cognitive, motor, or behavioral. No significant difference was found in the prevalence of clinical presentation between pre‐ and post‐200 carrier groups. However, the prevalence of motor symptoms reported as part of the clinical decline in the post‐200 group tended to be twice as high as that in the pre‐200 group (15% vs. 7%, *p* value = 0.10, Table 2).

**TABLE 2:** Clinical presentation and characteristics of participants.

	NC	Pre‐200 MC	Post‐200 MC	*p* value^a^
	148	83	162	
Case with clinical decline estimated by clinician, *n* (%)	9 (6.1)	30 (36.1)	62 (38.3)	0.78
Age estimated at clinical decline, mean ± SD, years	33.2 ± 11.1	38.0 ± 7.5	44.3 ± 9.0	<0.005
Case with reported motor symptoms, *n* (%)	3 (2.0)	6 (7.2)	25 (15.4)	0.10
Age of onset of motor symptoms, mean ± SD, years	45 ± 12.5	40 ± 6.1	48.5 ± 8.5	0.02
Case with reported behavioral symptoms, *n* (%)	8 (5.4)	15 (18.1)	45 (27.8)	0.12
Age of onset of behavioral symptoms, mean ± SD, years	32.8 ± 14.4	36.78 ± 7.7	44.0 ± 11.4	0.01
CDR spatial orientation domain > 0, *n* (%)	0	18 (21.69)	40 (24.69)	0.64
CDR language domain > 0, *n* (%)	1 (0.68)	10 (12.05)	18 (11.11)	0.83
CDR behavior, comportment, and personality > 0, *n* (%)	5 (3.4)	10 (12.1)	32 (19.8)	0.15
Comorbidity clinician diagnosis				
Dementia with Lewy bodies, *n* (%)	0 (0)	0 (0)	1 (0.62)	1
Vascular dementia, *n* (%)	1 (0.68)	0 (0)	0 (0)	0.59
Corticobasal degeneration, *n* (%)	0 (0.0)	1 (1.20)	1 (0.62)	0.69
Depression, *n* (%)	26 (17.6)	19 (22.9)	34 (21.0)	0.75
Parkinson's disease, *n* (%)	0 (0)	0 (0)	1 (0.62)	1
Stroke, *n* (%)	1(0.68)	0(0)	2 (1.23)	0.80
Primary domain responsible of clinical decline (in cases with cognitive decline)				
Motor, *n* (% of cases with reported clinical decline)	2 (22.2)	0 (0)	3 (4.8)	0.55
Behavior, *n* (% of cases with reported clinical decline)	2 (22.2)	7 (23.3)	8 (12.9)	0.23
Cognition, *n* (% of cases with reported clinical decline)	4 (44.4)	22 (73.3)	50 (80.7)	0.58
Symptom of decline in cognition (in cases with clinical decline)				
Attention/concentration, *n* (%)	2 (22.2)	19 (63.3)	34 (54.8)	0.50
First recognized, *n* (% of cases with attention/concentration deficit)	1 (50)	3 (15.8)	3 (8.8)	0.65
Judgment and problem solving, *n* (%)	1 (11.1)	16 (53.3)	37 (59.7)	0.65
First recognized, *n* (% of cases with judgment and problem solving)	0 (0)	2 (12.5)	3 (8.1)	0.63
Language deficit, *n* (%)	1 (11.1)	9 (30)	21 (34.4) *(1)*	0.81
First recognized, *n* (% of cases with language deficit)	1 (100)	0 (0)	0 (0)	–
Memory, *n* (%)	4 (44.4)	28 (93.3)	57 (91.9)	1
First recognized, *n* (% of cases with memory symptoms)	4 (100)	23 (82.1)	50 (87.7)	0.52
Visuospatial function, *n* (%)	0 (0)	7 (24.1)	23 (37.1)	0.24
First recognized, *n* (% of cases with visuospatial function deficit)	0 (0)	0 (0)	1 (4.3)	1
Fluctuating cognition, *n* (%)	0 (0)	4 (13.8)	6 (9.7)	0.34

The data of the non‐carrier (NC) group are presented for reference and not included in the analyses as the research question aims to assess the potential effect of the mutation position on clinical presentation when symptoms are present. Variables reported in this table are based on clinician reports using UDS forms B9 and D1.

^a^ Statistical results of comparison between the two carrier groups.

#### 3.4.2 Measures of dementia and cognitive impairment

**FIGURE 6:**
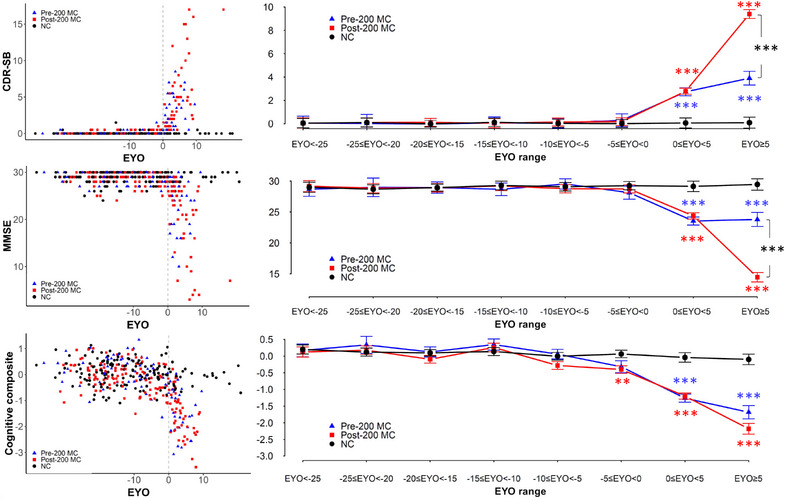
Cross‐sectional progression of clinical and cognitive measures in pre‐ and post‐200 MC groups per 5‐year range of EYO. Scatterplot of raw values as a function of EYO (left panel) and plots of LME mean estimates (right panel) of CDR‐SB (top), MMSE (middle), and cognitive composite (bottom) for pre‐200 (blue triangle), post‐200 carriers (red square), and NCs (black circle) by EYO ranges. Post‐200 had worse measures of CDR‐SB and MMSE in EYO +5 years compared to pre‐200 carriers. EYO, estimated years to symptom onset; LME, linear mixed effect; MC, mutation carrier; NC, non‐carrier; CDR‐SB, Clinical Dementia Rating Sum of Boxes; MMSE, Mini‐Mental State Examination. Significance levels: *p < 0.05, **p < 0.005, ***p < 0.0005 in blue for pre‐200 versus NC, in red for post‐200 versus NC, and in black for pre‐200 versus post‐200 carriers.

CDR‐SB and MMSE measures showed differences in their cross‐sectional EYO associations between pre‐200 and post‐200 groups (Figure 6 and Table S9). In disease stages past the expected onset by 5 years, post‐200 MC showed worse CDR‐SB and MMSE scores compared to pre‐200 MC (CDR‐SB of 9.4 ± 0.4 for post‐200 versus 3.9 ± 0.6 for pre‐200, *p* value < 0.0001 and MMSE of 14.4 ± 0.8 for post‐200 vs. 23.8 ± 1.1 for pre‐200, *p* value < 0.0001). Post‐200 MCs also tended to have worse cognitive composite scores in later stages than pre‐200 MCs, although the difference was not statistically significant (−2.2 ± 0.2 for post‐200 vs. −1.7 ± 0.2 for pre‐200, *p* value = 0.05). Compared to NCs, post‐200 showed lower cognitive composite scores starting between EYO −5 and 0, whereas pre‐200 showed abnormal scores only beyond EYO = 0 (Figure 6 and Table S9).

#### 3.4.3 Mediation of clinical dementia deficit effects

Cortical regional Aβ burden partially mediated the mutation position effects on clinical measures starting at EYO = −5, except for six regions mostly in posterior areas (e.g., caudal and rostral anterior cingulate, cuneus, lingual, lateral occipital, and pericalcarine) which did not show any effects (e.g., average causal mediation effect at EYO = −5 for the lateral orbitofrontal: estimates = −0.14 [−0.26, −0.05] 95% CI, *p*‐value < 0.0001, Table S10 in supporting information). In subcortical regions, Aβ burden in amygdala, hippocampus, or putamen mediated these effects as well, with the putamen showing strong mediating effects starting even at EYO = −10 (e.g., average causal mediation effect at EYO = −10: estimates = −0.11 [−0.22, −0.03] 95% CI, *p*‐value < 0.0001, Table S10 in supporting information). Interestingly, the caudate and rostral anterior cingulate Aβ burden, which were the most influenced by mutation position with EYO, did not mediate the effects of mutation position on CDR‐SB. PMSD partially mediated the effect of mutation position on clinical dementia measure which was significantly worse in post‐200 MC in disease stages past the estimated years to symptom onset, at EYO = 0, 0.5, and 1 (e.g., average causal mediation effect at EYO = 0.5: estimates = 0.2 [0.01, 0.38] 95% CI, *p*‐value < 0.05, Table S11 in supporting information). The mutation position effect on clinical dementia measures was, however, not mediated by markers of white matter lesions, as measured by PV WMH volumes at any stage of the disease, although a trend was observed in disease stage EYO = 0 and over (eg, average causal mediation effect at EYO = 0.5: estimates = 0.08 [−0.01, 0.22] 95% CI, *p* = 0.08; Table S11 in supporting information). Similarly, these effects were not mediated by the total count of microhemorrhages (eg, average causal mediation effect at EYO = 0.5: estimates = 0.08 [−0.01, 0.22] 95% CI, *p* = 0.08; Table S11 in supporting information).

## 4 DISCUSSION

In this cross‐sectional neuroimaging and clinical study of a DIAD population, we demonstrated that mutation position within the *PSEN1* coding sequence influenced the spatiotemporal development of Aβ deposits and signs of SVD as well as the late clinical stages of the disease. Carriers with mutations postcodon 200 have elevated Aβ burden beginning in occipital areas very early in the disease, as well as more severe markers of SVD, including PV white matter lesions and diffusion indices, which partly mediate worse clinical outcomes in later stages compared to those with mutations precodon 200. The precodon 200 group began accumulating Aβ later in the disease course but reached higher levels in most cortical and subcortical regions, resulting in higher Aβ burden in later disease stages. Although this genotype grouping strategy has limitations, these findings highlight the genotypic variability seen in DIAD and how it plays a role in disease presentation and progression. These data may inform patient prognoses and the conduct of future clinical trials in this patient population.

We previously reported that mean cortical and striatal Aβ accumulation was higher in precodon 200 *PSEN1* carriers.^8^ Our present study further examined 40 cortical and subcortical regions to provide a more detailed evaluation. We found that, compared to post‐200 carriers, pre‐200 carriers had more rapid accumulation of Aβ in all 40 regions except for posterior regions such as the cuneus and lingual cortex. Though mutation position influenced Aβ burden trajectory, the regional patterns of Aβ burden were similar in both groups, with the highest accumulation in the caudate, precuneus, middle frontal, and rostral anterior cingulate and the least accumulation in the cuneus, lingual cortex, and thalamus. Such regional Aβ accumulation patterns have been widely reported in studies of DIAD, with regions such as the precuneus showing the earliest changes and being highly associated with disease progression, as well as high striatal uptake compared to sporadic LOAD populations.^39^, ^40^, ^41^, ^42^ While these reports combined all mutation types, *PSEN1* variants account for more carriers than *PSEN2* and *APP* mutation types combined and contribute significantly to the previously reported patterns of Aβ distribution and explain consistency across studies. Our finding, that the regional pattern of Aβ deposition is consistent across mutation groups but that the time course differs, nevertheless supports the use of summary regions for evaluating overall Aβ burden in DIAD and its utility in clinical trials.

While we observed a consistent Aβ pattern overall, interestingly, post‐200 carriers showed Aβ PET binding earlier in the disease starting in lingual and occipital regions, commonly affected later in the disease course.^43^ Recent work using large neuroimaging studies of LOAD defined three types of progression, including an occipital type where Aβ starts to accumulate in lingual and occipital regions and then propagates to the precuneus,^44^ which mimicked the profile found in post‐200 carriers. A similar data‐driven approach applied in DIAD cohorts would be of interest to confirm this pattern within different mutation types and subtypes. However, it is not clear whether this early occipital SUVR uptake observed 20 years ahead of expected onset in post‐200 carriers corresponds to Aβ deposition in the parenchyma or in blood vessels. Occipital regions are, indeed, often associated with vascular Aβ or CAA.^45^ Although the PiB signal from CAA may be overshadowed by the signal from parenchymal amyloid,^46^ considering that current Aβ PET tracers can detect both parenchymal plaques as well as Aβ in the vasculature,^47^ it is possible that this early occipital PiB uptake indicates CAA rather than parenchymal Aβ plaques.

This hypothesis is consistent with our finding that post‐200 carriers show elevated markers of SVD early, between EYO −10 and 0, and with prior imaging and neuropathology studies that clearly established more severe CAA in individuals with a *PSEN1* mutation postcodon 200.^9^, ^10^ We confirmed that post‐200 carriers had larger white matter lesions as measured by WMH volumes, and we found specifically larger volumes in posterior PV areas. Similar imaging findings were reported by Ryan and colleagues based on semi‐quantitative measure of WMH volume. They, however, reported greater inflammation in white matter with lower axonal density or integrity in pre‐200 cases, indicating worse white matter damage in this group at autopsy.^11^ This latter finding seems to contradict our in vivo findings of white matter injury. The cross‐sectional increase in PSMD, a reliable marker of SVD,^19^, ^37^, ^48^ was particularly high in the post‐200 group.

Regarding microhemorrhages, while we confirmed our previous report that mutation position does not significantly affect the odds of developing them generally^28^ or in any specific lobe, we did find an influence on the total count of microhemorrhages, including a trend toward occipital count, with post‐200 carriers showing higher counts starting at preclinical stages. This finding has implications for clinical trial enrollment since total microhemorrhage count is a strong risk factor for the development of Aβ‐related imaging abnormalities (ARIA) in individuals with DIAD treated with anti‐amyloid antibodies.^49^

Vascular risk factors may influence the development of these markers of SVD, but in our cohort no difference between pre‐ and post‐200 *PSEN1* carriers was observed in the prevalence of hypercholesterolemia, diabetes, or stroke. However, the prevalence of clinically confirmed hypertension was lower in the pre‐200 group. This might be explained by the younger mean age of this group, although the difference was not significant. The post‐200 *PSEN1* carrier group had a similar prevalence of hypertension compared to the NC group, so in our cohort having hypertension did not entirely explain the more severe SVD markers in this group.

In terms of clinical presentations, we found a trend toward higher prevalence of motor and behavioral symptoms in post‐200 compared to pre‐200, consistent with previous DIAN studies in which motor deficits, quantified with the Unified Parkinson Disease Rating Scale, were more common and more pronounced in post‐200 compared to pre‐200.^13^ The prevalence of other clinical, visual, or language impairments was not significantly different between groups, but, again, the post‐200 group tended to have higher prevalence of CDR > 0 for behavioral, comportment, and personality domains. We focused on carriers reported by their clinicians to have shown decline; thus, our sample size was small and limited our ability to rigorously examine group differences.

While behavioral and motor symptoms appeared less prevalent in the pre‐200 group, interestingly, the ages of onset of these reported symptoms were lower in this group. Based on their younger age at onset and rapid development of Aβ deposition,^8^, ^50^ one might expect individuals with mutations precodon 200 to experience worse clinical and cognitive symptoms compared to individuals with a mutation postcodon 200. However, even after accounting for age of onset and other potential cofounding factors like *APOE* ɛ4 status, we still observed that post‐200 carriers presented with more severe clinical dementia ratings and MMSE scores and tended to show worse cognitive composite scores in later disease stages compared to pre‐200. We previously reported similar cross‐sectional overall progression of CDR‐SB measures between both groups, suggesting similar functional decline overall.^8^ Differences in modeling and sample size may explain the difference in findings. However, further evaluation utilizing grouping by cytoplasmic versus transmembrane mutation location found an effect on cognition as measured by MMSE, confirming that the mutation position influences cognitive decline.^51^

We further hypothesized that the mutation position effect on clinical measures might be linked to the worse markers of SVD in the post‐200 group. We found that PSMD, and a trend for PV WMH volumes, partially mediated the worse clinical outcomes in late disease stages. We also observed that regional amyloid burden mediated the effects of mutation position on clinical measures. Within the scope of our study, we did not investigate how both regional amyloid and SVD mediations entered into play; however, others have shown that Aβ mediates the relationship between SVD and cognition in LOAD, suggesting close relationships among these factors.^52^ We focused on markers of amyloidosis and SVD, but other factors mediating or contributing to these effects, such as axonal degeneration, may also play a role.

A limitation of using codon 200 to investigate genetic variability is the arbitrary selection of the codon location without clear biological relevance and the inherent issue of dependency on the cohort size and number of mutations represented in each group. One could speculate that mutations located postcodon 200, being closer to the two aspartate residues critical for gamma‐secretase activity, may have differential effects on APP processing. Other grouping methods accounting for protein domains and ongoing investigations into mechanistic pathways involving *PSEN1* may provide additional insight into why certain mutations located precodon 200 appear more deleterious than certain mutations located postcodon 200. Further studies are needed to understand the biology underlying such mutation‐specific AD pathophysiology processes.

In summary, this study highlights better understanding of the risk factors of certain families for developing more aggressive pathologies is critically important for enabling a more adapted response in clinical trials, especially if certain families with *PSEN1* post‐200 mutations are at greater risk of developing ARIA. The heterogeneity in disease presentation in DIAD needs to be considered in trial design, participant inclusion/exclusion criteria, and adaptive trial monitoring.

## CONSENT STATEMENT

All participants or their caregivers provided written informed consent approved by their local institution's review board.

## CONFLICT OF INTEREST STATEMENT

NJM reports receiving a travel fellowship from the Alzheimer's Association to present related work at the Alzheimer Imaging Consortium preconference and the Alzheimer's Association International Conference. CC has received research support from GSK and Eisai Co., Ltd., and acknowledges travel support from Somalogics and consulting fees from Circular Genomics and Alector. CC is a member of the advisory board of Vivid Genetics and Circular Genomics and owns stock in those companies. DMC reports travel support from the Alzheimer's Association. GSD reports consulting fees from Parabon Nanolabs, honoraria from PeerView Media, Continuing Education Inc, Eli Lilly and Company, expert testimony fees from Barrow and from DynaMed, material support from Horizon Therapeutics and Avid Radiopharmaceuticals, and owing stock in ANI Pharmaceuticals and Parabon Nanolabs. NCF has provided consultancy or served on advisory or data safety and monitoring boards for F. Hoffmann‐La Roche, Ltd. and Eli Lilly and Company, Ionis, Biogen, and Siemens. WEK is a co‐inventor on a patent portfolio related to PiB PET technology owned by the University of Pittsburgh. GE Healthcare holds a license agreement with the University of Pittsburgh based on the technology described in this manuscript. Dr. Klunk is a co‐inventor of PiB and, as such, has a financial interest in this license agreement. GE Healthcare provided no grant support for this study and had no role in the design or interpretation of results or preparation of this manuscript. WEK has served on the data safety monitoring board for Biogen and owns stock in Cognoptix. JL reports consulting fees and/or honoraria from Eisai Co., Ltd., Biogen, Bayer Vital, TEVA, F. Hoffmann‐La Roche, Ltd., and Zambon, participation on an advisory board for Axon Neuroscience. JMR reports research support from Avid Radiopharmaceuticals. AJS has served on advisory committees for Eisai Co., Ltd., and Siemens Medical Solutions USA, Inc., and has received material from Avid Radiopharmaceuticals, a subsidiary of Eli Lilly and Company. PRS is the company director for Neuroscience Research Australian Foundation (NeuRA), the Health‐Science Alliance, Schizophrenia Research Institute, Australian Association of Medical Research Institutes, Australia Dementia Network Ltd., Standing Pty Ltd., and Australasian Neuroscience Society and acknowledges receiving consulting fees from NeuRA. IY reports consulting fees from ABX‐CRO and Blue Earth Diagnostics, honoraria from Piramal, and travel supports from the Society of Nuclear Medicine and Molecular Imaging (SNMMI) and the European Association of Nuclear Medicine, for both of which he has served on committee. AMB reports consulting fees from Cognition Therapeutics and Regeneron, honoraria from Yale University, Celdara Medical, International Neuropsychological Society, Pennington Biomedical Research Center, International Society for Neurovascular Disease, American College of Neuropsychopharmacology, American Neuropathologists, travel support from the International Neuropsychological Society. AMB has served on an advisory board for the Albert Einstein College of Medicine and CogState and is an editor for *Alzheimer's & Dementia*. JCM reports consulting fees from Barcelona Brain Research Center (BBRC), Native Alzheimer Disease‐Related Resource Center in Minority Aging Research, and honoraria from Montefiore Grand Rounds and Tetra‐Inst ADRC seminar series, Grand Rounds. JCM has served on advisory and/or study monitoring boards for Cure Alzheimer's Fund, Diverse Vascular Contributions to Cognitive Impairment and Dementia (VCID) Observational Study and the Longitudinal Early‐Onset Alzheimer's Disease Study (LEADS). EM is a co‐inventor of a test licensed by C2N Diagnostics and reports honoraria from Eisai Co., Ltd., and the American Academy of Neurology (AAN), travel support from the Alzheimer's Association, Alnylum, Fondation Alzheimer, Amsterdam UMC, and F. Hoffmann‐La Roche, Ltd. EM has served on advisory boards for Eli Lilly and Company, the NIA, Alector, Cumulus Neuroscience Ltd., and SAGE Therapeutics and has leadership involvement in Fondation Alzheimer and Alzamend. CX reports consulting fees from Diadem, participation on the FDA Advisory Committee on Imaging Medical Products, and other financial interests with C2N Diagnostics unrelated to the current study. RJB is the Principal Investigator of the DIAN Observational Study; receives research support from the NIA of the NIH, DIAN‐TU Trial Pharmaceutical Partners, and DIAN‐TU Pharma Consortium; has equity ownership interest in C2N Diagnostics; receives royalty income based on technology licensed by Washington University to C2N Diagnostics; has received honoraria from the Korean Dementia Association, American Neurological Association, Fondazione Prada, Weill Cornell Medical College, and Harvard University, travel support from Alzheimer's Association Roundtable, BrightFocus Foundation, Duke Margolis Alzheimer's Roundtable, Fondazione Prada, F. Hoffmann‐La Roche, Ltd., NAPA Advisory Council on Alzheimer's Research, and Tau Consortium Investigator's Meeting; and has received income from C2N Diagnostics for serving on the scientific advisory board and drugs and services from Eisai Co., Ltd., Janssen, and F. Hoffmann‐La Roche, Ltd. for the DIAN‐TU Next Generation and Open Label Extension trials. JPC reports consulting fees from Humana, MedaCorp, and ExpertConnect. TLSB reports consulting fees, honoraria, and/or advisory board compensation from Biogen, Eli Lilly and Company, Eisai Co., Ltd., Siemens, Bristol Myers Squibb, and technology transfer and precursors for radiopharmaceuticals from Avid Radiopharmaceuticals, LMI, and Cerveau/Lantheus. TLSB reports participation in the ASNR Alzheimer's and ARIA Study Group, QIBA Amyloid PET Working Group, Alzheimer's Association Clinical Tau PET Work Group, and the American College of Radiology/AlzNet Work Group. The other authors report no conflicts of interest. Author disclosures are available in the supporting information.

## Supporting information



Supporting Information

Supporting Information

## DIAN COLLABORATORS

### 1

**Table:**

Name	Institution	Role	Contribution
Martin Farlow	Indiana University	Site Leader	Coordinated imaging data from site
Alison Goate	Icahn School of Medicine at Mount Sinai	Genetic Core Co‐Leader	Co‐Led and coordinated all genetic data
Jason Hassenstab	Washington University School of Medicine in Saint Louis	Cognition Core Leader	Led and coordinated all cognition data
Ted Huey	Butler Hospital	Site Leader	Led and coordinated imaging data from site
Takeshi Ikeuchi	Niigata University	Site Leader	Led and coordinated imaging data from site
Robert Koeppe	Michigan University	Subcontract PI (PET)	Led and coordinated PET imaging acquisition and harmonization
Jae‐Hong Lee	Asan Medical Center, South Korea	Site Leader	Led and coordinated imaging data from site
Ralph Martins	Edith Cowan University	Site Leader	Led and coordinated imaging data from site
Alan E. Renton	Icahn School of Medicine at Mount Sinai	Genetic Core Co‐Leader	Co‐Led and coordinated all genetic data
