# Supplementary material for: Presenilin‐1 mutation position influences amyloidosis, small vessel disease, and dementia with disease stage
Source: Alzheimers Dement. 2024 Feb 21;20(4):2680–97. doi: 10.1002/alz.13729 (PMC11032566; doi:10.1002/alz.13729)
Supplement: Supplementary file 1 — Supporting Information [file ALZ-20-2680-s001.pdf]

Supplementary Material

Supplement to: Joseph-Mathurin N, et al. Presenilin-1 mutation position influences amyloidosis, small vessel disease, and dementia with disease stage

Table of Contents

1. Dominantly Inherited Alzheimer Network Consortium – additional study members 2

2. Statistical analysis models and equations 5

2.1. Linear Mixed Effect Models, Sample Size, and EYO range grouping 5

2.1.1. Regional PiB-uptake and EYO 5

2.1.2. Regional WMH volumes and EYO 5

2.1.3. Peak width of Skeletonized Mean Diffusivity and EYO 6

2.1.4. Cognition and EYO 6

2.2. Negative Binomial models (Two-part models) for count and zero-inflated variables 7

2.2.1. Deep WMH volumes 7

2.2.2. Microhemorrhages 7

2.3. Mediation Analysis 8

2.3.1. Markers of regional Amyloid as mediator 8

2.3.2. Markers of SVD as mediator 9

3. Supplementary Figures and Tables 10

Figure S1 – Flow diagram of cohort selection, exclusion criteria, and analyses 10

Tables S1 - Baseline characteristics of participants per mutation status and sub cohort study 11

Tables S2 - Mutation by EYO effect on PiB SUVR per regions 12

Table S3a – LME Estimates of mean difference SUVR per EYO range in all 40 regions for Pre-200 MC versus NC 13

Table S3b – LME Estimates of mean difference SUVR per EYO range in all 40 regions for Post-200 MC versus NC 14

Table S3c – LME Estimates of mean difference SUVR per EYO range in all 40 regions for pre-200 MC versus post-200 MC 15

Table S4 – LME results mutation by EYO effect on SVD measures (Global PSMD, total, PV, Ant, and Post WMH volumes) 16

Table S5a – LME Estimates of mean difference in **Global SVD measures** per EYO range for pre-200 MC versus NC, post-200 MC versus NC, and pre-200 versus post-200 MC 17

Table S5b – LME Estimates of mean difference in **WMH Regions** per EYO range for pre-200 MC versus NC, post-200 MC versus NC, and pre-200 versus post-200 MC 17

Table S6a – Results of Negative binomial mixed effect models evaluating the effect of mutation position on **Deep WMH volumes per EYO** 18

Table S6b – Results of Negative binomial mixed effect models evaluating the effect of mutation position on **Deep WMH volumes per EYO range** 18

Table S7 – Microhemorrhages prevalence, count, and location per mutation group 19

Table S8 – Mutation position effect conditional of EYO on microhemorrhage count per region 20

Table S9 – LME Estimates of mean difference in **Clinical measures** per EYO range for pre-200 MC versus NC, post-200 MC versus NC, and pre-200 versus post-200 MC 21

Table S10 – Mediation Analyses conditional per EYO with individual PiB regions as mediator 22

Table S11 – Mediation Analyses conditional per EYO with SVD markers as mediator 24

1. Dominantly Inherited Alzheimer Network Consortium – additional study members

Name	Institution	Affiliation
Graff-Radford, Neill R.	Mayo	Mayo Clinic , Department of Neurology, Jacksonville, Florida
Chrem Mendez, Patricio	Fleni	Department of Cognitive Neurology, Institute for Neurological Research Fleni, Buenos Aires, Argentina
Surace, Ezequiel	Fleni	Department of Molecular Biology and Neuropathology, Institute for Neurological Research Fleni, Buenos Aires, Argentina
Ikonomovic, Snezana	Pitt	University of Pittsburgh, Department of Neurology
Nadkarni, Neelesh K.	Pitt	University of Pittsburgh, Departments of Medicine (Geriatric Medicine) and Neurology
Lopera, Francisco	Medellin Colombia	Grupo de Neurociencias de Antioquia (GNA), Universidad de Antioquia, Medellin, Colombia
Ramirez, Laura	Medellin Colombia	Grupo de Neurociencias de Antioquia (GNA), Universidad de Antioquia, Medellin, Colombia
Aguillon, David	Medellin Colombia	Grupo de Neurociencias de Antioquia (GNA), Universidad de Antioquia, Medellin, Colombia
Leon, Yudy	Medellin Colombia	Grupo de Neurociencias de Antioquia (GNA), Universidad de Antioquia, Medellin, Colombia
Ramos, Claudia	Medellin Colombia	Grupo de Neurociencias de Antioquia (GNA), Universidad de Antioquia, Medellin, Colombia
Alzate, Diana	Medellin Colombia	Grupo de Neurociencias de Antioquia (GNA), Universidad de Antioquia, Medellin, Colombia
Baena, Ana	Medellin Colombia	Grupo de Neurociencias de Antioquia (GNA), Universidad de Antioquia, Medellin, Colombia
Londono, Natalia	Medellin Colombia	Grupo de Neurociencias de Antioquia (GNA), Universidad de Antioquia, Medellin, Colombia
Moreno, Sonia	Medellin Colombia	Grupo de Neurociencias de Antioquia (GNA), Universidad de Antioquia, Medellin, Colombia
Laske, Christoph	Tubingen	German Center for Neurodegenerative Diseases Section for Dementia Research, Hertie-Institute for Clinical Brain Research and Department of Psychiatry and Psychotherapy
Kuder-Buletta, Elke	Tubingen	German Center for Neurodegenerative Diseases
Graber-Sultan, Susanne	Tubingen	German Center for Neurodegenerative Diseases
Preische, Oliver	Tubingen	German Center for Neurodegenerative Diseases Section for Dementia Research, Hertie-Institute for Clinical Brain Research and Department of Psychiatry and Psychotherapy
Hofmann, Anna	Tubingen	German Center for Neurodegenerative Diseases Hertie-Institute for Clinical Brain Research
Kasuga, Kensaku	Niigata	Niigata University
Ishii, Kenji	Tokyo	Tokyo Metropolitan Institute of Gerontology
Senda, Michio	Kobe	Kobe City Medical Center General Hospital

Sanchez-Valle, Raquel	Barcelona	Alzheimer's disease and other cognitive disorders Unit, Neurology Service, Hospital Clinic de Barcelona
Rosa-Neto, Pedro	McGill-Canada	
Roh, Jee Hoon	Seoul Korea	Korea University College of Medicine
Riddle, Meghan C.	Butler	Butler Hospital, Warren Alpert School of Medicine at Brown University
Menard, William	Butler	Butler Hospital, Warren Alpert School of Medicine at Brown University
Bodge, Courtney	Butler	Butler Hospital, Warren Alpert School of Medicine at Brown University
Surti, Mustafa	Butler	Butler Hospital, Warren Alpert School of Medicine at Brown University
Takada, Leonel Tadao	Sao Paulo	Hospital das Clinicas, University of Sao Paulo School of Medicine
Sanchez-Gonzalez, VJ	Guadalajara	Doctorado en Biociencias & Departamento de Clinicas, Centro Universitario de Los Altos, UDG
Orozco-Barajas, Maribel	Guadalajara	Doctorado en Biociencias & Departamento de Salud, Centro Universitario de Los Altos, UDG
Esposito, Bianca T.		Department of Genetics and Genomic Sciences and Ronald M. Loeb Center for Alzheimer's Disease, Icahn School of Medicine at Mount Sinai, New York, NY 10029
Marsh, Jacob	Washington in St. Louis School of Medicine	Department of Psychiatry, Washington University in St. Louis
Fernandez, Victoria	Washington University	1)Department of Psychiatry, Washington University in St. Louis, Mo, USA 2)NeuroGenomics and Informatics Center, Washington University School of Medicine, St. Louis, MO, USA
Fagan, Anne M.	Washington University	Department of Neurology, Washington University in St. Louis
Jerome, Gina	Washington University	Department of Neurology, Washington University in St. Louis
Herries, Elizabeth	Washington University	Department of Neurology, Washington University in St. Louis
Levey, Allan I.	Emory University	Goizueta Alzheimer's Disease Research Center, Emory University, Atlanta, GA 30329
Johnson, Erik C.B.	Emory University	Goizueta Alzheimer's Disease Research Center, Emory University, Atlanta, GA 30329
Seyfried, Nicholas T.	Emory University	Goizueta Alzheimer's Disease Research Center, Emory University, Atlanta, GA 30329
Bechara, Jacob A	Sydney	Neuroscience Research Australia, Sydney NSW 2031 Australia
Franklin, Erin E.	Washington University	Department of Pathology and Immunology, Washington University in St. Louis
Flores, Shaney	Washington University	Washington University School of Medicine in St. Louis
Hantler, Nancy	Washington University	Washington University School of Medicine in St. Louis
Jarman, Steve	Washington University	Washington University School of Medicine in St. Louis

Koudelis, Deborah	Washington University	Washington University School of Medicine in St. Louis
Nicklaus, Joyce	Washington University	Washington University School of Medicine in St. Louis
Pulizos, Christine	Washington University	Washington University School of Medicine in St. Louis
Mishall, Sheetal	Washington University	Washington University School of Medicine in St. Louis
Sabaredzovic, Edita	Washington University	Washington University School of Medicine in St. Louis
Deng, Emily	Washington University	Washington University School of Medicine in St. Louis
Candela, Madison	Washington University	Washington University School of Medicine in St. Louis
Smith, Hunter	Washington University	Washington University School of Medicine in St. Louis
Hobbs, Diana	Washington University	Washington University School of Medicine in St. Louis
Scott, Jalen	Washington University	Washington University School of Medicine in St. Louis
Xu, Xiong	Washington University	Washington University School of Medicine in St. Louis
Gremminger, Emily	Washington University	Washington University School of Medicine in St. Louis
Bui, Ryan	Washington University	Washington University School of Medicine in St. Louis
Sosa Ortiz, Ana Luisa	Mexico City	
Daniels, Alisha	Washington University	Washington University School of Medicine in St. Louis
Courtney, Laura	Washington University	Washington University School of Medicine in St. Louis
Mori, Hiroshi		Osaka Metropolitan University
Xu, Jinbin	Washington University	Department of Radiology, Washington University in St. Louis
Barthelemy, Nicolas	Washington University	Department of Neurology, Washington University in St. Louis
Smith, Jennifer	Washington University	Department of Neurology, Washington University in St. Louis

2. Statistical analysis models and equations

2.1. Linear Mixed Effect Models, Sample Size, and EYO range grouping

All linear mixed effects models were ran using SAS software. We run the analysis with EYO as continuous and as categorical measures per range of 5years or 10 years.

2.1.1. Regional PiB-uptake and EYO

To evaluate the effects of mutation position on regional amyloid burden as a function of estimated years to symptom onset (EYO), we used the *model (1)* and *model (2),* accounting or not for age of onset, respectively. Both models included *APOE-ε4* status (presence or not of at least one *APOE-ε4* allele), sex at birth, and education in years as fixed effects and family cluster as random effect. The linear mixed effect model below was applied and run individually for each FreeSurfer region of interest (ROI, n=40) and results of the model were corrected for multiple comparisons using the Benjamin-Hochberg method (Benjamini and Hochberg 1995).

(1) $PiB\ SUVR_{ROI_n} \sim \beta_0 + \beta_1 \times (Mutation\ position \times EYO) + \beta_2 \times mutation\ position + \beta_3 \times EYO + \beta_4 \times APOEe4\ status + \beta_5 \times education + \beta_6 \times sex + \beta_7 \times age\ of\ onset$

(2) $PiB\ SUVR_{ROI_n} \sim \beta_0 + \beta_1 \times (Mutation\ position \times EYO) + \beta_2 \times mutation\ position + \beta_3 \times EYO + \beta_4 \times APOEe4\ status + \beta_5 \times education + \beta_6 \times sex$

These 2 models were used with EYO as a categorical variable to estimate $Mean_{PIB\ SUVR_{ROI_n}} \pm Standard\ Error$ for each group (NC, Pre-200, and Post-200 MC) and compare the groups at different disease stage as ranged per 5years (8 EYO categories: beyond -25, -25 to -20, -20 to -15, -15 to -10, -10 to -5, -5 to 0, 0 to +5, above +5). The total sample size with non-missing PIB-PET data and other variables included in model (1) and (20) = 350 and was repartitioned as follow for the 3 groups and 8 EYO categories:

**PiB**	**< -25**	**-25 to -20**	**-20 to -15**	**-15 to -10**	**-10 to -5**	**-5 to 0**	**0 to +5**	**> +5**
NC	15	17	30	18	17	20	13	11
Pre	6	4	10	9	14	6	21	6
Post	14	10	22	14	17	22	22	13

2.1.2. Regional WMH volumes and EYO

To evaluate the effects of mutation position on total and regional white matter hyperintensity volumes as a function of EYO, we used the *model (3)* and *model (4)*, accounting or not for age, respectively. Both models included *APOE-ε4* status, sex at birth, mean arterial pressure (MAP), and education in years as fixed effects and family cluster as random effect. The linear mixed effect model below was applied and run individually for each WMH volume (Vol, n=4). Due to the small number of regions and the exploratory nature of the analysis, the results were not adjusted for multiple comparisons.

(3) $WMH_{Vol_n} \sim \beta_0 + \beta_1 \times (Mutation\ position \times EYO) + \beta_2 \times mutation\ position + \beta_3 \times EYO + \beta_4 \times APOEe4\ status + \beta_5 \times education + \beta_6 \times sex + \beta_7 \times MAP + \beta_8 \times age$

(4) $WMH_{Vol_n} \sim \beta_0 + \beta_1 \times (Mutation\ position \times EYO) + \beta_2 \times mutation\ position + \beta_3 \times EYO + \beta_4 \times APOEe4\ status + \beta_5 \times education + \beta_6 \times sex + \beta_7 \times MAP$

These 2 models were used with EYO as a categorical variable to estimate $Mean_{WMH_{Vol_n}} \pm Standard\ Error$ for each group (NC, Pre-200, and Post-200 MC) and compare the groups at different disease stage as ranged per

10years (3 EYO categories: beyond -10, -10 to 0, above 0). The total sample size with non-missing sub parcellation of WMH data and variable included in model (3) and (4) = 165 and was repartitioned as follow for the 3 groups and 3 EYO categories. When categorized by EYO range of 5, some group sample sizes were n<4.

**Regional WMH (semi-automated pipeline)**	**< -10**	**-10 to 0**	**> 0**
NC	31	22	11
Pre	15	10	14
Post	27	15	20

2.1.3. Peak width of Skeletonized Mean Diffusivity and EYO

To evaluate the effects of mutation position on global white matter injury (as measured by peak width of skeletonized mean diffusivity (PSMD)) as a function of EYO, we used the *model (5)* and *model (6)*, accounting or not for age, respectively. Both models included *APOE-ε4* status, sex at birth, MAP, and education in years as fixed effects and family cluster as random effect.

(5) $PSMD \sim \beta_0 + \beta_1 \times (Mutation\ position \times EYO) + \beta_2 \times mutation\ position + \beta_3 \times EYO + \beta_4 \times APOEe4\ status + \beta_5 \times education + \beta_6 \times sex + \beta_7 \times MAP + \beta_8 \times age$

(6) $PSMD \sim \beta_0 + \beta_1 \times (Mutation\ position \times EYO) + \beta_2 \times mutation\ position + \beta_3 \times EYO + \beta_4 \times APOEe4\ status + \beta_5 \times education + \beta_6 \times sex + \beta_7 \times MAP$

These 2 models were used with EYO as a categorical variable to estimate $Mean_{PSMD} \pm Standard\ Error$ for each group (NC, Pre-200, and Post-200 MC) and compare the groups at different disease stage as ranged per 10years (3 EYO categories: beyond -10, -10 to 0, above 0). The total sample size with non-missing PSMD data and other variables included in model (5) and (6) = 190 and was repartitioned as follow for the 3 groups and 3 EYO categories. When categorized by EYO range of 5, some group size were n<4.

**PSMD**	**< -10**	**-10 to 0**	**> 0**
NC	42	22	13
Pre	20	8	14
Post	32	11	28

2.1.4. Cognition and EYO

Because our clinical measures do not follow a linear progression with EYO, we investigate the effects of mutation position on cognition and clinical measures with disease stage by categorizing EYO by range of 5years. We used the *model (7)* and *model (8)*, accounting or not for age of onset, respectively. Both models included *APOE-ε4* status, sex at birth, and education in years as fixed effects and family cluster as random effect. The linear mixed effect model below was applied and run individually for each clinical and cognitive measures (n=3). Due to the small number of comparisons and the exploratory nature of the analysis, the results were not adjusted for multiple comparisons.

(7) $Clin/Cog \sim \beta_0 + \beta_1 \times (Mutation\ position \times EYOcat) + \beta_2 \times mutation\ position + \beta_3 \times EYOcat + \beta_4 \times APOEe4\ status + \beta_5 \times education + \beta_6 \times sex + \beta_7 \times age\ of\ onset$

(8) $Clin/Cog \sim \beta_0 + \beta_1 \times (Mutation\ position \times EYOcat) + \beta_2 \times mutation\ position + \beta_3 \times EYOcat + \beta_4 \times APOEe4\ status + \beta_5 \times education + \beta_6 \times sex$

These 2 models were used with EYO as a categorical variable to estimate $Mean_{Clin/Cog} \pm Standard\ Error$ for each group (NC, Pre-200, and Post-200 MC) and compare the groups at different disease stage as ranged per 5years (8 EYO categories: beyond -25, -25 to -20, -20 to -15, -15 to -10, -10 to -5, -5 to 0, 0 to +5, beyond +5). The total sample size with non-missing CDR-SB, MMSE, or Composite cognitive data and other variables included in model (7) and (8) = 391, 387, or 376 and was repartitioned as follow for the 3 groups and 8 EYO categories:

CDRSB	**< -25**	**-25 to -20**	**-20 to -15**	**-15 to -10**	**-10 to -5**	**-5 to 0**	**0 to +5**	**> +5**
NC	15	17	30	19	20	22	37	17
Pre	7	4	11	9	14	8	22	7
Post	11	16	22	16	21	22	37	17

MMSE	**< -25**	**-25 to -20**	**-20 to -15**	**-15 to -10**	**-10 to -5**	**-5 to 0**	**0 to +5**	**> +5**
NC	14	17	30	19	20	21	13	11
Pre	7	4	11	9	14	8	22	7
Post	11	16	21	16	21	22	37	16

CompCog	**< -25**	**-25 to -20**	**-20 to -15**	**-15 to -10**	**-10 to -5**	**-5 to 0**	**0 to +5**	**> +5**
NC	14	17	30	19	20	21	13	11
Pre	7	4	11	9	14	8	20	7
Post	11	16	21	16	21	21	35	10

2.2. Negative Binomial models (Two-part models) for count and zero-inflated variables

Two-part models using the glmmTMB package in R (github.com/glmmTMB/glmmTMB) were used to run zero inflated negative binomial models for microhemorrhages and DWMH variables that had a zero-inflated distribution. The model rendered one part for the binary outcome (0 or non-0), and a second part for the continuous data >0 outcome [29].

2.2.1. Deep WMH volumes

Similarly, as *model (3)* and *model (4), model (9)* and *model (10)* evaluated the effects of mutation position on deep white matter lesions as a function of estimated years to symptom onset (EYO), accounting or not for age, respectively.

(9) $DWMH \sim \beta_0 + \beta_1 \times (Mutation\ position \times EYO) + \beta_2 \times mutation\ position + \beta_3 \times EYO + \beta_4 \times APOEe4\ status + \beta_5 \times education + \beta_6 \times sex + \beta_7 \times MAP + \beta_8 \times age$

(10) $DWMH \sim \beta_0 + \beta_1 \times (Mutation\ position \times EYO) + \beta_2 \times mutation\ position + \beta_3 \times EYO + \beta_4 \times APOEe4\ status + \beta_5 \times education + \beta_6 \times sex + \beta_7 \times MAP$

2.2.2. Microhemorrhages

Similar approach evaluated the effects of mutation position on microhemorrhages in the entire brain or five individual ROIs (frontal, parietal, temporal, occipital, and deep) as a function of estimated years to symptom onset (EYO), accounting or not for age.

(11) $mH\ count_{ROI_n} \sim \beta_0 + \beta_1 \times (Mutation\ position \times EYO) + \beta_2 \times mutation\ position + \beta_3 \times EYO + \beta_4 \times APOEe4\ status + \beta_5 \times education + \beta_6 \times sex + \beta_7 \times MAP + \beta_8 \times age$

(12) $mH\ count_{ROI_n} \sim \beta_0 + \beta_1 \times (Mutation\ position \times EYO) + \beta_2 \times mutation\ position + \beta_3 \times EYO + \beta_4 \times APOEe4\ status + \beta_5 \times education + \beta_6 \times sex + \beta_7 \times MAP$

We fit the mixed effect model the relationship between mH and mutation position. The distribution of count is modeled as the zero-inflated negative binomial distribution to account for its dispersion and mass on zero. The effect of mutation position is adjusted for EYO, its interaction with EYO, *APOE-ε4* status, education, sex, age and MAP. The intra-correlation within families is modeled by the random effect. In the second model of each mH outcome, we remove age from the model.

2.3. Mediation Analysis

To evaluate potential indirect effect of mutation position on clinical measures, via markers of small vessel disease. We used the lme4 and Mediation packages in R:

cran.r-project.org/web/packages/mediation/vignettes/mediation.pdf

Each PiB SUVR regions (n=40) or markers of small vessel disease that showed a significant effect in LME analyses were further evaluated in individual mediation analyses. For the approach, the mutation position corresponded to the independent variable, the clinical measure the dependent variable, and the regional PiB SUVR or marker of small vessel disease the mediator. The models included *APOE-ε4* status, sex at birth, and education in years as fixed effects and family cluster as random effect. We tested the significance of this indirect effect using bootstrapping procedures. Unstandardized indirect effects were computed for each of 1000 bootstrapped samples, and the 95% confidence interval was computed by determining the indirect effects at the 2.5th and 97.5th percentiles. For both evaluations (regional amyloid or SVD as mediator) EYO is treated as continuous for conditional mediation at EYO=-15, -10, -5, 0, 0.5, and 1. Mediation analyses were corrected for multiple test when evaluating the 40 PiB SUVR regions.

2.3.1. Markers of regional Amyloid as mediator

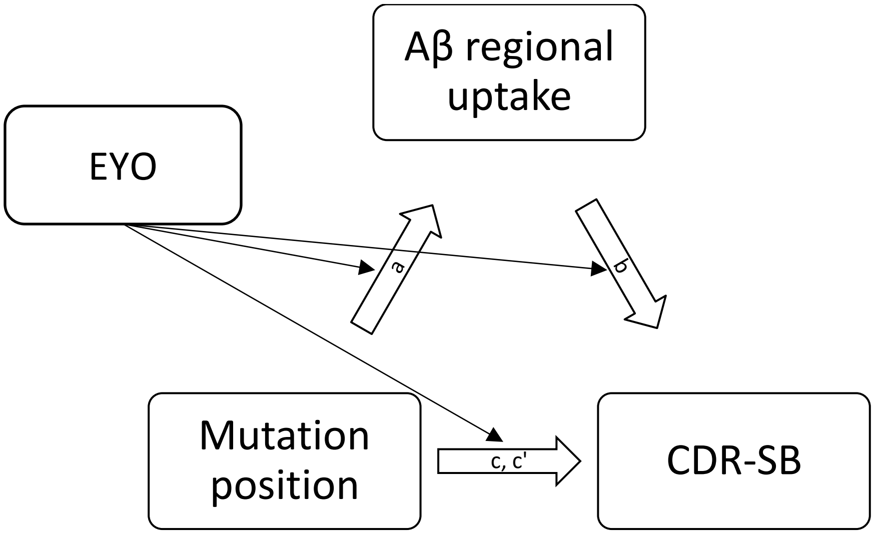

a $PiB\ SUVR \sim \beta_0 + \beta_1 \times (Mutation\ position \times EYO) + \beta_3 \times EYO + \beta_4 \times APOEe4\ status + \beta_5 \times education + \beta_6 \times sex + \beta_7 \times age\ of\ onset$

Total effect (c) $CDRSB \sim \beta_0 + \boldsymbol{\beta_1} \times (Mutation\ position \times EYO) + \beta_2 \times mutation\ position + \beta_3 \times EYO + \beta_4 \times APOEe4\ status + \beta_5 \times education + \beta_6 \times sex + \beta_7 \times age\ of\ onset$

Mediator effect (b) $CDRSB \sim \beta_0 + \beta_1 \times PiB\ SUVR + \beta_2 \times mutation\ position + \beta_3 \times EYO + \beta_4 \times APOEe4\ status + \beta_5 \times education + \beta_6 \times sex + \beta_7 \times age\ of\ onset$

(c') $CDRSB \sim \beta_0 + \boldsymbol{\beta_1} \times (Mutation\ position \times EYO) + \beta_2 \times mutation\ position + \beta_3 \times EYO + \beta_4 \times APOEe4\ status + \beta_5 \times education + \beta_6 \times sex + \beta_7 \times age\ of\ onset + \boldsymbol{\beta_8} \times PiB\ SUVR$

2.3.2. Markers of SVD as mediator

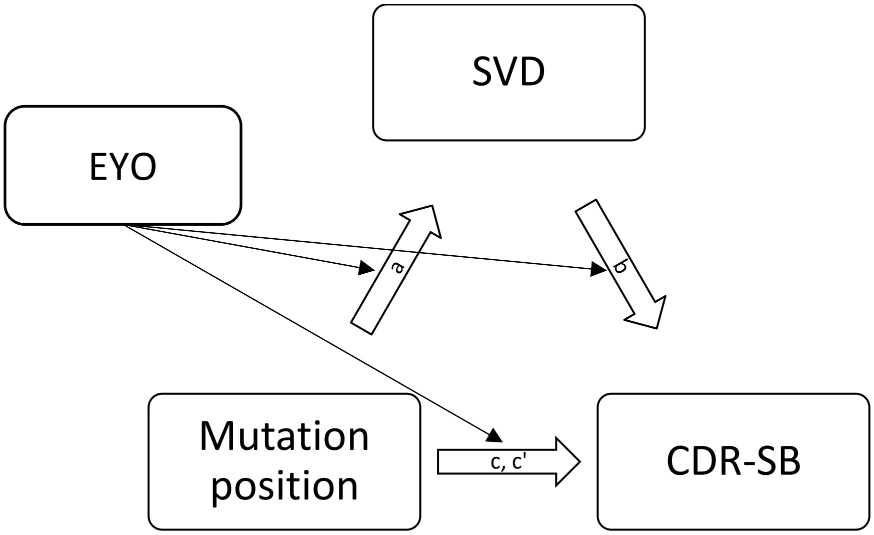

(a) $SVD \sim \beta_0 + \beta_1 \times (Mutation\ position \times EYO) + \beta_3 \times EYO + \beta_4 \times APOEe4\ status + \beta_5 \times education + \beta_6 \times sex + \beta_7 \times age\ of\ onset$

Total effect (c) $CDRSB \sim \beta_0 + \boldsymbol{\beta_1} \times (Mutation\ position \times EYO) + \beta_2 \times mutation\ position + \beta_3 \times EYO + \beta_4 \times APOEe4\ status + \beta_5 \times education + \beta_6 \times sex + \beta_7 \times age\ of\ onset$

Mediator effect (b) $CDRSB \sim \beta_0 + \beta_1 \times SVD + \beta_2 \times mutation\ position + \beta_3 \times EYO + \beta_4 \times APOEe4\ status + \beta_5 \times education + \beta_6 \times sex + \beta_7 \times age\ of\ onset$

(c') $CDRSB \sim \beta_0 + \boldsymbol{\beta_1} \times (Mutation\ position \times EYO) + \beta_2 \times mutation\ position + \beta_3 \times EYO + \beta_4 \times APOEe4\ status + \beta_5 \times education + \beta_6 \times sex + \beta_7 \times age\ of\ onset + \boldsymbol{\beta_8} \times SVD$

3. Supplementary Figures and Tables

Figure S1 – Flow diagram of cohort selection, exclusion criteria, and analyses

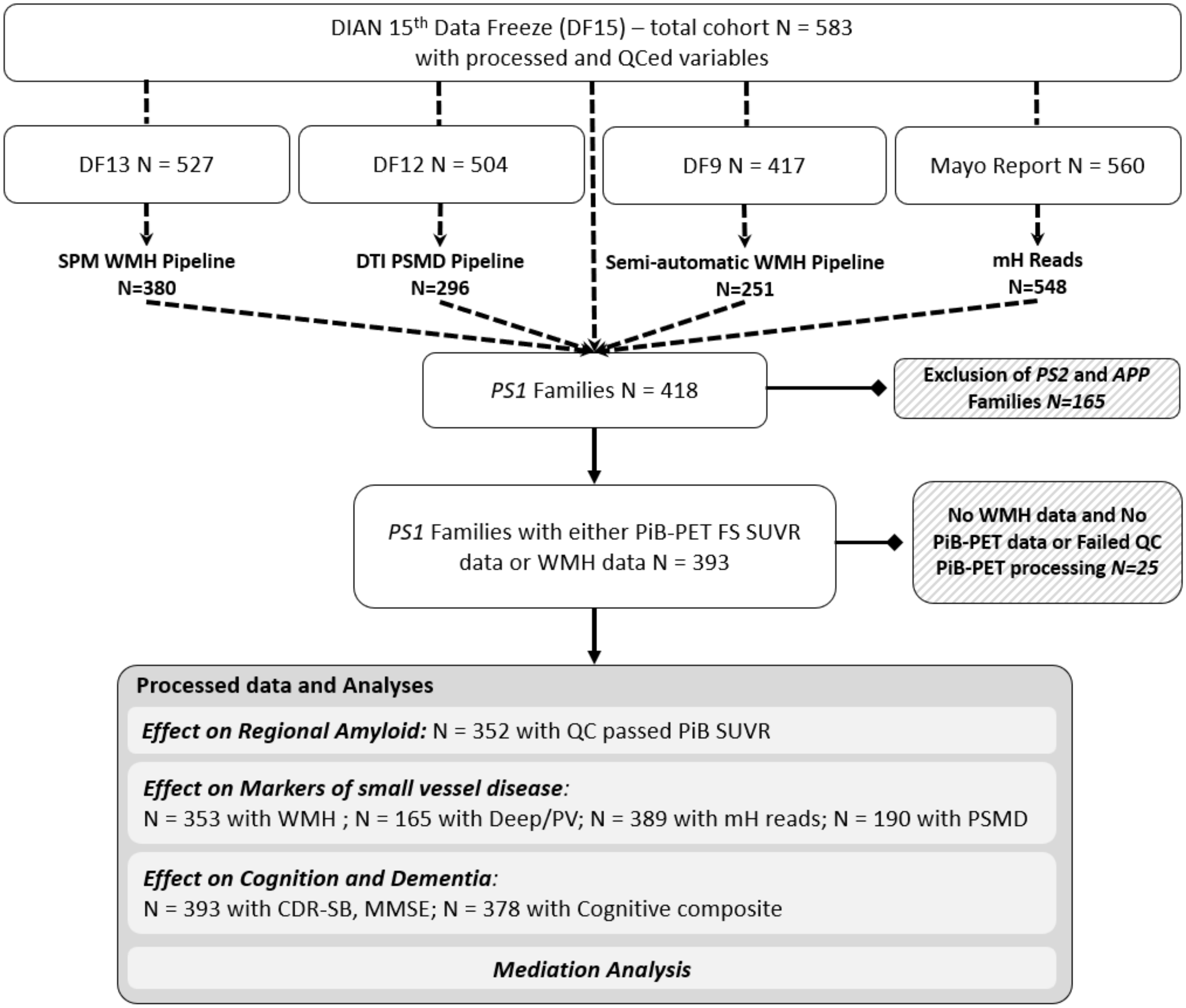

Annotations: DIAN = dominantly inherited Alzheimer network, PSEN1 = presenilin-1, PiB = Pittsburg Compound B, PET = positron emission tomography, SUVR = standardized uptake value ratio, FLAIR = fluid attenuated inversion recovery, NC = non-carriers, MC = mutation carrier, QC = quality control, SPM = statistical parametric mapping, WMH = white matter hyperintensity, mH = microhemorrhage, PSMD = peak width of skeletonized mean diffusivity, CDR-SB = clinical dementia rating sum of boxes, MMSE = mini-mental state examination.

Tables S1 - Baseline characteristics of participants per mutation status and sub cohort study

Group Mutation status	NC			Pre-200 MC			Post-200 MC		
Cohort/Subset Analyses	Overall	PSMD	Sub WMH	Overall	PSMD	Sub WMH	Overall	PSMD	Sub WMH
N (%)	148 (37.7)	77 (40.5)	64 (38.8)	83 (21.1)	42 (22.1)	39 (23.6)	162 (41.2)	71 (37.4)	62 (37.6)
Age, mean (SD), y	35.8 (10.6)	35.8 (11.2)	38.0 (11.3)	35.5 (9.3)	34.6 (10.8)	35.6 (10.3)	38.7 (11.6)	38.1 (12.2)	38.1 (12.0)
Female, n (%)	83 (56.1)	43 (55.8)	42 (65.6)	47 (56.6)	22 (52.4)	20 (51.3)	94 (58.0)	39 (54.9)	35 (56.5)
Education, mean (SD), y	15.1 (2.7)	15.4 (2.8)	15.2 (2.8)	14.8 (3.2)	15.0 (3.2)	14.4 (2.9)	14.2 (2.9)	14.0 (3.1)	14.5 (2.9)
APOE-ε4+ carriers, n (%)	43 (29.1)	20 (26.0)	18 (28.1)	16 (19.3)	9 (21.4)	7 (17.9)	54 (33.3)	24 (33.8)	16 (25.8)
Familial AAO, mean (SD), y	47.2 (7.3)	47.3 (7.1)	48.4 (7.0)	44.0 (9.0)	44.7 (9.0)	44.4 (9.3)	46.8 (7.2)	46.3 (7.0)	46.8 (7.3)
EYO, mean (SD), y	−11.01 (11.6)	−11.0 (12.9)	−10.0 (12.0)	−8.0 (11.0)	−9.7 (12.1)	−8.3 (11.8)	−7.6 (11.0)	−7.7 (11.7)	−8.3 (10.8)
EYO >0, n (%)	23 (15.5)	13 (16.9)	11 (17.2)	29 (34.9)	14 (33.3)	14 (35.9)	53 (32.7)	28 (39.4)	19 (30.6)
Asymptomatic, n (% of EYO>0)	-	-	-	2 (6.9)	1 (7.1)	1 (7.1)	0 (0)	0 (0)	0 (0)
CDR >0, n (%)	7 (4.7)	2 (2.6)	0 (0)	28 (33.7)	13 (31.0)	14 (35.9)	62 (38.3)	30 (42.3)	25 (40.3)
CDR-SB, mean (SD)	0.04 (0.18)	0.03 (0.1)	0.01 (0.1)	1.10 (2.02)	0.9 (1.8)	1.0 (1.8)	1.67 (3.53)	2.0 (3.7)	1.2 (2.9)
MMSE, mean (SD)	29.0 (1.2)	29.1 (1.2)	29.0 (1.3)	27.0 (4.3)	27.0 (4.7)	27.4 (4.0)	26.4 (5.8)	25.7 (6.3)	26.8 (5.0)
Cognitive composite, mean (SD)	0.06 (0.47)	0.08 (0.40)	0.08 (0.44)	-0.38 (1.03)	-0.30 (0.95)	-0.44 (1.05)	-0.50 (0.92)	-0.58 (0.96)	-0.49 (0.78)
PiB-PET Positive, n (%)	0 (0)	0 (0)	0 (0)	36 (43.4)	14 (35.9)	14 (35.9)	41 (25.3)	13 (21.0)	13 (21.0)
Mean Cortical PiB SUVR, mean (SD)	1.0 (0.1)	1.0 (0.1)	1.0 (0.1)	2.2 (1.3)	2.2 (1.4)	2.1 (1.4)	1.8 (0.9)	1.8 (0.7)	1.8 (0.8)
Stroke history, n (%) [a]	1 (0.6)	0 (0)	0 (0)	0 (0)	0 (0)	0 (0)	3 (1.9)	0 (0)	0 (0)
Systolic blood pressure, mean (SD), mmHg	122.6 (16.7)	123.1 (17.8)	123.0 (18.4)	121.8 (14.2)	122.8 (12.3)	121.9 (13.3)	123.2 (13.9)	122.3 (11.1)	124.0 (11.9)
Diastolic blood pressure, mean (SD), mmHg	76.1 (10.3)	76.2 (10.6)	76.2 (9.9)	73.7 (10.7)	75.8 (8.7)	73.1 (8.6)	76.3 (9.8)	75.6 (8.5)	76.5 (8.0)
Mean arterial blood pressure, mean (SD), mmHg	91.6 (11.5)	91.8 (12.2)	91.8 (12.0)	89.7 (11.0)	91.5 (9.1)	89.3 (9.4)	91.9 (9.9)	91.2 (8.3)	92.4 (8.1)
Hachinski Ischemia score, mean (SD)	0.18 (0.45)	0.16 (0.37)	0.24 (0.43)	0.18 (0.65)	0.12 (0.55)	0.20 (0.73)	0.45 (1.22)	0.25 (0.73)	0.15 (0.44)
Hypertension history, n (%)	23 (19.4)	13 (16.9)	15 (23.4)	3 (2.6)	0 (0)	1 (2.6)	19 (12.7)	6 (0.1)	3 (4.8) [†]
Hypercholesterolemia, n (%)	16 (10.8)	6 (0.1)	6 (0.1)	5 (6.0)	4 (0.1)	5 (13.2)	25 (15.4)	10 (16.1)	10 (16.1)
Diabetes, n (%)	5 (3.4)	2 (2.6)	3 (4.8)	2 (2.4)	0 (0)	0 (0)	2 (1.2)	1 (1.4)	0 (0)
Seizures, n (%)	2 (1.4)	0 (0)	0 (0)	4 (4.8)	2 (4.8)	2 (5.1)	6 (3.7)	3 (4.2)	4 (6.5)
Abnormal gait, n (%)	5 (3.4)	2 (2.6)	3 (4.7)	4 (4.8)	2 (4.8)	2 (5.1)	17 (10.5)	9 (9.9)	6 (9.8)
Tremor, n (%)	7 (4.7)	4 (5.2)	2 (3.1)	5 (6.0)	3 (7.1)	2 (5.1)	9 (5.6)	6 (8.5)	4 (6.5)

Annotations: NC = non-carriers, MC = mutation carrier, APOE = Apolipoprotein-E allele ε4, EYO = Estimated years to symptom onset, AAO = Age at onset, CDR-SB = clinical dementia rating sum of boxes, MMSE = mini-mental state examination, PiB = Pittsburg Compound B, PET = positron emission tomography, SUVR = standardized uptake value ratio.

Tables S2 - Mutation by EYO effect on PiB SUVR per regions

PiB SUVR Region	Pre-200 slope vs. NC slope			Post-200 slope vs. NC slope			Pre-200 slope vs. Post-200 slope		
	Estimate	SE	*P* ROI-adjusted	Estimate	SE	*P* ROI-adjusted	Estimate	SE	*P* ROI-adjusted
amygdala	0.01600	0.00302	0.00000	0.00441	0.00248	0.08303	-0.01160	0.00317	0.00067
caudate	0.10890	0.00923	0.00000	0.06607	0.00761	0.00000	-0.04287	0.00962	0.00009
bankssts	0.07732	0.00821	0.00000	0.04959	0.00677	0.00000	-0.02773	0.00856	0.00183
caudal anterior cingulate	0.08974	0.00841	0.00000	0.04248	0.00692	0.00000	-0.04726	0.00879	0.00001
caudal middle frontal	0.07033	0.00802	0.00000	0.04051	0.00660	0.00000	-0.02982	0.00838	0.00077
cuneus	0.02944	0.00555	0.00000	0.01900	0.00458	0.00005	-0.01043	0.00575	0.07244
entorhinal	0.01209	0.00325	0.00025	-0.00004	0.00266	0.98903	-0.01213	0.00343	0.00077
frontal pole	0.09010	0.00956	0.00000	0.05163	0.00783	0.00000	-0.03847	0.01007	0.00038
fusiform	0.04063	0.00486	0.00000	0.02217	0.00401	0.00000	-0.01845	0.00507	0.00067
inferior parietal	0.06262	0.00705	0.00000	0.03935	0.00580	0.00000	-0.02327	0.00737	0.00231
inferior temporal	0.04935	0.00581	0.00000	0.02968	0.00479	0.00000	-0.01968	0.00605	0.00181
insula	0.04494	0.00531	0.00000	0.02147	0.00436	0.00000	-0.02347	0.00557	0.00014
isthmus cingulate	0.06189	0.00742	0.00000	0.03463	0.00613	0.00000	-0.02727	0.00771	0.00077
lateral occipital	0.03961	0.00572	0.00000	0.02564	0.00471	0.00000	-0.01397	0.00596	0.02126
lateral orbitofrontal	0.06827	0.00716	0.00000	0.03701	0.00589	0.00000	-0.03125	0.00750	0.00016
lingual	0.02954	0.00486	0.00000	0.02136	0.00401	0.00000	-0.00818	0.00508	0.10847
medial orbitofrontal	0.08711	0.00920	0.00000	0.04842	0.00757	0.00000	-0.03869	0.00962	0.00021
middle temporal	0.04827	0.00570	0.00000	0.02818	0.00470	0.00000	-0.02009	0.00595	0.00126
paracentral	0.05924	0.00709	0.00000	0.03854	0.00583	0.00000	-0.02070	0.00741	0.00649
parahippocampal	0.02606	0.00381	0.00000	0.00851	0.00313	0.00765	-0.01755	0.00400	0.00010
pars opercularis	0.06887	0.00718	0.00000	0.03614	0.00588	0.00000	-0.03273	0.00755	0.00011
pars orbitalis	0.05829	0.00686	0.00000	0.03632	0.00566	0.00000	-0.02197	0.00715	0.00277
pars triangularis	0.06583	0.00733	0.00000	0.04006	0.00601	0.00000	-0.02576	0.00770	0.00137
pericalcarine	0.08906	0.01276	0.00000	0.06067	0.01051	0.00000	-0.02839	0.01331	0.03549
postcentral	0.04367	0.00558	0.00000	0.02928	0.00461	0.00000	-0.01439	0.00581	0.01577
posterior cingulate	0.08852	0.00855	0.00000	0.04613	0.00705	0.00000	-0.04239	0.00893	0.00004
precentral	0.04043	0.00516	0.00000	0.02382	0.00425	0.00000	-0.01661	0.00539	0.00277
precuneus	0.10490	0.00963	0.00000	0.06558	0.00794	0.00000	-0.03928	0.01004	0.00029
rostral anterior cingulate	0.09611	0.00890	0.00000	0.04869	0.00732	0.00000	-0.04742	0.00931	0.00001
rostral middle frontal	0.09661	0.00929	0.00000	0.05681	0.00764	0.00000	-0.03980	0.00973	0.00017
superior frontal	0.08514	0.00844	0.00000	0.04551	0.00694	0.00000	-0.03963	0.00884	0.00009
superior parietal	0.07146	0.00754	0.00000	0.04674	0.00622	0.00000	-0.02472	0.00787	0.00235
superior temporal	0.05098	0.00583	0.00000	0.02899	0.00480	0.00000	-0.02199	0.00610	0.00069
supramarginal	0.06353	0.00698	0.00000	0.03830	0.00575	0.00000	-0.02523	0.00729	0.00098
temporal pole	0.02622	0.00388	0.00000	0.00949	0.00319	0.00365	-0.01673	0.00407	0.00017
transverse temporal	0.05666	0.00815	0.00000	0.03662	0.00671	0.00000	-0.02004	0.00852	0.02126
hippocampus	0.00703	0.00205	0.00071	-0.00078	0.00168	0.65979	-0.00781	0.00217	0.00069
pallidum	0.02983	0.00525	0.00000	0.00746	0.00433	0.09005	-0.02237	0.00547	0.00017
putamen	0.08778	0.00785	0.00000	0.05602	0.00647	0.00000	-0.03177	0.00819	0.00032
thalamus proper	0.03758	0.00482	0.00000	0.01628	0.00398	0.00006	-0.02130	0.00503	0.00014

Annotations: SE – Standard error; ROI = Region of interest; PSMD= peak width of skeletonized mean diffusivity; EYO= estimated years to symptom onset; CI= confidence interval; NC= non-carrier.

Cell colors: Yellow highlights for significant p-values, green highlights for the largest cortical and subcortical regional effects in each comparison (pre vs nc, post vs nc, and pre vs post).

Table S3a – LME Estimates of mean difference SUVR per EYO range in all 40 regions for Pre-200 MC versus NC

PiB SUVR Region	EYO category																															
	1: < -25				2: -25 <= EYO < -20				3: -20 <= EYO < -15				4: -15 <= EYO < -10				5: -10 <= EYO < -5				6: -5 <= EYO < 0				7: 0 <= EYO < 5				8: 5 <= EYO			
	Estimates	SE	*P* ROI-adjusted	*P* ROI & EYO category-adjusted	Estimates	SE	*P* ROI-adjusted	*P* ROI & EYO category-adjusted	Estimates	SE	*P* ROI-adjusted	*P* ROI & EYO category-adjusted	Estimates	SE	*P* ROI-adjusted	*P* ROI & EYO category-adjusted	Estimates	SE	*P* ROI-adjusted	*P* ROI & EYO category-adjusted	Estimates	SE	*P* ROI-adjusted	*P* ROI & EYO category-adjusted	Estimates	SE	*P* ROI-adjusted	*P* ROI & EYO category-adjusted	Estimates	SE	*P* ROI-adjusted	*P* ROI & EYO category-adjusted
amygdala	-0.11	0.11	1.00	0.51	-0.01	0.13	1.00	1.00	0.07	0.08	0.99	0.65	0.06	0.10	0.57	0.83	0.08	0.09	0.33	0.54	0.20	0.11	0.07	0.12	0.37	0.08	0.00	0.00	0.63	0.13	0.00	0.00
caudate	-0.05	0.34	1.00	1.00	0.02	0.39	1.00	1.00	0.16	0.26	0.99	0.81	0.84	0.30	0.10	0.01	1.87	0.26	0.00	0.00	2.50	0.33	0.00	0.00	2.79	0.25	0.00	0.00	3.93	0.37	0.00	0.00
bankssts	-0.02	0.31	1.00	1.00	0.01	0.36	1.00	1.00	-0.01	0.23	0.99	1.00	0.57	0.27	0.10	0.07	0.95	0.23	0.00	0.00	1.88	0.30	0.00	0.00	2.05	0.23	0.00	0.00	2.50	0.33	0.00	0.00
caudal anterior cingulate	-0.17	0.31	1.00	0.89	0.03	0.36	1.00	1.00	0.01	0.23	0.99	1.00	0.61	0.28	0.10	0.06	1.36	0.24	0.00	0.00	2.15	0.31	0.00	0.00	2.25	0.23	0.00	0.00	2.87	0.34	0.00	0.00
caudal middle frontal	-0.15	0.30	1.00	0.92	-0.03	0.35	1.00	1.00	0.03	0.23	0.99	1.00	0.57	0.27	0.10	0.07	0.94	0.23	0.00	0.00	1.85	0.30	0.00	0.00	1.92	0.23	0.00	0.00	1.99	0.33	0.00	0.00
cuneus	-0.01	0.21	1.00	1.00	0.10	0.24	1.00	0.98	0.03	0.16	0.99	1.00	0.44	0.19	0.10	0.04	0.49	0.16	0.00	0.01	0.76	0.20	0.00	0.00	0.97	0.15	0.00	0.00	0.76	0.22	0.00	0.00
entorhinal	-0.01	0.12	1.00	1.00	-0.01	0.14	1.00	1.00	0.02	0.09	0.99	1.00	0.06	0.11	0.59	0.87	0.20	0.09	0.03	0.06	0.14	0.12	0.24	0.40	0.19	0.09	0.03	0.07	0.63	0.13	0.00	0.00
frontal pole	0.07	0.35	1.00	1.00	0.00	0.40	1.00	1.00	-0.05	0.26	0.99	1.00	0.74	0.31	0.10	0.04	1.31	0.26	0.00	0.00	2.72	0.35	0.00	0.00	2.29	0.26	0.00	0.00	2.90	0.39	0.00	0.00
fusiform	-0.03	0.18	1.00	1.00	-0.01	0.21	1.00	1.00	0.00	0.14	0.99	1.00	0.29	0.16	0.12	0.13	0.48	0.14	0.00	0.00	0.93	0.18	0.00	0.00	1.09	0.14	0.00	0.00	1.21	0.20	0.00	0.00
inferior parietal	-0.14	0.26	1.00	0.89	0.01	0.31	1.00	1.00	0.05	0.20	0.99	1.00	0.39	0.24	0.15	0.17	0.85	0.20	0.00	0.00	1.49	0.26	0.00	0.00	1.67	0.20	0.00	0.00	1.94	0.29	0.00	0.00
inferior temporal	-0.06	0.22	1.00	1.00	0.02	0.25	1.00	1.00	0.01	0.17	0.99	1.00	0.30	0.19	0.17	0.21	0.63	0.17	0.00	0.00	1.07	0.22	0.00	0.00	1.29	0.16	0.00	0.00	1.52	0.24	0.00	0.00
insula	0.01	0.20	1.00	1.00	0.00	0.23	1.00	1.00	-0.04	0.15	0.99	1.00	0.26	0.18	0.18	0.23	0.59	0.15	0.00	0.00	1.04	0.20	0.00	0.00	1.14	0.15	0.00	0.00	1.51	0.22	0.00	0.00
isthmus cingulate	0.01	0.28	1.00	1.00	0.10	0.32	1.00	1.00	-0.01	0.21	0.99	1.00	0.28	0.25	0.30	0.44	0.95	0.21	0.00	0.00	1.33	0.27	0.00	0.00	1.60	0.21	0.00	0.00	2.09	0.30	0.00	0.00
lateral occipital	-0.05	0.21	1.00	1.00	-0.04	0.25	1.00	1.00	0.09	0.16	0.99	0.88	0.37	0.19	0.10	0.09	0.53	0.16	0.00	0.00	0.83	0.21	0.00	0.00	1.18	0.16	0.00	0.00	1.14	0.23	0.00	0.00
lateral orbitofrontal	-0.03	0.26	1.00	1.00	0.05	0.31	1.00	1.00	0.00	0.20	0.99	1.00	0.48	0.23	0.10	0.08	0.87	0.20	0.00	0.00	1.70	0.26	0.00	0.00	1.72	0.20	0.00	0.00	2.28	0.29	0.00	0.00
lingual	0.00	0.18	1.00	1.00	0.13	0.21	1.00	0.83	0.09	0.13	0.99	0.81	0.39	0.16	0.10	0.03	0.37	0.14	0.01	0.01	0.80	0.18	0.00	0.00	1.01	0.13	0.00	0.00	0.74	0.20	0.00	0.00
medial orbitofrontal	0.00	0.34	1.00	1.00	0.02	0.39	1.00	1.00	0.02	0.25	0.99	1.00	0.66	0.30	0.10	0.06	1.26	0.26	0.00	0.00	2.24	0.34	0.00	0.00	2.24	0.25	0.00	0.00	2.87	0.38	0.00	0.00
middle temporal	-0.11	0.21	1.00	0.89	-0.01	0.25	1.00	1.00	0.00	0.16	0.99	1.00	0.27	0.19	0.20	0.27	0.60	0.16	0.00	0.00	1.04	0.21	0.00	0.00	1.22	0.16	0.00	0.00	1.49	0.23	0.00	0.00
paracentral	-0.01	0.27	1.00	1.00	0.05	0.31	1.00	1.00	0.04	0.20	0.99	1.00	0.67	0.24	0.10	0.01	0.84	0.20	0.00	0.00	1.52	0.26	0.00	0.00	1.73	0.20	0.00	0.00	1.69	0.29	0.00	0.00
parahippocampal	0.01	0.14	1.00	1.00	0.03	0.16	1.00	1.00	-0.08	0.11	0.99	0.69	0.24	0.13	0.11	0.11	0.36	0.11	0.00	0.00	0.49	0.14	0.00	0.00	0.63	0.11	0.00	0.00	0.94	0.16	0.00	0.00
pars opercularis	-0.06	0.27	1.00	1.00	-0.05	0.31	1.00	1.00	-0.02	0.20	0.99	1.00	0.48	0.24	0.10	0.08	0.91	0.20	0.00	0.00	1.74	0.27	0.00	0.00	1.75	0.20	0.00	0.00	2.20	0.30	0.00	0.00
pars orbitalis	-0.03	0.26	1.00	1.00	0.00	0.30	1.00	1.00	0.03	0.19	0.99	1.00	0.33	0.23	0.19	0.25	0.80	0.20	0.00	0.00	1.41	0.25	0.00	0.00	1.45	0.19	0.00	0.00	1.87	0.28	0.00	0.00
pars triangularis	-0.07	0.27	1.00	1.00	-0.05	0.32	1.00	1.00	-0.01	0.20	0.99	1.00	0.36	0.24	0.19	0.24	0.87	0.21	0.00	0.00	1.85	0.27	0.00	0.00	1.68	0.21	0.00	0.00	1.97	0.30	0.00	0.00
pericalcarine	0.03	0.47	1.00	1.00	0.07	0.54	1.00	1.00	0.12	0.35	0.99	1.00	0.82	0.42	0.10	0.09	1.17	0.36	0.00	0.00	3.27	0.46	0.00	0.00	2.76	0.35	0.00	0.00	2.15	0.51	0.00	0.00
postcentral	-0.08	0.21	1.00	1.00	0.02	0.25	1.00	1.00	-0.02	0.16	0.99	1.00	0.39	0.19	0.10	0.08	0.60	0.16	0.00	0.00	1.18	0.21	0.00	0.00	1.15	0.16	0.00	0.00	1.24	0.23	0.00	0.00
posterior cingulate	-0.11	0.32	1.00	1.00	0.09	0.37	1.00	1.00	-0.05	0.24	0.99	1.00	0.51	0.28	0.12	0.13	1.39	0.24	0.00	0.00	2.09	0.31	0.00	0.00	2.25	0.24	0.00	0.00	2.79	0.35	0.00	0.00
precentral	-0.13	0.20	1.00	0.81	0.06	0.23	1.00	1.00	0.02	0.15	0.99	1.00	0.43	0.17	0.10	0.03	0.55	0.15	0.00	0.00	1.04	0.19	0.00	0.00	1.10	0.15	0.00	0.00	1.14	0.21	0.00	0.00
precuneus	-0.12	0.36	1.00	1.00	0.05	0.42	1.00	1.00	0.04	0.27	0.99	1.00	0.68	0.32	0.10	0.07	1.54	0.27	0.00	0.00	2.46	0.36	0.00	0.00	2.79	0.27	0.00	0.00	3.26	0.39	0.00	0.00
rostral anterior cingulate	0.03	0.32	1.00	1.00	0.06	0.38	1.00	1.00	-0.01	0.24	0.99	1.00	0.64	0.29	0.10	0.05	1.39	0.25	0.00	0.00	2.24	0.32	0.00	0.00	2.54	0.24	0.00	0.00	3.18	0.36	0.00	0.00
rostral middle frontal	-0.12	0.35	1.00	1.00	-0.06	0.40	1.00	1.00	0.04	0.26	0.99	1.00	0.74	0.31	0.10	0.03	1.44	0.26	0.00	0.00	2.52	0.34	0.00	0.00	2.54	0.26	0.00	0.00	2.90	0.38	0.00	0.00
superior frontal	-0.13	0.31	1.00	0.98	0.01	0.37	1.00	1.00	0.03	0.24	0.99	1.00	0.63	0.28	0.10	0.05	1.23	0.24	0.00	0.00	2.23	0.31	0.00	0.00	2.31	0.24	0.00	0.00	2.47	0.35	0.00	0.00
superior parietal	-0.16	0.28	1.00	0.87	0.04	0.33	1.00	1.00	0.09	0.21	0.99	0.98	0.49	0.25	0.10	0.10	1.06	0.22	0.00	0.00	1.89	0.28	0.00	0.00	1.91	0.21	0.00	0.00	2.09	0.31	0.00	0.00
superior temporal	-0.10	0.22	1.00	0.96	0.00	0.25	1.00	1.00	0.01	0.16	0.99	1.00	0.33	0.19	0.14	0.15	0.74	0.17	0.00	0.00	1.37	0.22	0.00	0.00	1.30	0.16	0.00	0.00	1.48	0.24	0.00	0.00
supramarginal	-0.08	0.26	1.00	1.00	-0.05	0.30	1.00	1.00	-0.03	0.20	0.99	1.00	0.37	0.23	0.17	0.20	0.86	0.20	0.00	0.00	1.57	0.26	0.00	0.00	1.64	0.20	0.00	0.00	1.90	0.29	0.00	0.00
temporal pole	-0.07	0.14	1.00	0.92	0.06	0.17	1.00	1.00	-0.01	0.11	0.99	1.00	0.14	0.13	0.33	0.48	0.30	0.11	0.01	0.01	0.54	0.14	0.00	0.00	0.59	0.11	0.00	0.00	0.92	0.16	0.00	0.00
transverse temporal	-0.03	0.30	1.00	1.00	0.01	0.35	1.00	1.00	-0.08	0.23	0.99	1.00	0.26	0.27	0.37	0.56	0.65	0.23	0.01	0.01	1.67	0.30	0.00	0.00	1.36	0.23	0.00	0.00	1.77	0.33	0.00	0.00
hippocampus	0.02	0.07	1.00	1.00	0.00	0.09	1.00	1.00	-0.01	0.06	0.99	1.00	0.09	0.07	0.20	0.28	0.08	0.06	0.17	0.27	0.12	0.07	0.11	0.19	0.14	0.06	0.02	0.03	0.44	0.08	0.00	0.00
pallidum	0.01	0.19	1.00	1.00	-0.14	0.22	1.00	0.83	0.05	0.15	0.99	1.00	0.08	0.17	0.65	0.95	0.29	0.15	0.05	0.09	0.78	0.19	0.00	0.00	0.59	0.14	0.00	0.00	1.30	0.21	0.00	0.00
putamen	-0.01	0.29	1.00	1.00	0.01	0.33	1.00	1.00	0.17	0.22	0.99	0.68	0.49	0.25	0.10	0.10	1.24	0.22	0.00	0.00	1.82	0.28	0.00	0.00	2.23	0.21	0.00	0.00	3.25	0.31	0.00	0.00
thalamus proper	-0.12	0.18	1.00	0.81	0.17	0.21	1.00	0.65	0.13	0.13	0.99	0.56	0.20	0.16	0.24	0.33	0.69	0.14	0.00	0.00	0.73	0.18	0.00	0.00	0.92	0.13	0.00	0.00	1.51	0.19	0.00	0.00

Annotations: SE – Standard error; ROI = Region of interest; PSMD= peak width of skeletonized mean diffusivity; EYO= estimated years to symptom onset; CI= confidence interval. P-value <0.05 are highlighted in yellow.

Table S3b – LME Estimates of mean difference SUVR per EYO range in all 40 regions for Post-200 MC versus NC

PiB SUVR Region	EYO category																															
	1: < -25				2: -25 <= EYO < -20				3: -20 <= EYO < -15				4: -15 <= EYO < -10				5: -10 <= EYO < -5				6: -5 <= EYO < 0				7: 0 <= EYO < 5				8: 5 <= EYO			
	Estimates	SE	*P* ROI-adjusted	*P* ROI & EYO category-adjusted	Estimates	SE	*P* ROI-adjusted	*P* ROI & EYO category-adjusted	Estimates	SE	*P* ROI-adjusted	*P* ROI & EYO category-adjusted	Estimates	SE	*P* ROI-adjusted	*P* ROI & EYO category-adjusted	Estimates	SE	*P* ROI-adjusted	*P* ROI & EYO category-adjusted	Estimates	SE	*P* ROI-adjusted	*P* ROI & EYO category-adjusted	Estimates	SE	*P* ROI-adjusted	*P* ROI & EYO category-adjusted	Estimates	SE	*P* ROI-adjusted	*P* ROI & EYO category-adjusted
amygdala	-0.06	0.10	0.99	0.68	-0.01	0.08	1.00	0.96	0.07	0.07	0.33	0.43	0.04	0.08	0.70	0.79	0.06	0.08	0.50	0.63	0.00	0.07	0.98	0.99	0.10	0.08	0.25	0.32	0.13	0.10	0.19	0.26
caudate	-0.03	0.29	0.99	0.95	0.03	0.25	1.00	0.95	0.25	0.20	0.26	0.31	0.92	0.25	0.01	0.00	1.22	0.25	0.00	0.00	1.48	0.22	0.00	0.00	1.74	0.25	0.00	0.00	2.16	0.29	0.00	0.00
bankssts	0.09	0.26	0.99	0.83	0.23	0.23	0.88	0.44	0.28	0.18	0.18	0.19	0.54	0.23	0.05	0.04	0.71	0.22	0.00	0.00	1.09	0.20	0.00	0.00	1.31	0.23	0.00	0.00	1.84	0.27	0.00	0.00
caudal anterior cingulate	-0.01	0.27	0.99	0.99	0.27	0.23	0.88	0.35	0.36	0.18	0.16	0.09	0.79	0.23	0.01	0.00	1.06	0.23	0.00	0.00	1.15	0.20	0.00	0.00	1.14	0.23	0.00	0.00	1.52	0.27	0.00	0.00
caudal middle frontal	0.02	0.26	0.99	0.96	0.22	0.23	0.88	0.45	0.32	0.18	0.17	0.12	0.37	0.23	0.13	0.16	0.51	0.22	0.02	0.04	0.94	0.20	0.00	0.00	1.14	0.22	0.00	0.00	1.45	0.26	0.00	0.00
cuneus	0.10	0.18	0.99	0.73	0.30	0.16	0.73	0.10	0.47	0.12	0.00	0.00	0.58	0.16	0.01	0.00	0.66	0.15	0.00	0.00	0.63	0.14	0.00	0.00	0.84	0.16	0.00	0.00	0.79	0.18	0.00	0.00
entorhinal	-0.04	0.10	0.99	0.83	-0.02	0.09	1.00	0.90	0.03	0.07	0.70	0.82	0.01	0.09	0.90	0.94	0.06	0.09	0.50	0.61	0.01	0.08	0.94	0.95	-0.11	0.09	0.26	0.34	0.03	0.11	0.79	0.86
frontal pole	0.19	0.30	0.99	0.66	0.00	0.26	1.00	1.00	0.35	0.21	0.17	0.15	0.55	0.26	0.07	0.06	1.01	0.25	0.00	0.00	1.27	0.22	0.00	0.00	1.25	0.26	0.00	0.00	1.93	0.31	0.00	0.00
fusiform	0.06	0.16	0.99	0.83	0.15	0.14	0.88	0.37	0.20	0.11	0.17	0.12	0.27	0.14	0.08	0.09	0.36	0.13	0.01	0.01	0.52	0.12	0.00	0.00	0.63	0.14	0.00	0.00	0.85	0.16	0.00	0.00
inferior parietal	0.09	0.23	0.99	0.82	0.20	0.20	0.88	0.42	0.37	0.16	0.10	0.04	0.41	0.20	0.07	0.07	0.52	0.19	0.01	0.02	0.92	0.17	0.00	0.00	1.16	0.20	0.00	0.00	1.48	0.23	0.00	0.00
inferior temporal	0.04	0.19	0.99	0.89	0.13	0.16	0.89	0.56	0.20	0.13	0.18	0.19	0.28	0.16	0.12	0.15	0.43	0.16	0.01	0.02	0.60	0.14	0.00	0.00	0.77	0.16	0.00	0.00	1.15	0.19	0.00	0.00
insula	0.04	0.17	0.99	0.90	0.03	0.15	1.00	0.89	0.16	0.12	0.23	0.27	0.28	0.15	0.09	0.11	0.38	0.14	0.01	0.02	0.50	0.13	0.00	0.00	0.56	0.15	0.00	0.00	0.73	0.17	0.00	0.00
isthmus cingulate	0.04	0.24	0.99	0.92	0.07	0.21	0.96	0.83	0.29	0.16	0.17	0.12	0.42	0.21	0.08	0.08	0.68	0.20	0.00	0.00	0.78	0.18	0.00	0.00	0.85	0.21	0.00	0.00	1.34	0.24	0.00	0.00
lateral occipital	0.10	0.18	0.99	0.71	0.29	0.16	0.73	0.11	0.45	0.13	0.00	0.00	0.42	0.16	0.03	0.02	0.48	0.15	0.00	0.01	0.66	0.14	0.00	0.00	0.89	0.16	0.00	0.00	1.14	0.19	0.00	0.00
lateral orbitofrontal	0.09	0.23	0.99	0.82	0.12	0.20	0.90	0.68	0.28	0.16	0.17	0.12	0.36	0.20	0.09	0.11	0.67	0.19	0.00	0.00	0.90	0.17	0.00	0.00	0.94	0.20	0.00	0.00	1.36	0.23	0.00	0.00
lingual	0.05	0.15	0.99	0.84	0.27	0.13	0.73	**0.08**	0.39	0.11	0.00	0.00	0.45	0.13	0.01	0.00	0.43	0.13	0.00	0.00	0.56	0.12	0.00	0.00	0.74	0.13	0.00	0.00	0.96	0.16	0.00	0.00
medial orbitofrontal	0.05	0.29	0.99	0.91	0.11	0.25	0.91	0.79	0.34	0.20	0.17	0.15	0.65	0.25	0.03	0.02	1.01	0.24	0.00	0.00	1.23	0.22	0.00	0.00	1.22	0.25	0.00	0.00	1.65	0.30	0.00	0.00
middle temporal	0.08	0.18	0.99	0.79	0.08	0.16	0.90	0.73	0.22	0.13	0.17	0.14	0.20	0.16	0.24	0.31	0.42	0.16	0.01	0.02	0.62	0.14	0.00	0.00	0.70	0.16	0.00	0.00	1.07	0.19	0.00	0.00
paracentral	0.08	0.23	0.99	0.83	0.36	0.20	0.73	0.12	0.42	0.16	0.06	0.02	0.58	0.20	0.02	0.01	0.71	0.19	0.00	0.00	1.02	0.17	0.00	0.00	1.20	0.20	0.00	0.00	1.48	0.23	0.00	0.00
parahippocampal	-0.03	0.12	0.99	0.90	0.02	0.11	1.00	0.92	0.14	0.08	0.18	0.17	0.20	0.11	0.09	0.10	0.20	0.10	0.05	0.08	0.18	0.09	0.05	0.08	0.20	0.11	0.06	0.10	0.33	0.12	0.01	0.02
pars opercularis	0.09	0.23	0.99	0.82	0.14	0.20	0.90	0.61	0.23	0.16	0.19	0.22	0.32	0.20	0.13	0.17	0.51	0.19	0.01	0.02	0.93	0.17	0.00	0.00	0.91	0.20	0.00	0.00	1.32	0.24	0.00	0.00
pars orbitalis	0.11	0.22	0.99	0.75	0.17	0.19	0.88	0.49	0.25	0.15	0.17	0.16	0.27	0.19	0.18	0.23	0.62	0.19	0.00	0.00	0.92	0.17	0.00	0.00	0.88	0.19	0.00	0.00	1.40	0.22	0.00	0.00
pars triangularis	0.08	0.23	0.99	0.83	0.13	0.20	0.90	0.67	0.25	0.16	0.18	0.20	0.34	0.20	0.12	0.15	0.62	0.20	0.00	0.01	0.99	0.18	0.00	0.00	0.97	0.20	0.00	0.00	1.47	0.24	0.00	0.00
pericalcarine	0.11	0.40	0.99	0.86	0.56	0.35	0.86	0.17	0.74	0.28	0.06	0.02	0.98	0.35	0.02	0.01	1.11	0.34	0.00	0.00	1.48	0.30	0.00	0.00	2.07	0.35	0.00	0.00	2.29	0.41	0.00	0.00
postcentral	0.04	0.18	0.99	0.90	0.11	0.16	0.90	0.64	0.23	0.13	0.17	0.12	0.35	0.16	0.06	0.05	0.40	0.15	0.01	0.02	0.64	0.14	0.00	0.00	0.79	0.16	0.00	0.00	1.08	0.18	0.00	0.00
posterior cingulate	0.05	0.27	0.99	0.92	0.28	0.24	0.88	0.34	0.39	0.19	0.16	0.07	0.68	0.24	0.02	0.01	0.98	0.23	0.00	0.00	1.15	0.21	0.00	0.00	1.21	0.24	0.00	0.00	1.80	0.28	0.00	0.00
precentral	0.05	0.17	0.99	0.86	0.20	0.15	0.88	0.26	0.25	0.12	0.14	0.06	0.34	0.15	0.05	0.04	0.34	0.14	0.02	0.03	0.56	0.13	0.00	0.00	0.68	0.15	0.00	0.00	0.96	0.17	0.00	0.00
precuneus	0.10	0.31	0.99	0.83	0.31	0.27	0.88	0.35	0.49	0.21	0.10	0.04	0.82	0.27	0.02	0.01	1.21	0.26	0.00	0.00	1.59	0.23	0.00	0.00	1.83	0.27	0.00	0.00	2.44	0.31	0.00	0.00
rostral anterior cingulate	0.02	0.28	0.99	0.96	0.21	0.24	0.88	0.52	0.34	0.19	0.17	0.13	0.67	0.24	0.02	0.01	1.11	0.24	0.00	0.00	1.39	0.21	0.00	0.00	1.27	0.24	0.00	0.00	1.61	0.28	0.00	0.00
rostral middle frontal	0.10	0.30	0.99	0.83	0.22	0.26	0.88	0.52	0.41	0.21	0.16	0.09	0.60	0.26	0.05	0.04	0.98	0.25	0.00	0.00	1.41	0.22	0.00	0.00	1.50	0.26	0.00	0.00	2.09	0.30	0.00	0.00
superior frontal	0.03	0.27	0.99	0.95	0.29	0.23	0.88	0.31	0.37	0.19	0.16	0.09	0.57	0.24	0.04	0.03	0.79	0.23	0.00	0.00	1.18	0.20	0.00	0.00	1.32	0.23	0.00	0.00	1.63	0.28	0.00	0.00
superior parietal	0.08	0.24	0.99	0.83	0.32	0.21	0.88	0.20	0.46	0.17	0.06	0.01	0.60	0.21	0.02	0.01	0.77	0.21	0.00	0.00	1.36	0.18	0.00	0.00	1.33	0.21	0.00	0.00	1.73	0.25	0.00	0.00
superior temporal	0.06	0.19	0.99	0.84	0.08	0.16	0.90	0.77	0.18	0.13	0.21	0.24	0.29	0.16	0.11	0.13	0.52	0.16	0.00	0.00	0.65	0.14	0.00	0.00	0.72	0.16	0.00	0.00	1.03	0.19	0.00	0.00
supramarginal	0.08	0.22	0.99	0.83	0.11	0.20	0.90	0.73	0.24	0.16	0.18	0.19	0.38	0.20	0.08	0.09	0.50	0.19	0.01	0.02	0.87	0.17	0.00	0.00	1.00	0.20	0.00	0.00	1.41	0.23	0.00	0.00
temporal pole	0.00	0.12	0.99	1.00	0.02	0.11	1.00	0.94	0.11	0.09	0.26	0.31	-0.04	0.11	0.75	0.83	0.18	0.10	0.09	0.13	0.25	0.09	0.01	0.02	0.12	0.11	0.28	0.37	0.37	0.13	0.00	0.01
transverse temporal	0.09	0.26	0.99	0.83	0.11	0.23	0.90	0.77	0.21	0.18	0.29	0.35	0.50	0.23	0.06	0.06	0.56	0.22	0.01	0.02	0.71	0.20	0.00	0.00	1.01	0.23	0.00	0.00	1.32	0.27	0.00	0.00
hippocampus	-0.03	0.06	0.99	0.82	-0.02	0.06	0.98	0.85	0.05	0.04	0.30	0.37	0.03	0.06	0.66	0.75	0.02	0.05	0.72	0.83	0.01	0.05	0.89	0.90	-0.05	0.06	0.38	0.51	-0.01	0.07	0.83	0.90
pallidum	0.10	0.17	0.99	0.70	-0.01	0.14	1.00	0.97	-0.01	0.11	0.92	0.95	0.10	0.14	0.53	0.61	0.20	0.14	0.17	0.23	0.28	0.13	0.03	0.06	0.13	0.14	0.38	0.49	0.19	0.17	0.26	0.35
putamen	-0.02	0.24	0.99	0.96	0.00	0.21	1.00	0.99	0.17	0.17	0.33	0.43	0.65	0.21	0.02	0.01	0.89	0.21	0.00	0.00	1.14	0.19	0.00	0.00	1.39	0.21	0.00	0.00	1.91	0.25	0.00	0.00
thalamus proper	-0.09	0.15	0.99	0.71	0.07	0.13	0.90	0.73	0.16	0.11	0.18	0.18	0.34	0.13	0.03	0.02	0.46	0.13	0.00	0.00	0.37	0.12	0.00	0.00	0.36	0.13	0.01	0.02	0.61	0.15	0.00	0.00

Annotations: SE – Standard error; ROI = Region of interest; PSMD= peak width of skeletonized mean diffusivity; EYO= estimated years to symptom onset; CI= confidence interval. P-value <0.05 are highlighted in yellow.

Table S3c – LME Estimates of mean difference SUVR per EYO range in all 40 regions for pre-200 MC versus post-200 MC

PiB SUVR Region	EYO category																															
	1: < -25				2: -25 <= EYO < -20				3: -20 <= EYO < -15				4: -15 <= EYO < -10				5: -10 <= EYO < -5				6: -5 <= EYO < 0				7: 0 <= EYO < 5				8: 5 <= EYO			
	Estimates	SE	*P* ROI-adjusted	*P* ROI & EYO category-adjusted	Estimates	SE	*P* ROI-adjusted	*P* ROI & EYO category-adjusted	Estimates	SE	*P* ROI-adjusted	*P* ROI & EYO category-adjusted	Estimates	SE	*P* ROI-adjusted	*P* ROI & EYO category-adjusted	Estimates	SE	*P* ROI-adjusted	*P* ROI & EYO category-adjusted	Estimates	SE	*P* ROI-adjusted	*P* ROI & EYO category-adjusted	Estimates	SE	*P* ROI-adjusted	*P* ROI & EYO category-adjusted	Estimates	SE	*P* ROI-adjusted	*P* ROI & EYO category-adjusted
amygdala	0.05	0.12	0.99	0.87	0.00	0.14	0.99	0.99	-0.003	0.092	1.00	0.99	-0.024	0.106	0.97	0.93	-0.027	0.090	0.78	0.91	-0.20	0.11	0.08	0.21	-0.27	0.08	0.00	0.00	-0.498	0.12	0.00	0.00
caudate	0.02	0.37	0.99	0.99	0.01	0.41	0.99	0.99	0.084	0.273	0.84	0.91	0.079	0.318	0.97	0.93	-0.652	0.266	0.43	**0.06**	-1.02	0.33	0.01	0.01	-1.05	0.22	0.00	0.00	-1.766	0.36	0.00	0.00
bankssts	0.11	0.34	0.99	0.90	0.22	0.37	0.99	0.82	0.285	0.248	0.35	0.50	-0.025	0.289	0.97	0.99	-0.242	0.242	0.44	0.59	-0.79	0.30	0.02	0.04	-0.75	0.20	0.00	0.00	-0.657	0.32	0.08	0.15
caudal anterior cingulate	0.16	0.34	0.99	0.86	0.24	0.38	0.99	0.81	0.349	0.253	0.35	0.41	0.178	0.294	0.97	0.82	-0.298	0.247	0.43	0.48	-1.00	0.31	0.01	0.01	-1.11	0.21	0.00	0.00	-1.359	0.33	0.00	0.00
caudal middle frontal	0.17	0.33	0.99	0.85	0.25	0.36	0.99	0.78	0.285	0.245	0.35	0.50	-0.202	0.285	0.97	0.77	-0.429	0.239	0.43	0.22	-0.91	0.30	0.01	0.01	-0.78	0.20	0.00	0.00	-0.540	0.32	0.13	0.26
cuneus	0.11	0.23	0.99	0.86	0.20	0.25	0.99	0.71	0.443	0.168	0.35	0.04	0.137	0.195	0.97	0.77	0.179	0.162	0.43	0.53	-0.14	0.20	0.49	0.78	-0.13	0.14	0.35	0.62	0.029	0.22	0.92	0.97
entorhinal	-0.03	0.13	0.99	0.93	-0.01	0.15	0.99	0.99	0.013	0.098	0.94	0.97	-0.049	0.114	0.97	0.87	-0.135	0.096	0.43	0.40	-0.13	0.12	0.29	0.53	-0.30	0.08	0.00	0.00	-0.598	0.13	0.00	0.00
frontal pole	0.13	0.39	0.99	0.90	0.01	0.43	0.99	0.99	0.402	0.285	0.35	0.40	-0.186	0.331	0.97	0.82	-0.304	0.280	0.43	0.53	-1.45	0.35	0.00	0.00	-1.03	0.24	0.00	0.00	-0.977	0.38	0.02	0.04
fusiform	0.09	0.20	0.99	0.87	0.16	0.22	0.99	0.76	0.198	0.148	0.35	0.43	-0.022	0.172	0.97	0.97	-0.112	0.144	0.53	0.72	-0.41	0.18	0.03	0.08	-0.46	0.12	0.00	0.00	-0.362	0.19	0.10	0.19
inferior parietal	0.23	0.29	0.99	0.72	0.19	0.32	0.99	0.82	0.317	0.215	0.35	0.36	0.018	0.250	0.97	0.99	-0.332	0.210	0.43	0.30	-0.58	0.26	0.04	0.10	-0.51	0.18	0.00	0.02	-0.457	0.28	0.14	0.28
inferior temporal	0.10	0.24	0.99	0.87	0.11	0.26	0.99	0.87	0.189	0.177	0.38	0.54	-0.025	0.206	0.97	0.97	-0.199	0.172	0.43	0.50	-0.47	0.21	0.04	0.11	-0.52	0.14	0.00	0.00	-0.372	0.23	0.14	0.28
insula	0.02	0.22	0.99	0.98	0.03	0.24	0.99	0.97	0.197	0.160	0.35	0.47	0.011	0.186	0.97	0.99	-0.216	0.157	0.43	0.41	-0.53	0.19	0.02	0.03	-0.58	0.13	0.00	0.00	-0.775	0.21	0.00	0.00
isthmus cingulate	0.03	0.30	0.99	0.97	-0.03	0.33	0.99	0.99	0.302	0.223	0.35	0.42	0.143	0.260	0.97	0.82	-0.273	0.217	0.43	0.45	-0.56	0.27	0.05	0.14	-0.74	0.18	0.00	0.00	-0.747	0.29	0.02	0.05
lateral occipital	0.15	0.23	0.99	0.81	0.33	0.26	0.99	0.44	0.367	0.172	0.35	0.12	0.047	0.200	0.97	0.93	-0.051	0.168	0.78	0.91	-0.17	0.21	0.42	0.70	-0.28	0.14	0.05	0.15	-0.003	0.22	0.99	0.99
lateral orbitofrontal	0.12	0.29	0.99	0.87	0.08	0.32	0.99	0.93	0.282	0.215	0.35	0.43	-0.116	0.249	0.97	0.86	-0.201	0.210	0.44	0.61	-0.81	0.26	0.01	0.01	-0.78	0.18	0.00	0.00	-0.919	0.28	0.00	0.01
lingual	0.05	0.20	0.99	0.92	0.14	0.22	0.99	0.81	0.301	0.146	0.35	0.14	0.058	0.169	0.97	0.90	0.060	0.143	0.75	0.87	-0.23	0.18	0.20	0.43	-0.27	0.12	0.03	0.10	0.214	0.19	0.31	0.52
medial orbitofrontal	0.05	0.37	0.99	0.97	0.09	0.41	0.99	0.93	0.322	0.276	0.35	0.50	-0.014	0.320	0.97	0.99	-0.257	0.270	0.44	0.61	-1.01	0.33	0.01	0.01	-1.01	0.23	0.00	0.00	-1.221	0.36	0.00	0.01
middle temporal	0.19	0.24	0.99	0.70	0.10	0.26	0.99	0.88	0.213	0.173	0.35	0.47	-0.066	0.202	0.97	0.90	-0.185	0.169	0.43	0.53	-0.42	0.21	0.06	0.16	-0.52	0.14	0.00	0.00	-0.418	0.23	0.10	0.21
paracentral	0.09	0.29	0.99	0.91	0.31	0.32	0.99	0.60	0.386	0.216	0.35	0.23	-0.086	0.251	0.97	0.90	-0.132	0.211	0.62	0.82	-0.50	0.26	0.06	0.18	-0.53	0.18	0.00	0.02	-0.204	0.28	0.51	0.76
parahippocampal	-0.04	0.16	0.99	0.93	-0.01	0.17	0.99	0.99	0.218	0.115	0.35	0.19	-0.037	0.134	0.97	0.92	-0.151	0.113	0.43	0.43	-0.31	0.14	0.04	0.10	-0.43	0.10	0.00	0.00	-0.609	0.15	0.00	0.00
pars opercularis	0.15	0.30	0.99	0.85	0.20	0.33	0.99	0.82	0.254	0.219	0.35	0.50	-0.161	0.254	0.97	0.81	-0.391	0.215	0.43	0.21	-0.81	0.27	0.01	0.01	-0.84	0.18	0.00	0.00	-0.884	0.29	0.01	0.01
pars orbitalis	0.14	0.28	0.99	0.85	0.17	0.31	0.99	0.82	0.219	0.207	0.38	0.55	-0.057	0.241	0.97	0.93	-0.183	0.202	0.46	0.65	-0.49	0.25	0.06	0.17	-0.57	0.17	0.00	0.01	-0.471	0.27	0.12	0.24
pars triangularis	0.15	0.30	0.99	0.86	0.18	0.33	0.99	0.83	0.254	0.223	0.35	0.51	-0.015	0.259	0.97	0.99	-0.258	0.219	0.43	0.50	-0.85	0.27	0.01	0.01	-0.71	0.18	0.00	0.00	-0.496	0.29	0.13	0.26
pericalcarine	0.08	0.51	0.99	0.97	0.49	0.56	0.99	0.66	0.611	0.379	0.35	0.28	0.153	0.440	0.97	0.90	-0.067	0.370	0.86	0.95	-1.80	0.46	0.00	0.00	-0.69	0.31	0.03	0.10	0.146	0.49	0.81	0.91
postcentral	0.12	0.23	0.99	0.85	0.08	0.25	0.99	0.90	0.243	0.171	0.35	0.39	-0.036	0.199	0.97	0.95	-0.196	0.166	0.43	0.50	-0.54	0.21	0.02	0.04	-0.36	0.14	0.01	0.04	-0.161	0.22	0.51	0.76
posterior cingulate	0.15	0.35	0.99	0.87	0.19	0.38	0.99	0.86	0.434	0.258	0.35	0.26	0.169	0.299	0.97	0.82	-0.412	0.252	0.43	0.28	-0.94	0.31	0.01	0.01	-1.04	0.21	0.00	0.00	-0.985	0.34	0.01	0.02
precentral	0.17	0.21	0.99	0.71	0.13	0.23	0.99	0.82	0.229	0.158	0.35	0.38	-0.088	0.184	0.97	0.86	-0.205	0.154	0.43	0.43	-0.48	0.19	0.02	0.06	-0.42	0.13	0.00	0.01	-0.178	0.21	0.44	0.68
precuneus	0.22	0.39	0.99	0.82	0.27	0.43	0.99	0.82	0.451	0.291	0.35	0.31	0.137	0.338	0.97	0.87	-0.327	0.283	0.43	0.50	-0.87	0.35	0.02	0.06	-0.96	0.24	0.00	0.00	-0.817	0.38	0.06	0.12
rostral anterior cingulate	-0.01	0.36	0.99	0.99	0.14	0.39	0.99	0.89	0.350	0.264	0.35	0.43	0.033	0.307	0.97	0.98	-0.280	0.259	0.43	0.53	-0.85	0.32	0.02	0.04	-1.27	0.22	0.00	0.00	-1.565	0.35	0.00	0.00
rostral middle frontal	0.22	0.38	0.99	0.82	0.27	0.42	0.99	0.81	0.362	0.282	0.35	0.44	-0.147	0.327	0.97	0.87	-0.460	0.276	0.43	0.27	-1.11	0.34	0.01	0.01	-1.03	0.23	0.00	0.00	-0.810	0.37	0.06	0.11
superior frontal	0.16	0.35	0.99	0.87	0.29	0.38	0.99	0.74	0.337	0.256	0.35	0.43	-0.061	0.297	0.97	0.94	-0.433	0.250	0.43	0.24	-1.05	0.31	0.01	0.01	-0.99	0.21	0.00	0.00	-0.841	0.33	0.03	0.05
superior parietal	0.24	0.31	0.99	0.73	0.28	0.34	0.99	0.70	0.375	0.229	0.35	0.28	0.109	0.266	0.97	0.87	-0.291	0.223	0.43	0.43	-0.54	0.28	0.06	0.17	-0.58	0.19	0.00	0.01	-0.356	0.30	0.28	0.49
superior temporal	0.16	0.24	0.99	0.81	0.08	0.26	0.99	0.91	0.173	0.178	0.40	0.60	-0.048	0.207	0.97	0.93	-0.218	0.174	0.43	0.45	-0.72	0.22	0.01	0.01	-0.58	0.15	0.00	0.00	-0.448	0.23	0.09	0.18
supramarginal	0.16	0.29	0.99	0.82	0.16	0.32	0.99	0.85	0.275	0.213	0.35	0.44	0.014	0.247	0.97	0.99	-0.361	0.208	0.43	0.24	-0.70	0.26	0.02	0.04	-0.64	0.17	0.00	0.00	-0.495	0.28	0.12	0.23
temporal pole	0.07	0.16	0.99	0.87	-0.05	0.17	0.99	0.91	0.120	0.117	0.38	0.57	-0.172	0.136	0.97	0.45	-0.113	0.115	0.44	0.60	-0.29	0.14	0.05	0.14	-0.47	0.10	0.00	0.00	-0.549	0.15	0.00	0.00
transverse temporal	0.12	0.33	0.99	0.89	0.10	0.37	0.99	0.92	0.287	0.247	0.35	0.50	0.243	0.287	0.97	0.69	-0.090	0.241	0.77	0.89	-0.96	0.30	0.01	0.01	-0.35	0.20	0.08	0.24	-0.446	0.32	0.21	0.41
hippocampus	-0.05	0.08	0.99	0.82	-0.02	0.09	0.99	0.94	0.057	0.061	0.41	0.62	-0.063	0.071	0.97	0.65	-0.060	0.060	0.44	0.59	-0.11	0.07	0.15	0.35	-0.19	0.05	0.00	0.00	-0.452	0.08	0.00	0.00
pallidum	0.09	0.21	0.99	0.87	0.13	0.23	0.99	0.82	-0.062	0.157	0.79	0.87	0.026	0.182	0.97	0.97	-0.093	0.152	0.62	0.82	-0.51	0.19	0.02	0.04	-0.46	0.13	0.00	0.00	-1.106	0.20	0.00	0.00
putamen	0.00	0.31	0.99	0.99	-0.01	0.34	0.99	0.99	0.000	0.231	1.00	1.00	0.156	0.269	0.97	0.82	-0.354	0.225	0.43	0.31	-0.69	0.28	0.03	0.06	-0.85	0.19	0.00	0.00	-1.330	0.30	0.00	0.00
thalamus proper	0.03	0.19	0.99	0.97	-0.10	0.21	0.99	0.86	0.038	0.144	0.86	0.92	0.142	0.167	0.97	0.69	-0.232	0.140	0.43	0.27	-0.36	0.17	0.05	0.14	-0.56	0.12	0.00	0.00	-0.901	0.19	0.00	0.00

Annotations: SE – Standard error; ROI = Region of interest; PSMD= peak width of skeletonized mean diffusivity; EYO= estimated years to symptom onset; CI= confidence interval. P-value <0.05 are highlighted in yellow.

Table S4 – LME results mutation by EYO effect on SVD measures (Global PSMD, total, PV, Ant, and Post WMH volumes)

SVD variables	Region	Pre-200 slope vs. NC slope			Post-200 slope vs. NC slope			Pre-200 slope vs. Post-200 slope		
		**Estimate**	**SE**	***P* unadjusted (*P* ROI-adjusted*)**	**Estimate**	**SE**	***P* unadjusted (*P* ROI-adjusted*)**	**Estimate**	**SE**	***P* unadjusted (*P* ROI-adjusted*)**
WMH Volumes	anterior periventricular*	-1.70	15.10	0.91 (0.91*)	21.11	13.76	0.13 (0.13*)	22.81	15.67	0.15 (0.15*)
	posterior periventricular*	10.61	19.31	0.58 (0.91*)	50.04	17.64	0.005 (0.02*)	39.43	20.14	**0.05 (0.08*)**
	total periventricular	-3.87	29.96	0.90 (0.91*)	61.46	27.40	0.03 (0.04*)	65.32	31.77	**0.04 (0.08*)**
	total WMH log (SPM method)	0.03	0.01	0.02	0.05	0.01	0.00	0.02	0.02	0.27
PSMD	Global measure	2.73E-03	2.55E-03	0.29	1.24E-02	2.17E-03	0.00	9.63E-03	2.67E-03	0.00

** adjusted for multiple comparison (n=3 regions)*

Annotations: SVD = small vessel disease; WMH= white matter hyperintensity; SPM = statistical parametric mapping; SE – Standard error; ROI = Region of interest; PSMD= peak width of skeletonized mean diffusivity; EYO= estimated years to symptom onset. P-value <0.05 are highlighted in yellow. In Bold, tests close to significance (P-value <.10).

Table S5a – LME Estimates of mean difference in **Global SVD measures** per EYO range for pre-200 MC versus NC, post-200 MC versus NC, and pre-200 versus post-200 MC

Global SVD measures	Test	EYO category											
		1: < -10				2: -10 <= EYO < 0				3: 0 <= EYO			
		Estimates	SE	*P* unadjusted	*P* EYO category-adjusted	Estimates	SE	*P* unadjusted	*P* EYO category-adjusted	Estimates	SE	*P* unadjusted	*P* EYO category-adjusted
PSMD	Pre-200 vs NC	0.05	0.04	0.26	0.39	-0.05	0.07	0.45	0.45	0.21	0.06	0.001	0.004
total WMHlog		-0.15	0.26	0.57	0.57	0.46	0.31	0.14	0.21	0.98	0.34	0.004	0.013
PSMD	Post-200 vs NC	-0.02	0.04	0.53	0.53	0.06	0.06	0.28	0.42	0.33	0.06	0.00	0.00
total WMHlog		-0.03	0.21	0.89	0.89	0.10	0.27	0.71	0.89	1.23	0.29	3.74E-05	1.12E-04
PSMD	Pre-200 vs Post-200	-0.07	0.05	0.14	0.14	0.12	0.08	0.14	0.14	0.12	0.06	0.03	**0.10**
total WMHlog		0.12	0.28	0.68	0.68	-0.36	0.33	0.28	0.61	0.25	0.30	0.41	0.61

Table S5b – LME Estimates of mean difference in **WMH Regions** per EYO range for pre-200 MC versus NC, post-200 MC versus NC, and pre-200 versus post-200 MC

WMH Regions (semi-automated methods)	Test	EYO category											
		1: < -10				2: -10 <= EYO < 0				3: 0 <= EYO			
		Estimates	SE	*P* unadjusted (*P* ROI-adjusted*)	*P* ROI & EYO category-adjusted	Estimates	SE	*P* unadjusted (*P* ROI-adjusted*)	*P* ROI & EYO category-adjusted	Estimates	SE	*P* unadjusted (*P* ROI-adjusted*)	*P* ROI & EYO category-adjusted
anterior periventricular	Pre-200 vs NC	23.0	266.9	0.93 (0.93*)	0.93	264.5	328.6	0.42 (0.76*)	0.86	-174.3	370.8	0.64 (0.75*)	0.86
posterior periventricular		159.7	342.1	0.64 (0.93*)	0.86	127.0	418.4	0.76 (0.76*)	0.86	657.2	464.8	0.16 (0.48*)	0.86
total periventricular		306.9	535.5	0.56 (0.93*)	0.86	430.6	663.8	0.52 (0.76*)	0.86	228.6	722.7	0.75 (0.75*)	0.86
anterior periventricular	Post-200 vs NC	-8.2	226.2	0.97 (0.97*)	0.97	381.1	281.3	0.18 (0.18*)	0.29	409.1	335.5	0.22 (0.22*)	0.29
posterior periventricular		-355.4	289.3	0.22 (0.66*)	0.29	970.0	361.3	**0.008 (0.02*)**	**0.07**	863.8	421.3	**0.04** (0.13*)	**0.13**
total periventricular		-291.9	453.5	0.52 (0.78*)	0.59	1365.7	569.9	**0.02 (0.03*)**	**0.08**	1022.5	652.9	0.11 (0.18*)	0.27
anterior periventricular	Pre-200 vs Post-200	-31.2	282.2	0.91 (0.91*)	0.91	116.6	352.9	0.74 (0.74*)	0.83	583.4	311.7	0.06 (0.19*)	0.29
posterior periventricular		-515.1	353.2	0.15 (0.44*)	0.37	843.0	445.5	**0.06 (0.18*)**	0.29	206.6	388.6	0.60 (0.60*)	0.77
total periventricular		-598.8	567.2	0.29 (0.44*)	0.44	935.1	710.9	0.19 (0.29*)	0.37	793.9	625.1	0.21 (0.31*)	0.37

Annotations in Tables S5a and S5b: SE – Standard error; ROI = Region of interest; PSMD= peak width of skeletonized mean diffusivity; EYO= estimated years to symptom onset; CI= confidence interval.

P-value <0.05 are highlighted in yellow. In Bold, tests close to significance (P-value <.10).

Table S6a – Results of Negative binomial mixed effect models evaluating the effect of mutation position on **Deep WMH volumes per EYO**

**WMH Region**	**Pre-200 slope vs. NC slope**			**Post-200 slope vs. NC slope**			**Pre-200 slope vs. Post-200 slope**		
	**Estimate**	**SE**	***P* unadjusted**	**Estimate**	**SE**	***P* unadjusted**	**Estimate**	**SE**	***P* unadjusted**
Deep WMH	-16.4	20.2	0.6955	19.1	18.8	0.5682	-35.5	20.2	0.1883

Table S6b – Results of Negative binomial mixed effect models evaluating the effect of mutation position on **Deep WMH volumes per EYO range**

**Deep WMH**	**EYO category**											
	**1: < -10**				**2: -10 <= EYO < 0**				**3: 0 <= EYO**			
	**Estimates**	**SE**	***P* unadjusted**	***P* EYO category-adjusted**	**Estimates**	**SE**	***P* unadjusted**	***P* EYO category-adjusted**	**Estimates**	**SE**	***P* unadjusted**	***P* EYO category-adjusted**
Pre-200 vs NC	275.7	434.0	0.80	0.93	341.3	443.0	0.72	0.93	-212.3	589.0	0.93	0.93
Post-200 vs NC	182.6	366.0	0.87	0.92	-143.1	380.0	0.92	0.92	515.4	502.0	0.56	0.92
Pre-200 vs Post-200	93.1	480.0	0.98	0.98	484.4	507.0	0.61	0.91	-727.7	419.0	0.20	0.59

Annotations in Tables S6a and S6b: EYO= estimated years to symptom onset; SE – Standard error; WMH = white matter hyperintensity.

Table S7 – Microhemorrhages prevalence, count, and location per mutation group

	NC (N=148)	MC pre-200 (N=83)	MC post-200 (N=162)	Overall (N=393)	P-value
**Total MCH presence**					
Not present	142 (95.9%)	74 (89.2%)	145 (89.5%)	361 (91.9%)	0.0857
Present	6 (4.1%)	5 (6.0%)	17 (10.5%)	28 (7.1%)	
**Frontal MCH presence**					
Not present	146 (98.6%)	78 (94.0%)	154 (95.1%)	378 (96.2%)	0.1053
Present	2 (1.4%)	1 (1.2%)	8 (4.9%)	11 (2.8%)	
**Parietal MCH presence**					
Not present	147 (99.3%)	76 (91.6%)	157 (96.9%)	380 (96.7%)	0.2283
Present	1 (0.7%)	3 (3.6%)	5 (3.1%)	9 (2.3%)	
**Temporal MCH presence**					
Not present	146 (98.6%)	76 (91.6%)	153 (94.4%)	375 (95.4%)	0.1387
Present	2 (1.4%)	3 (3.6%)	9 (5.6%)	14 (3.6%)	
**Occipital MCH presence**					
Not present	147 (99.3%)	76 (91.6%)	154 (95.1%)	377 (95.9%)	0.0876
Present	1 (0.7%)	3 (3.6%)	8 (4.9%)	12 (3.1%)	
**Cerebellar MCH presence**					
Not present	148 (100%)	79 (95.2%)	161 (99.4%)	388 (98.7%)	0.4954
Present	0 (0%)	0 (0%)	1 (0.6%)	1 (0.3%)	
**Deep MCH presence**					
Not present	148 (100%)	77 (92.8%)	158 (97.5%)	383 (97.5%)	0.1538
Present	0 (0%)	2 (2.4%)	4 (2.5%)	6 (1.5%)	
**Total MCH count**					
Median (IQR)	0.0 (0.0)	0.0 (0.0)	0.0 (0.0)	0.0 (0.0)	0.0761
[Min, Max]	[0.0, 1.0]	[0.0, 40.0]	[0.0, 135.0]	[0.0, 135.0]	
**Frontal MCH count**					
Median (IQR)	0.0 (0.0)	0.0 (0.0)	0.0 (0.0)	0.0 (0.0)	0.106
[Min, Max]	[0.0, 1.0]	[0.0, 6.0]	[0.0, 23.0]	[0.0, 23.0]	
**Parietal MCH count**					
Median (IQR)	0.0 (0.0)	0.0 (0.0)	0.0 (0.0)	0.0 (0.0)	0.2275
[Min, Max]	[0.0, 1.0]	[0.0, 5.0]	[0.0, 34.0]	[0.0, 34.0]	
**Temporal MCH count**					
Median (IQR)	0.0 (0.0)	0.0 (0.0)	0.0 (0.0)	0.0 (0.0)	0.1359
[Min, Max]	[0.0, 1.0]	[0.0, 7.0]	[0.0, 25.0]	[0.0, 25.0]	
**Occipital MCH count**					
Median (IQR)	0.0 (0.0)	0.0 (0.0)	0.0 (0.0)	0.0 (0.0)	0.0868
[Min, Max]	[0.0, 1.0]	[0.0, 21.0]	[0.0, 49.0]	[0.0, 49.0]	

Table S8 – Mutation position effect conditional of EYO on microhemorrhage count per region

Region	EYO (exact break point)	NC vs. pre200	Unadjusted *P*-value	Adjusted *P*-value	NC vs. post200	Unadjusted *P*-value	Adjusted *P*-value	pre200 vs. post200	Unadjusted *P*-value	Adjusted *P*-value
Total	-10	39.732 (71.363)	0.1019	0.5095	0.237 (0.213)	0.2451	0.4893	**0.006** (0.009)**	**0.0027**	**0.0162**
	-5	5.014 (6.402)	0.4172	0.6953	0.233 (0.183)	0.1536	0.4893	**0.047** (0.043)**	**0.0027**	**0.0162**
	0.5	0.514 (0.465)	0.7425	0.9281	0.229 (0.188)	0.1739	0.4893	**0.445 (0.195)**	**0.1544**	0.6176
Frontal	-10	>1000 (-)			0.164 (0.28)	0.5397	0.7196	0 (0)	0.8917	0.9728
	-5	>1000 (-)			0.28 (0.328)	0.5238	0.7196	0 (0)	0.8892	0.9728
	0.5	>1000 (-)			0.502 (0.522)	0.7850	0.8061	0 (0)	0.8782	0.9728
Parietal	-10									
	-5									
	0.5									
Temporal	-10	>20 (-)			0.289 (0.408)	0.6536	0.7843	0.009 (0.064)	0.7757	0.9728
	-5	5.002 (-)			0.192 (0.209)	0.2854	0.4893	0.038 (0.179)	0.7640	0.9728
	0.5	0.667 (1.592)	0.9842	0.9842	**0.122 (0.115)**	**0.0663**	0.3978	0.184 (0.418)	0.7368	0.9728
Occipital	-10	>1000 (-)			0.418 (0.583)	0.8061	0.8061	0 (0)	0.2434	0.7302
	-5	72.789 (-)			0.098 (0.141)	0.2393	0.4893	0.001 (0.007)	0.3660	0.8784
	0.5	0.018 (0.046)	0.2490	0.6225	**0.02 (0.034)**	**0.0544**	0.3978	1.1 (1.852)	0.9982	0.9982

Significant pairwise comparisons are shaded in yellow, and trends are indicated in bold. Comparisons without enough power or with too large effects are shaded in grey.
Annotations: EYO= estimated years to symptom onset; CI= confidence interval.
*P-value thresholds based on unadjusted comparison: * <0.05; ** <0.005.*

Table S9 – LME Estimates of mean difference in **Clinical measures** per EYO range for pre-200 MC versus NC, post-200 MC versus NC, and pre-200 versus post-200 MC

Clinical and Cognitive Measures	Test	EYO category																															
		1: < -25				2: -25 <= EYO < -20				3: -20 <= EYO < -15				4: -15 <= EYO < -10				5: -10 <= EYO < -5				6: -5 <= EYO < 0				7: 0 <= EYO < 5				8: 5 <= EYO			
		Estimates	SE	*P* unadjusted	*P* EYO category-adjusted	Estimates	SE	*P* unadjusted	*P* EYO category-adjusted	Estimates	SE	*P* unadjusted	*P* EYO category-adjusted	Estimates	SE	*P* unadjusted	*P* EYO category-adjusted	Estimates	SE	*P* unadjusted	*P* EYO category-adjusted	Estimates	SE	*P* unadjusted	*P* EYO category-adjusted	Estimates	SE	*P* unadjusted	*P* EYO category-adjusted	Estimates	SE	*P* unadjusted	*P* EYO category-adjusted
CDR-SB	Pre-200 vs NC	0.00	0.70	1.00	1.00	-0.08	0.85	0.93	1.00	0.00	0.54	0.99	1.00	-0.05	0.62	0.93	1.00	0.00	0.54	0.99	1.00	0.29	0.64	0.65	1.00	2.67	0.54	1.13E-06	4.54E-06	3.81	0.75	6.07E-07	4.54E-06
MMSE		-0.31	1.36	0.82	0.97	0.31	1.65	0.85	0.97	-0.01	1.04	0.99	0.99	-0.63	1.20	0.60	0.97	0.46	1.04	0.66	0.97	-1.14	1.26	0.37	0.97	-5.60	1.05	1.75E-07	1.40E-06	-5.67	1.47	1.42E-04	5.70E-04
CogComp		-0.03	0.22	0.88	0.88	0.22	0.28	0.43	0.69	0.03	0.17	0.87	0.88	0.21	0.20	0.30	0.59	0.06	0.17	0.71	0.88	-0.38	0.21	0.07	0.19	-1.22	0.18	6.01E-11	4.81E-10	-1.58	0.25	1.16E-09	4.62E-09
CDR-SB	Post-200 vs NC	0.00	0.61	0.99	1.00	0.00	0.53	1.00	1.00	0.13	0.43	0.77	1.00	-0.07	0.52	0.89	1.00	0.11	0.49	0.82	1.00	0.17	0.47	0.72	1.00	2.72	0.50	8.86E-08	3.55E-07	9.31	0.60	0.00E+00	0.00E+00
MMSE		0.19	1.20	0.87	0.98	0.18	1.02	0.86	0.98	-0.02	0.85	0.98	0.98	-0.10	1.01	0.92	0.98	-0.30	0.94	0.75	0.98	-0.54	0.91	0.55	0.98	-4.77	0.97	1.29E-06	5.16E-06	-15.01	1.19	0.00E+00	0.00E+00
CogComp		-0.08	0.20	0.69	0.73	0.06	0.17	0.73	0.73	-0.19	0.14	0.18	0.29	0.12	0.17	0.47	0.62	-0.28	0.15	0.07	0.15	-0.46	0.15	2.36E-03	6.30E-03	-1.16	0.16	9.17E-12	3.67E-11	-2.08	0.22	2.00E-18	1.60E-17
CDR-SB	Post-200 vs Pre-200	0.00	0.75	1.00	1.00	0.08	0.86	0.93	1.00	0.13	0.57	0.82	1.00	-0.02	0.64	0.98	1.00	0.11	0.54	0.84	1.00	-0.12	0.64	0.85	1.00	0.05	0.42	0.90	1.00	5.50	0.70	3.49E-14	2.79E-13
MMSE		0.50	1.45	0.73	0.97	-0.13	1.68	0.94	0.99	-0.01	1.12	0.99	0.99	0.53	1.25	0.67	0.97	-0.77	1.08	0.48	0.97	0.60	1.25	0.63	0.97	0.83	0.82	0.31	0.97	-9.35	1.37	3.75E-11	3.00E-10
CogComp		-0.05	0.24	0.85	0.85	-0.16	0.28	0.57	0.80	-0.22	0.19	0.24	0.64	-0.09	0.21	0.68	0.80	**-0.34**	**0.18**	**0.06**	0.26	-0.08	0.21	0.70	0.80	0.06	0.15	0.66	0.80	**-0.50**	**0.25**	**0.05**	0.26

Significant pairwise comparisons are shaded in yellow, and trends are indicated in bold.

Annotations: EYO= estimated years to symptom onset; SE= standard error; CDR-SB= clinical dementia rating sum of boxes; MMSE= mini-mental state examination; CogComp= cognitive composite.

*P-value thresholds based on unadjusted comparison: * <0.05; ** <0.005.*

Table S10 – Mediation Analyses conditional per EYO with individual PiB regions as mediator

PiB Variable (mediator)	Effect	EYO=-15			EYO=-10			EYO=-5			EYO=0			EYO=0.5			EYO=1		
		Estimates (95% CI)	*P* value	Adjusted p value	Estimates (95% CI)	*P* value	Adjusted p value	Estimates (95% CI)	*P* value	Adjusted p value	Estimates (95% CI)	*P* value	Adjusted p value	Estimates (95% CI)	*P* value	Adjusted p value	Estimates (95% CI)	*P* value	Adjusted p value
amygdala	ACME	-0.04(-0.13, 0.03)	0.2660	0.6945	-0.08*(-0.18, -0.01)	0.0220	0.1022	-0.12**(-0.22, -0.03)	0.0000	0.0000	-0.16**(-0.29, -0.06)	0.0000	0.0000	-0.16**(-0.3, -0.05)	0.0000	0.0000	-0.17**(-0.31, -0.07)	0.0000	0.0000
	ADE	0.02(-0.21, 0.29)	0.8400	0.9880	0.14(-0.09, 0.36)	0.2300	0.4436	0.25*(0.03, 0.49)	0.0300	0.0941	0.38*(0.09, 0.69)	0.0120	0.0383	0.4**(0.12, 0.69)	0.0060	0.0282	0.42**(0.12, 0.73)	0.0060	0.0267
	TE	-0.02(-0.27, 0.25)	0.8960	0.9980	0.06(-0.18, 0.29)	0.6160	0.6503	0.14(-0.09, 0.37)	0.2760	0.2840	0.23(-0.05, 0.52)	0.1300	0.1660	0.24(-0.06, 0.54)	0.1040	0.1497	0.25(-0.04, 0.56)	0.1160	0.1600
caudate	ACME	-0.05`(-0.12, 0)	0.0800	0.6945	-0.07`(-0.15, 0)	0.0580	0.1078	-0.09*(-0.2, -0.01)	0.0400	0.0552	-0.11`(-0.24, 0)	0.0540	0.0617	-0.11*(-0.25, 0)	0.0460	0.0558	-0.11`(-0.25, 0.01)	0.0660	0.0776
	ADE	0.02(-0.25, 0.29)	0.8820	0.9880	0.14(-0.08, 0.38)	0.2700	0.4436	0.23`(-0.02, 0.49)	0.0720	0.1076	0.33*(0.03, 0.67)	0.0260	0.0385	0.35*(0.05, 0.69)	0.0320	0.0424	0.36*(0.02, 0.67)	0.0300	0.0414
	TE	-0.03(-0.29, 0.24)	0.8600	0.9980	0.07(-0.14, 0.31)	0.5660	0.6503	0.15(-0.1, 0.38)	0.2400	0.2840	0.23(-0.06, 0.54)	0.1120	0.1660	0.24`(-0.05, 0.56)	0.0780	0.1497	0.25(-0.05, 0.54)	0.1180	0.1600
bankssts	ACME	-0.04(-0.13, 0.04)	0.3680	0.6945	-0.07*(-0.16, 0)	0.0440	0.1022	-0.11**(-0.22, -0.03)	0.0040	0.0094	-0.15**(-0.28, -0.04)	0.0020	0.0042	-0.16**(-0.28, -0.05)	0.0000	0.0000	-0.16**(-0.29, -0.05)	0.0040	0.0064
	ADE	0.02(-0.25, 0.28)	0.8580	0.9880	0.15(-0.09, 0.37)	0.2080	0.4436	0.26*(0.02, 0.51)	0.0300	0.0941	0.38*(0.09, 0.67)	0.0140	0.0383	0.4*(0.1, 0.7)	0.0100	0.0282	0.41**(0.1, 0.69)	0.0060	0.0267
	TE	-0.02(-0.3, 0.25)	0.9480	0.9980	0.07(-0.19, 0.31)	0.5440	0.6503	0.15(-0.09, 0.41)	0.2300	0.2840	0.24(-0.07, 0.55)	0.1240	0.1660	0.24(-0.07, 0.56)	0.1140	0.1497	0.25(-0.06, 0.57)	0.1340	0.1600
caudal anterior cingulate	ACME	-0.02(-0.07, 0.02)	0.4420	0.7168	-0.04`(-0.11, 0)	0.0720	0.1171	-0.07`(-0.17, 0.01)	0.0800	0.0960	-0.1`(-0.22, 0.01)	0.0980	0.1059	-0.1`(-0.22, 0)	0.0540	0.0635	-0.1`(-0.22, 0.02)	0.0880	0.0951
	ADE	0(-0.27, 0.29)	0.9560	0.9880	0.11(-0.12, 0.34)	0.3940	0.4635	0.22`(-0.05, 0.48)	0.0980	0.1153	0.33`(-0.02, 0.66)	0.0660	0.0695	0.34*(0.01, 0.68)	0.0340	0.0424	0.35*(0.03, 0.69)	0.0360	0.0436
	TE	-0.01(-0.29, 0.27)	0.9300	0.9980	0.06(-0.17, 0.31)	0.6220	0.6503	0.15(-0.1, 0.4)	0.2080	0.2840	0.24(-0.08, 0.54)	0.1400	0.1660	0.24(-0.06, 0.58)	0.1380	0.1497	0.25(-0.07, 0.56)	0.1080	0.1600
caudal middle frontal	ACME	-0.04(-0.12, 0.03)	0.2660	0.6945	-0.07*(-0.16, -0.01)	0.0280	0.1022	-0.11**(-0.21, -0.03)	0.0020	0.0053	-0.14**(-0.26, -0.04)	0.0000	0.0000	-0.14**(-0.27, -0.05)	0.0000	0.0000	-0.14**(-0.27, -0.05)	0.0000	0.0000
	ADE	0.02(-0.23, 0.3)	0.8820	0.9880	0.14(-0.09, 0.38)	0.2160	0.4436	0.26*(0.01, 0.51)	0.0440	0.0978	0.38*(0.07, 0.67)	0.0220	0.0383	0.4*(0.09, 0.69)	0.0100	0.0282	0.39*(0.08, 0.7)	0.0180	0.0348
	TE	-0.01(-0.28, 0.27)	0.9060	0.9980	0.07(-0.17, 0.32)	0.5220	0.6503	0.15(-0.11, 0.4)	0.2260	0.2840	0.24(-0.07, 0.53)	0.1220	0.1660	0.25(-0.04, 0.55)	0.1100	0.1497	0.25(-0.07, 0.56)	0.1240	0.1600
cuneus	ACME	0.03(-0.02, 0.09)	0.2640	0.6945	0.02(-0.02, 0.07)	0.4100	0.4316	0(-0.04, 0.05)	0.9040	0.9040	-0.01(-0.07, 0.04)	0.7320	0.7320	-0.01(-0.06, 0.04)	0.7620	0.7620	-0.01(-0.07, 0.05)	0.7760	0.7760
	ADE	-0.04(-0.3, 0.23)	0.7860	0.9880	0.05(-0.19, 0.3)	0.6800	0.6800	0.15(-0.12, 0.4)	0.2520	0.2520	0.24(-0.08, 0.56)	0.1440	0.1440	0.25(-0.06, 0.55)	0.1040	0.1040	0.26`(-0.03, 0.56)	0.0860	0.0860
	TE	-0.01(-0.28, 0.26)	0.9220	0.9980	0.06(-0.17, 0.31)	0.5620	0.6503	0.15(-0.12, 0.41)	0.2640	0.2840	0.23(-0.08, 0.55)	0.1520	0.1660	0.24(-0.07, 0.53)	0.1300	0.1497	0.25(-0.04, 0.56)	0.1120	0.1600
entorhinal	ACME	-0.03(-0.1, 0.01)	0.1940	0.6945	-0.05*(-0.13, 0)	0.0440	0.1022	-0.08*(-0.17, -0.01)	0.0320	0.0474	-0.1*(-0.22, -0.01)	0.0180	0.0248	-0.1*(-0.21, 0)	0.0460	0.0558	-0.1*(-0.21, -0.01)	0.0240	0.0320
	ADE	0.01(-0.24, 0.28)	0.9140	0.9880	0.12(-0.1, 0.35)	0.3420	0.4436	0.22`(-0.02, 0.46)	0.0760	0.1076	0.32*(0.04, 0.6)	0.0280	0.0386	0.34*(0.03, 0.66)	0.0300	0.0424	0.35*(0.01, 0.63)	0.0380	0.0447
	TE	-0.02(-0.29, 0.25)	0.8900	0.9980	0.06(-0.16, 0.29)	0.6280	0.6503	0.14(-0.09, 0.38)	0.2460	0.2840	0.22(-0.07, 0.5)	0.1280	0.1660	0.24(-0.06, 0.53)	0.1080	0.1497	0.25(-0.07, 0.53)	0.1220	0.1600
frontal pole	ACME	-0.03(-0.11, 0.03)	0.3340	0.6945	-0.07*(-0.15, 0)	0.0400	0.1022	-0.1**(-0.2, -0.02)	0.0000	0.0000	-0.13**(-0.26, -0.04)	0.0040	0.0070	-0.14**(-0.27, -0.04)	0.0020	0.0042	-0.14**(-0.27, -0.04)	0.0020	0.0044
	ADE	0.03(-0.23, 0.3)	0.8620	0.9880	0.15(-0.09, 0.42)	0.2300	0.4436	0.26*(0.02, 0.49)	0.0300	0.0941	0.36*(0.06, 0.66)	0.0220	0.0383	0.38*(0.05, 0.69)	0.0220	0.0367	0.39*(0.04, 0.68)	0.0260	0.0385
	TE	-0.01(-0.28, 0.28)	0.9440	0.9980	0.08(-0.16, 0.34)	0.5260	0.6503	0.16(-0.08, 0.4)	0.2200	0.2840	0.23(-0.08, 0.52)	0.1320	0.1660	0.25(-0.06, 0.54)	0.1240	0.1497	0.25(-0.05, 0.53)	0.1060	0.1600
fusiform	ACME	-0.03(-0.13, 0.07)	0.5640	0.7277	-0.08`(-0.17, 0)	0.0620	0.1078	-0.12**(-0.25, -0.03)	0.0060	0.0126	-0.17**(-0.29, -0.06)	0.0000	0.0000	-0.18**(-0.3, -0.06)	0.0020	0.0042	-0.18**(-0.33, -0.07)	0.0000	0.0000
	ADE	0.02(-0.23, 0.29)	0.8680	0.9880	0.15(-0.09, 0.39)	0.2180	0.4436	0.28*(0.02, 0.52)	0.0280	0.0941	0.4*(0.11, 0.69)	0.0120	0.0383	0.41**(0.09, 0.73)	0.0080	0.0282	0.43**(0.12, 0.76)	0.0060	0.0267
	TE	0(-0.28, 0.27)	0.9900	0.9980	0.07(-0.19, 0.32)	0.5580	0.6503	0.16(-0.11, 0.41)	0.2440	0.2840	0.23(-0.08, 0.53)	0.1460	0.1660	0.23(-0.07, 0.56)	0.1340	0.1497	0.25(-0.07, 0.6)	0.1240	0.1600
inferior parietal	ACME	-0.01(-0.1, 0.07)	0.7120	0.7911	-0.04(-0.13, 0.03)	0.2080	0.2570	-0.08*(-0.18, 0)	0.0360	0.0514	-0.11**(-0.23, -0.03)	0.0080	0.0128	-0.11**(-0.23, -0.03)	0.0080	0.0119	-0.12**(-0.23, -0.03)	0.0020	0.0044
	ADE	0.01(-0.24, 0.26)	0.9760	0.9880	0.12(-0.11, 0.38)	0.3600	0.4436	0.24`(-0.02, 0.49)	0.0660	0.1076	0.35*(0.03, 0.63)	0.0360	0.0447	0.36*(0.04, 0.67)	0.0300	0.0424	0.37*(0.04, 0.69)	0.0260	0.0385
	TE	-0.01(-0.27, 0.26)	0.9820	0.9980	0.07(-0.18, 0.32)	0.5480	0.6503	0.16(-0.1, 0.43)	0.2460	0.2840	0.23(-0.07, 0.52)	0.1440	0.1660	0.24(-0.07, 0.57)	0.1320	0.1497	0.26(-0.07, 0.58)	0.1480	0.1600
inferior temporal	ACME	-0.04(-0.14, 0.05)	0.4360	0.7168	-0.08`(-0.19, 0.01)	0.0780	0.1171	-0.13**(-0.24, -0.03)	0.0060	0.0126	-0.17**(-0.32, -0.06)	0.0000	0.0000	-0.18**(-0.32, -0.06)	0.0000	0.0000	-0.18**(-0.32, -0.06)	0.0000	0.0000
	ADE	0.03(-0.25, 0.3)	0.7980	0.9880	0.16(-0.07, 0.38)	0.1960	0.4436	0.28*(0.03, 0.52)	0.0280	0.0941	0.41*(0.12, 0.71)	0.0100	0.0383	0.41**(0.11, 0.71)	0.0080	0.0282	0.43**(0.14, 0.74)	0.0080	0.0320
	TE	-0.01(-0.3, 0.26)	0.9900	0.9980	0.07(-0.18, 0.33)	0.5500	0.6503	0.15(-0.11, 0.41)	0.2360	0.2840	0.24(-0.07, 0.55)	0.1240	0.1660	0.23(-0.09, 0.54)	0.1460	0.1497	0.25(-0.04, 0.55)	0.1160	0.1600
insula	ACME	-0.03(-0.12, 0.04)	0.3700	0.6945	-0.08*(-0.17, -0.01)	0.0340	0.1022	-0.13**(-0.25, -0.04)	0.0000	0.0000	-0.17**(-0.3, -0.06)	0.0000	0.0000	-0.18**(-0.31, -0.08)	0.0020	0.0042	-0.18**(-0.32, -0.07)	0.0000	0.0000
	ADE	0.03(-0.22, 0.3)	0.8080	0.9880	0.15(-0.09, 0.4)	0.2340	0.4436	0.29*(0.04, 0.51)	0.0240	0.0941	0.41*(0.11, 0.72)	0.0160	0.0383	0.42**(0.09, 0.73)	0.0040	0.0282	0.43**(0.1, 0.74)	0.0060	0.0267
	TE	0(-0.27, 0.28)	0.9940	0.9980	0.07(-0.19, 0.32)	0.5460	0.6503	0.16(-0.08, 0.4)	0.2080	0.2840	0.24(-0.07, 0.53)	0.1320	0.1660	0.25(-0.08, 0.55)	0.1320	0.1497	0.25(-0.07, 0.56)	0.1000	0.1600
isthmus cingulate	ACME	-0.03(-0.13, 0.05)	0.3540	0.6945	-0.07*(-0.16, 0)	0.0360	0.1022	-0.11**(-0.21, -0.03)	0.0000	0.0000	-0.15**(-0.27, -0.05)	0.0000	0.0000	-0.15**(-0.28, -0.06)	0.0040	0.0070	-0.16**(-0.31, -0.05)	0.0040	0.0064
	ADE	0.03(-0.24, 0.28)	0.8000	0.9880	0.15(-0.08, 0.4)	0.2160	0.4436	0.27*(0.03, 0.51)	0.0280	0.0941	0.39*(0.08, 0.67)	0.0100	0.0383	0.4*(0.09, 0.7)	0.0120	0.0282	0.42*(0.1, 0.72)	0.0120	0.0343
	TE	0(-0.28, 0.25)	0.9960	0.9980	0.08(-0.15, 0.34)	0.5480	0.6503	0.16(-0.08, 0.4)	0.1960	0.2840	0.23(-0.05, 0.54)	0.1360	0.1660	0.25(-0.06, 0.56)	0.1240	0.1497	0.26(-0.07, 0.55)	0.1240	0.1600
lateral occipital	ACME	0.02(-0.07, 0.11)	0.6520	0.7543	-0.01(-0.09, 0.07)	0.8360	0.8360	-0.04(-0.13, 0.05)	0.3840	0.4042	-0.06(-0.17, 0.03)	0.2140	0.2253	-0.07(-0.18, 0.03)	0.1660	0.1747	-0.07(-0.19, 0.03)	0.1880	0.1979
	ADE	-0.03(-0.3, 0.24)	0.8220	0.9880	0.08(-0.17, 0.33)	0.5260	0.5518	0.19(-0.06, 0.46)	0.1440	0.1516	0.31*(0.02, 0.62)	0.0420	0.0480	0.32*(0.02, 0.62)	0.0360	0.0424	0.33`(-0.01, 0.64)	0.0560	0.0589
	TE	-0.01(-0.28, 0.27)	0.9220	0.9980	0.07(-0.2, 0.34)	0.5700	0.6503	0.16(-0.12, 0.43)	0.2440	0.2840	0.25(-0.07, 0.57)	0.1120	0.1660	0.25(-0.06, 0.57)	0.1260	0.1497	0.26(-0.1, 0.6)	0.1360	0.1600
lateral orbitofrontal	ACME	-0.04(-0.14, 0.05)	0.3380	0.6945	-0.09*(-0.18, -0.01)	0.0200	0.1022	-0.14**(-0.26, -0.05)	0.0000	0.0000	-0.19**(-0.34, -0.08)	0.0000	0.0000	-0.2**(-0.35, -0.09)	0.0000	0.0000	-0.2**(-0.36, -0.08)	0.0020	0.0044
	ADE	0.04(-0.21, 0.3)	0.7460	0.9880	0.17(-0.06, 0.4)	0.1460	0.4436	0.3*(0.06, 0.55)	0.0100	0.0941	0.43**(0.12, 0.74)	0.0020	0.0383	0.44**(0.15, 0.73)	0.0020	0.0282	0.45**(0.14, 0.76)	0.0040	0.0267
	TE	0(-0.26, 0.27)	0.9860	0.9980	0.08(-0.16, 0.32)	0.5460	0.6503	0.15(-0.09, 0.43)	0.2060	0.2840	0.23(-0.08, 0.54)	0.1420	0.1660	0.24(-0.05, 0.53)	0.1220	0.1497	0.24(-0.08, 0.56)	0.1640	0.1660
lingual	ACME	0.01(-0.07, 0.1)	0.7500	0.8108	-0.01(-0.09, 0.07)	0.8260	0.8360	-0.03(-0.11, 0.05)	0.4360	0.4472	-0.05(-0.15, 0.04)	0.2420	0.2482	-0.05(-0.15, 0.04)	0.3300	0.3385	-0.05(-0.16, 0.05)	0.2420	0.2482
	ADE	-0.02(-0.31, 0.25)	0.8860	0.9880	0.08(-0.15, 0.31)	0.5380	0.5518	0.18(-0.07, 0.45)	0.1700	0.1744	0.27`(-0.04, 0.57)	0.0800	0.0821	0.29`(-0.01, 0.57)	0.0620	0.0636	0.3`(-0.02, 0.6)	0.0740	0.0759
	TE	-0.01(-0.3, 0.28)	0.9320	0.9980	0.07(-0.18, 0.32)	0.5820	0.6503	0.15(-0.12, 0.43)	0.2460	0.2840	0.22(-0.09, 0.51)	0.1660	0.1660	0.25(-0.07, 0.54)	0.1460	0.1497	0.24(-0.09, 0.57)	0.1660	0.1660
medial orbitofrontal	ACME	-0.03(-0.11, 0.02)	0.2360	0.6945	-0.07*(-0.14, -0.01)	0.0140	0.1022	-0.1**(-0.2, -0.03)	0.0020	0.0053	-0.13**(-0.25, -0.04)	0.0000	0.0000	-0.14**(-0.27, -0.03)	0.0020	0.0042	-0.14**(-0.27, -0.03)	0.0040	0.0064
	ADE	0.02(-0.24, 0.3)	0.8880	0.9880	0.14(-0.08, 0.36)	0.2420	0.4436	0.25`(-0.03, 0.5)	0.0760	0.1076	0.37*(0.05, 0.67)	0.0200	0.0383	0.38*(0.07, 0.7)	0.0120	0.0282	0.39*(0.06, 0.72)	0.0200	0.0348
	TE	-0.01(-0.28, 0.27)	0.9160	0.9980	0.07(-0.16, 0.29)	0.5320	0.6503	0.15(-0.12, 0.39)	0.2580	0.2840	0.24(-0.06, 0.54)	0.1140	0.1660	0.24(-0.06, 0.56)	0.1160	0.1497	0.25(-0.06, 0.57)	0.1260	0.1600
middle temporal	ACME	-0.02(-0.11, 0.05)	0.5600	0.7277	-0.07`(-0.16, 0.01)	0.0800	0.1171	-0.11*(-0.22, -0.02)	0.0120	0.0200	-0.15**(-0.27, -0.05)	0.0020	0.0042	-0.15**(-0.27, -0.05)	0.0000	0.0000	-0.16**(-0.3, -0.04)	0.0020	0.0044
	ADE	0.02(-0.24, 0.3)	0.8460	0.9880	0.14(-0.11, 0.37)	0.2640	0.4436	0.26*(0.01, 0.5)	0.0340	0.0941	0.38*(0.08, 0.7)	0.0180	0.0383	0.4*(0.1, 0.71)	0.0120	0.0282	0.42**(0.11, 0.72)	0.0040	0.0267
	TE	0(-0.28, 0.28)	0.9980	0.9980	0.07(-0.19, 0.31)	0.5880	0.6503	0.15(-0.1, 0.42)	0.2740	0.2840	0.23(-0.08, 0.56)	0.1420	0.1660	0.25(-0.06, 0.59)	0.1260	0.1497	0.27`(-0.04, 0.58)	0.0900	0.1600
paracentral	ACME	-0.01(-0.09, 0.06)	0.8100	0.8480	-0.03(-0.11, 0.03)	0.2660	0.3040	-0.06`(-0.14, 0.01)	0.0840	0.0960	-0.08*(-0.19, 0)	0.0420	0.0560	-0.08*(-0.2, -0.01)	0.0300	0.0414	-0.09*(-0.2, -0.01)	0.0240	0.0320
	ADE	-0.01(-0.27, 0.29)	0.9420	0.9880	0.1(-0.15, 0.33)	0.4740	0.5124	0.21`(-0.04, 0.45)	0.0980	0.1153	0.32*(0.01, 0.65)	0.0480	0.0533	0.33*(0.02, 0.62)	0.0400	0.0457	0.34`(0, 0.65)	0.0540	0.0584
	TE	-0.01(-0.29, 0.28)	0.8700	0.9980	0.06(-0.2, 0.31)	0.6600	0.6600	0.15(-0.11, 0.41)	0.2420	0.2840	0.24(-0.08, 0.57)	0.1560	0.1660	0.25(-0.07, 0.55)	0.1320	0.1497	0.25(-0.09, 0.56)	0.1520	0.1600
parahippocampal	ACME	-0.03(-0.1, 0.04)	0.3820	0.6945	-0.07*(-0.15, -0.01)	0.0220	0.1022	-0.11**(-0.23, -0.03)	0.0020	0.0053	-0.16**(-0.29, -0.05)	0.0040	0.0070	-0.16**(-0.29, -0.06)	0.0000	0.0000	-0.16**(-0.29, -0.05)	0.0000	0.0000
	ADE	0.02(-0.25, 0.29)	0.9100	0.9880	0.15(-0.09, 0.38)	0.2020	0.4436	0.26*(0.03, 0.53)	0.0320	0.0941	0.39*(0.09, 0.69)	0.0160	0.0383	0.4**(0.13, 0.71)	0.0080	0.0282	0.41*(0.1, 0.74)	0.0120	0.0343
	TE	-0.01(-0.29, 0.26)	0.9380	0.9980	0.08(-0.16, 0.32)	0.4960	0.6503	0.15(-0.09, 0.4)	0.2480	0.2840	0.23(-0.06, 0.53)	0.1200	0.1660	0.25`(-0.03, 0.55)	0.0780	0.1497	0.25(-0.06, 0.58)	0.1140	0.1600

PiB Variable (mediator)	Effect	EYO=-15			EYO=-10			EYO=-5			EYO=0			EYO=0.5			EYO=1		
		Estimates (95% CI)	*P* value	Adjusted p value	Estimates (95% CI)	*P* value	Adjusted p value	Estimates (95% CI)	*P* value	Adjusted p value	Estimates (95% CI)	*P* value	Adjusted p value	Estimates (95% CI)	*P* value	Adjusted p value	Estimates (95% CI)	*P* value	Adjusted p value
pars opercularis	ACME	-0.04(-0.13, 0.04)	0.2440	0.6945	-0.08*(-0.18, -0.01)	0.0160	0.1022	-0.13**(-0.25, -0.04)	0.0000	0.0000	-0.17**(-0.31, -0.07)	0.0000	0.0000	-0.18**(-0.33, -0.07)	0.0000	0.0000	-0.18**(-0.32, -0.06)	0.0060	0.0092
	ADE	0.04(-0.24, 0.3)	0.7880	0.9880	0.16(-0.07, 0.41)	0.1720	0.4436	0.29*(0.04, 0.54)	0.0260	0.0941	0.42**(0.12, 0.7)	0.0040	0.0383	0.42*(0.12, 0.74)	0.0100	0.0282	0.42*(0.1, 0.76)	0.0140	0.0348
	TE	0(-0.29, 0.28)	0.9740	0.9980	0.08(-0.16, 0.33)	0.5160	0.6503	0.16(-0.1, 0.4)	0.2260	0.2840	0.25(-0.06, 0.53)	0.1060	0.1660	0.24(-0.06, 0.55)	0.1080	0.1497	0.24(-0.07, 0.55)	0.1240	0.1600
pars orbitalis	ACME	-0.02(-0.11, 0.06)	0.6240	0.7543	-0.06(-0.15, 0.02)	0.1480	0.1910	-0.1*(-0.19, -0.02)	0.0120	0.0200	-0.13**(-0.25, -0.04)	0.0020	0.0042	-0.14**(-0.28, -0.04)	0.0080	0.0119	-0.14**(-0.26, -0.05)	0.0000	0.0000
	ADE	0.01(-0.24, 0.26)	0.9320	0.9880	0.14(-0.1, 0.37)	0.2160	0.4436	0.25`(-0.01, 0.48)	0.0560	0.1018	0.36*(0.07, 0.65)	0.0200	0.0383	0.37**(0.11, 0.66)	0.0060	0.0282	0.39*(0.11, 0.68)	0.0100	0.0343
	TE	-0.01(-0.27, 0.25)	0.9500	0.9980	0.09(-0.14, 0.33)	0.4460	0.6503	0.15(-0.1, 0.39)	0.2240	0.2840	0.23(-0.06, 0.54)	0.1300	0.1660	0.23(-0.04, 0.55)	0.1100	0.1497	0.24`(-0.05, 0.55)	0.0900	0.1600
pars triangularis	ACME	-0.03(-0.11, 0.05)	0.4480	0.7168	-0.06`(-0.15, 0.01)	0.0980	0.1307	-0.09*(-0.19, -0.01)	0.0160	0.0256	-0.13**(-0.26, -0.04)	0.0000	0.0000	-0.13**(-0.25, -0.03)	0.0020	0.0042	-0.13**(-0.26, -0.04)	0.0020	0.0044
	ADE	0.01(-0.25, 0.26)	0.9020	0.9880	0.14(-0.08, 0.36)	0.2420	0.4436	0.25*(0.01, 0.5)	0.0400	0.0941	0.37*(0.08, 0.66)	0.0100	0.0383	0.38**(0.08, 0.68)	0.0080	0.0282	0.39*(0.06, 0.69)	0.0260	0.0385
	TE	-0.01(-0.31, 0.25)	0.9400	0.9980	0.08(-0.14, 0.32)	0.5400	0.6503	0.16(-0.09, 0.42)	0.2380	0.2840	0.24(-0.05, 0.53)	0.1140	0.1660	0.25(-0.05, 0.53)	0.1080	0.1497	0.26(-0.07, 0.57)	0.1200	0.1600
pericalcarine	ACME	-0.01(-0.08, 0.04)	0.6600	0.7543	-0.03(-0.09, 0.02)	0.2980	0.3311	-0.04(-0.12, 0.01)	0.1320	0.1427	-0.06`(-0.14, 0.01)	0.0820	0.0911	-0.06`(-0.15, 0)	0.0700	0.0757	-0.06`(-0.15, 0)	0.0700	0.0800
	ADE	-0.01(-0.27, 0.25)	0.9620	0.9880	0.09(-0.15, 0.32)	0.4700	0.5124	0.19(-0.05, 0.42)	0.1260	0.1362	0.27`(-0.02, 0.59)	0.0620	0.0670	0.3`(-0.01, 0.58)	0.0540	0.0568	0.3`(0, 0.6)	0.0540	0.0584
	TE	-0.02(-0.3, 0.24)	0.8880	0.9980	0.06(-0.19, 0.3)	0.6340	0.6503	0.14(-0.1, 0.38)	0.2440	0.2840	0.22(-0.09, 0.54)	0.1620	0.1660	0.24(-0.08, 0.54)	0.1180	0.1497	0.24(-0.07, 0.55)	0.1080	0.1600
postcentral	ACME	-0.01(-0.09, 0.06)	0.6540	0.7543	-0.04(-0.12, 0.02)	0.2120	0.2570	-0.07*(-0.16, 0)	0.0480	0.0640	-0.09*(-0.19, -0.01)	0.0180	0.0248	-0.1**(-0.21, -0.02)	0.0060	0.0096	-0.1*(-0.21, -0.02)	0.0100	0.0148
	ADE	0.01(-0.26, 0.27)	0.9300	0.9880	0.11(-0.12, 0.36)	0.3440	0.4436	0.22`(-0.05, 0.46)	0.0920	0.1150	0.32*(0.02, 0.62)	0.0380	0.0447	0.33*(0.03, 0.64)	0.0320	0.0424	0.34*(0.02, 0.65)	0.0360	0.0436
	TE	0(-0.28, 0.28)	0.9720	0.9980	0.07(-0.17, 0.32)	0.5460	0.6503	0.15(-0.11, 0.4)	0.2540	0.2840	0.23(-0.08, 0.52)	0.1360	0.1660	0.23(-0.07, 0.55)	0.1160	0.1497	0.24(-0.06, 0.56)	0.1360	0.1600
posterior cingulate	ACME	-0.02(-0.1, 0.04)	0.4920	0.7277	-0.06*(-0.15, 0)	0.0460	0.1022	-0.11**(-0.22, -0.03)	0.0000	0.0000	-0.15**(-0.29, -0.05)	0.0040	0.0070	-0.15**(-0.29, -0.05)	0.0060	0.0096	-0.16**(-0.3, -0.05)	0.0000	0.0000
	ADE	0.01(-0.25, 0.26)	0.9120	0.9880	0.14(-0.1, 0.37)	0.2100	0.4436	0.25`(0, 0.51)	0.0520	0.1018	0.39*(0.09, 0.7)	0.0120	0.0383	0.4*(0.08, 0.74)	0.0200	0.0348	0.4*(0.08, 0.72)	0.0180	0.0348
	TE	-0.01(-0.28, 0.27)	0.9740	0.9980	0.08(-0.15, 0.33)	0.5040	0.6503	0.15(-0.11, 0.39)	0.2840	0.2840	0.24(-0.05, 0.56)	0.1040	0.1660	0.25(-0.06, 0.57)	0.1120	0.1497	0.25(-0.07, 0.57)	0.1200	0.1600
precentral	ACME	-0.03(-0.11, 0.05)	0.5100	0.7277	-0.06`(-0.14, 0.01)	0.0920	0.1269	-0.09*(-0.19, -0.02)	0.0100	0.0190	-0.12**(-0.23, -0.03)	0.0060	0.0100	-0.13**(-0.26, -0.04)	0.0000	0.0000	-0.13**(-0.26, -0.03)	0.0040	0.0064
	ADE	0.01(-0.25, 0.28)	0.9380	0.9880	0.12(-0.13, 0.36)	0.3200	0.4436	0.24`(-0.01, 0.48)	0.0640	0.1076	0.35*(0.05, 0.68)	0.0240	0.0384	0.37*(0.05, 0.67)	0.0280	0.0424	0.36*(0.06, 0.68)	0.0160	0.0348
	TE	-0.02(-0.28, 0.26)	0.9020	0.9980	0.07(-0.19, 0.3)	0.6160	0.6503	0.14(-0.1, 0.4)	0.2780	0.2840	0.24(-0.07, 0.57)	0.1360	0.1660	0.24(-0.07, 0.56)	0.1300	0.1497	0.24(-0.07, 0.56)	0.1520	0.1600
precuneus	ACME	-0.02(-0.1, 0.06)	0.5300	0.7277	-0.06`(-0.16, 0)	0.0580	0.1078	-0.1**(-0.2, -0.03)	0.0040	0.0094	-0.14**(-0.27, -0.04)	0.0020	0.0042	-0.14**(-0.28, -0.04)	0.0020	0.0042	-0.15**(-0.28, -0.05)	0.0000	0.0000
	ADE	0.01(-0.26, 0.28)	0.9320	0.9880	0.13(-0.11, 0.36)	0.2920	0.4436	0.26`(0.01, 0.51)	0.0500	0.1018	0.37*(0.06, 0.68)	0.0200	0.0383	0.39*(0.07, 0.71)	0.0200	0.0348	0.41*(0.1, 0.72)	0.0120	0.0343
	TE	-0.01(-0.29, 0.27)	0.9380	0.9980	0.07(-0.17, 0.31)	0.5780	0.6503	0.16(-0.09, 0.43)	0.2280	0.2840	0.23(-0.08, 0.54)	0.1380	0.1660	0.25(-0.08, 0.56)	0.1280	0.1497	0.26(-0.04, 0.56)	0.1120	0.1600
rostral anterior cingulate	ACME	-0.03(-0.09, 0.01)	0.2500	0.6945	-0.05`(-0.13, 0.01)	0.0820	0.1171	-0.08`(-0.17, 0.01)	0.0820	0.0960	-0.11`(-0.23, 0)	0.0500	0.0606	-0.11`(-0.24, 0.01)	0.0680	0.0756	-0.11`(-0.25, 0.01)	0.0740	0.0822
	ADE	0(-0.27, 0.27)	0.9500	0.9880	0.11(-0.12, 0.34)	0.3460	0.4436	0.23`(-0.03, 0.48)	0.0920	0.1150	0.34*(0.03, 0.67)	0.0380	0.0447	0.35*(0.03, 0.67)	0.0360	0.0424	0.35*(0.03, 0.66)	0.0400	0.0457
	TE	-0.02(-0.28, 0.26)	0.8800	0.9980	0.06(-0.18, 0.31)	0.6040	0.6503	0.15(-0.11, 0.41)	0.2600	0.2840	0.23(-0.06, 0.54)	0.1320	0.1660	0.24(-0.07, 0.57)	0.1360	0.1497	0.24(-0.06, 0.55)	0.1300	0.1600
rostral middle frontal	ACME	-0.03(-0.1, 0.03)	0.2820	0.6945	-0.07*(-0.16, -0.01)	0.0300	0.1022	-0.1**(-0.2, -0.02)	0.0000	0.0000	-0.13**(-0.28, -0.04)	0.0020	0.0042	-0.13**(-0.26, -0.03)	0.0040	0.0070	-0.14**(-0.27, -0.04)	0.0040	0.0064
	ADE	0.02(-0.25, 0.28)	0.8780	0.9880	0.14(-0.09, 0.38)	0.2600	0.4436	0.25*(0.01, 0.49)	0.0380	0.0941	0.37*(0.07, 0.67)	0.0100	0.0383	0.38*(0.07, 0.7)	0.0120	0.0282	0.39*(0.09, 0.71)	0.0160	0.0348
	TE	-0.01(-0.28, 0.26)	0.9720	0.9980	0.07(-0.17, 0.31)	0.5740	0.6503	0.15(-0.1, 0.4)	0.2160	0.2840	0.24(-0.06, 0.52)	0.1340	0.1660	0.25(-0.05, 0.56)	0.1100	0.1497	0.25(-0.06, 0.55)	0.1200	0.1600
superior frontal	ACME	-0.03(-0.11, 0.03)	0.3060	0.6945	-0.07*(-0.16, -0.01)	0.0260	0.1022	-0.1**(-0.21, -0.03)	0.0020	0.0053	-0.14*(-0.27, -0.03)	0.0120	0.0185	-0.14**(-0.28, -0.04)	0.0040	0.0070	-0.14**(-0.29, -0.04)	0.0020	0.0044
	ADE	0.03(-0.24, 0.27)	0.8160	0.9880	0.14(-0.1, 0.37)	0.2700	0.4436	0.26`(0, 0.52)	0.0560	0.1018	0.37*(0.05, 0.66)	0.0240	0.0384	0.38*(0.08, 0.69)	0.0180	0.0348	0.39*(0.07, 0.71)	0.0200	0.0348
	TE	-0.01(-0.27, 0.25)	0.9700	0.9980	0.07(-0.17, 0.31)	0.5620	0.6503	0.16(-0.11, 0.42)	0.2600	0.2840	0.23(-0.07, 0.51)	0.1360	0.1660	0.24(-0.07, 0.53)	0.1300	0.1497	0.25(-0.07, 0.55)	0.1400	0.1600
superior parietal	ACME	-0.01(-0.06, 0.05)	0.8380	0.8480	-0.03(-0.09, 0.02)	0.2580	0.3035	-0.05*(-0.12, 0)	0.0280	0.0431	-0.07*(-0.18, -0.01)	0.0160	0.0237	-0.07*(-0.18, -0.01)	0.0200	0.0286	-0.08*(-0.18, -0.01)	0.0200	0.0286
	ADE	-0.01(-0.29, 0.25)	0.9880	0.9880	0.1(-0.14, 0.34)	0.4160	0.4754	0.21(-0.04, 0.45)	0.1040	0.1189	0.31*(0.02, 0.61)	0.0360	0.0447	0.32`(0, 0.63)	0.0520	0.0562	0.33*(0.03, 0.63)	0.0340	0.0436
	TE	-0.01(-0.3, 0.25)	0.9700	0.9980	0.07(-0.17, 0.32)	0.5580	0.6503	0.16(-0.1, 0.41)	0.2200	0.2840	0.24(-0.06, 0.53)	0.1320	0.1660	0.24(-0.06, 0.58)	0.1300	0.1497	0.25(-0.07, 0.55)	0.1120	0.1600
superior temporal	ACME	-0.03(-0.12, 0.05)	0.3580	0.6945	-0.07*(-0.16, 0)	0.0420	0.1022	-0.11**(-0.22, -0.03)	0.0020	0.0053	-0.14**(-0.27, -0.05)	0.0000	0.0000	-0.15**(-0.27, -0.05)	0.0000	0.0000	-0.15**(-0.28, -0.05)	0.0000	0.0000
	ADE	0.03(-0.24, 0.27)	0.8260	0.9880	0.15(-0.08, 0.39)	0.2200	0.4436	0.27*(0.02, 0.52)	0.0340	0.0941	0.38**(0.08, 0.68)	0.0040	0.0383	0.39*(0.08, 0.7)	0.0160	0.0337	0.4**(0.09, 0.7)	0.0060	0.0267
	TE	-0.01(-0.29, 0.27)	0.9620	0.9980	0.08(-0.16, 0.33)	0.5240	0.6503	0.16(-0.09, 0.4)	0.1940	0.2840	0.23(-0.06, 0.55)	0.1300	0.1660	0.24(-0.06, 0.54)	0.1180	0.1497	0.25(-0.06, 0.56)	0.1100	0.1600
supramarginal	ACME	-0.02(-0.11, 0.05)	0.5360	0.7277	-0.06`(-0.15, 0.01)	0.0820	0.1171	-0.1**(-0.2, -0.02)	0.0080	0.0160	-0.14**(-0.26, -0.05)	0.0000	0.0000	-0.14**(-0.27, -0.04)	0.0000	0.0000	-0.14**(-0.27, -0.05)	0.0040	0.0064
	ADE	0.02(-0.27, 0.29)	0.9160	0.9880	0.13(-0.11, 0.38)	0.3160	0.4436	0.26*(0.01, 0.51)	0.0360	0.0941	0.38*(0.07, 0.68)	0.0220	0.0383	0.38*(0.06, 0.71)	0.0160	0.0337	0.39*(0.08, 0.71)	0.0200	0.0348
	TE	-0.01(-0.3, 0.28)	0.9500	0.9980	0.07(-0.16, 0.32)	0.5800	0.6503	0.16(-0.1, 0.42)	0.2120	0.2840	0.24(-0.07, 0.54)	0.1380	0.1660	0.24(-0.09, 0.56)	0.1460	0.1497	0.25(-0.06, 0.56)	0.1080	0.1600
temporal pole	ACME	-0.04(-0.12, 0.03)	0.2600	0.6945	-0.08*(-0.17, -0.01)	0.0200	0.1022	-0.12**(-0.23, -0.04)	0.0000	0.0000	-0.16**(-0.3, -0.06)	0.0020	0.0042	-0.17**(-0.31, -0.06)	0.0020	0.0042	-0.17**(-0.32, -0.06)	0.0020	0.0044
	ADE	0.03(-0.25, 0.3)	0.8060	0.9880	0.16(-0.11, 0.4)	0.2080	0.4436	0.28*(0.02, 0.53)	0.0380	0.0941	0.4*(0.1, 0.69)	0.0100	0.0383	0.41*(0.1, 0.7)	0.0120	0.0282	0.43**(0.11, 0.74)	0.0060	0.0267
	TE	0(-0.29, 0.27)	0.9980	0.9980	0.08(-0.18, 0.32)	0.5280	0.6503	0.16(-0.1, 0.4)	0.2060	0.2840	0.24(-0.08, 0.51)	0.1400	0.1660	0.24(-0.06, 0.52)	0.1220	0.1497	0.25(-0.07, 0.58)	0.1120	0.1600
transverse temporal	ACME	-0.01(-0.09, 0.08)	0.8480	0.8480	-0.03(-0.11, 0.04)	0.4080	0.4316	-0.06(-0.15, 0.01)	0.1240	0.1378	-0.09`(-0.2, 0)	0.0500	0.0606	-0.09*(-0.21, -0.01)	0.0320	0.0427	-0.1*(-0.21, -0.01)	0.0400	0.0516
	ADE	-0.01(-0.27, 0.26)	0.9600	0.9880	0.11(-0.1, 0.32)	0.3660	0.4436	0.21(-0.05, 0.45)	0.1120	0.1244	0.33*(0.04, 0.63)	0.0280	0.0386	0.32*(0.02, 0.61)	0.0360	0.0424	0.34*(0.03, 0.62)	0.0300	0.0414
	TE	-0.01(-0.28, 0.28)	0.9200	0.9980	0.07(-0.14, 0.3)	0.5320	0.6503	0.15(-0.12, 0.4)	0.2720	0.2840	0.23(-0.07, 0.55)	0.1320	0.1660	0.23(-0.07, 0.53)	0.1640	0.1640	0.24(-0.06, 0.53)	0.1160	0.1600
hippocampus	ACME	-0.04(-0.11, 0.02)	0.2020	0.6945	-0.06*(-0.14, 0)	0.0400	0.1022	-0.09*(-0.2, -0.02)	0.0120	0.0200	-0.12**(-0.24, -0.03)	0.0040	0.0070	-0.12**(-0.25, -0.03)	0.0020	0.0042	-0.12**(-0.24, -0.03)	0.0040	0.0064
	ADE	0.01(-0.25, 0.28)	0.9320	0.9880	0.12(-0.11, 0.35)	0.2900	0.4436	0.23`(-0.02, 0.47)	0.0700	0.1076	0.34*(0.06, 0.63)	0.0200	0.0383	0.35*(0.06, 0.67)	0.0200	0.0348	0.37*(0.05, 0.66)	0.0140	0.0348
	TE	-0.03(-0.3, 0.24)	0.8120	0.9980	0.06(-0.17, 0.29)	0.6280	0.6503	0.14(-0.1, 0.38)	0.2480	0.2840	0.22(-0.05, 0.5)	0.1240	0.1660	0.23(-0.06, 0.53)	0.1160	0.1497	0.25(-0.07, 0.54)	0.1080	0.1600
pallidum	ACME	-0.03(-0.09, 0.01)	0.1660	0.6945	-0.05`(-0.12, 0)	0.0580	0.1078	-0.08`(-0.17, 0.01)	0.0660	0.0825	-0.1*(-0.22, 0)	0.0460	0.0594	-0.1*(-0.22, 0)	0.0400	0.0516	-0.1`(-0.23, 0)	0.0600	0.0750
	ADE	0.01(-0.25, 0.26)	0.9720	0.9880	0.11(-0.11, 0.34)	0.3260	0.4436	0.21`(-0.02, 0.46)	0.0880	0.1150	0.31*(0.02, 0.62)	0.0340	0.0447	0.33*(0.03, 0.64)	0.0340	0.0424	0.34*(0.05, 0.67)	0.0260	0.0385
	TE	-0.02(-0.3, 0.24)	0.8640	0.9980	0.06(-0.16, 0.28)	0.5780	0.6503	0.14(-0.08, 0.38)	0.2760	0.2840	0.21(-0.08, 0.5)	0.1600	0.1660	0.23(-0.07, 0.53)	0.1380	0.1497	0.24`(-0.03, 0.52)	0.0900	0.1600
putamen	ACME	-0.08`(-0.19, 0)	0.0520	0.6945		0.0000	0.0000	-0.15**(-0.26, -0.06)	0.0000	0.0000	-0.18**(-0.32, -0.07)	0.0000	0.0000	-0.18**(-0.33, -0.07)	0.0040	0.0070	-0.19**(-0.33, -0.07)	0.0000	0.0000
	ADE	0.06(-0.18, 0.32)	0.6440	0.9880	0.18(-0.05, 0.41)	0.1400	0.4436	0.3**(0.05, 0.55)	0.0080	0.0941	0.41**(0.14, 0.72)	0.0060	0.0383	0.42**(0.13, 0.72)	0.0060	0.0282	0.44**(0.15, 0.73)	0.0060	0.0267
	TE	-0.02(-0.26, 0.26)	0.8660	0.9980	0.06(-0.17, 0.31)	0.5600	0.6503	0.15(-0.08, 0.4)	0.2180	0.2840	0.23(-0.05, 0.54)	0.1160	0.1660	0.24(-0.05, 0.54)	0.1040	0.1497	0.25`(-0.05, 0.57)	0.0940	0.1600
thalamus proper	ACME	-0.04(-0.1, 0.01)	0.1260	0.6945	-0.06`(-0.13, 0)	0.0620	0.1078	-0.08`(-0.18, 0.01)	0.0640	0.0825	-0.1`(-0.21, 0)	0.0540	0.0617	-0.1`(-0.22, 0)	0.0640	0.0731	-0.1`(-0.23, 0.01)	0.0620	0.0752
	ADE	0.02(-0.22, 0.27)	0.8940	0.9880	0.13(-0.13, 0.39)	0.2940	0.4436	0.23`(-0.02, 0.49)	0.0780	0.1076	0.33*(0.05, 0.65)	0.0260	0.0385	0.34*(0.01, 0.67)	0.0460	0.0511	0.35*(0.02, 0.66)	0.0320	0.0427
	TE	-0.01(-0.27, 0.25)	0.9460	0.9980	0.07(-0.19, 0.31)	0.5540	0.6503	0.16(-0.11, 0.4)	0.2160	0.2840	0.24(-0.05, 0.54)	0.1060	0.1660	0.25(-0.07, 0.54)	0.1080	0.1497	0.25(-0.06, 0.55)	0.1000	0.1600

*P-value thresholds based on unadjusted comparison:’0.1; * <0.05; ** <0.005. Regions/Cell with adjusted P-value <0.05 are highlighted in yellow and trend in bold. Annotations: EYO= estimated years to symptom onset; CI= confidence interval. ACME= average causal mediation effect; ADE = average direct effect; TE = total effect.*

Table S11 – Mediation Analyses conditional per EYO with SVD markers as mediator

SVD Variable	Effect	EYO=-15		EYO=-10		EYO=-5		EYO=0		EYO=0.5		EYO=1	
		Estimates (95% CI)	*P* value	Estimates (95% CI)	*P* value	Estimates (95% CI)	*P* value	Estimates (95% CI)	*P* value	Estimates (95% CI)	*P* value	Estimates (95% CI)	*P* value
**PSMD**	ACME	-0.04 (-0.2, 0.11)	0.598	0.03 (-0.12, 0.17)	0.706	0.1 (-0.06, 0.27)	0.198	0.18* (0.01, 0.39)	0.036	0.18* (0.01, 0.38)	0.042	0.19* (0.01, 0.41)	0.044
	ADE	0.14 (-0.24, 0.51)	0.454	0.18 (-0.17, 0.51)	0.332	0.24 (-0.13, 0.56)	0.212	0.28 (-0.11, 0.69)	0.158	0.29 (-0.14, 0.66)	0.164	0.3 (-0.14, 0.7)	0.178
	TE	0.1 (-0.29, 0.48)	0.632	0.2 (-0.15, 0.57)	0.282	**0.34` (-0.04, 0.72)**	**0.086**	0.46* (0.07, 0.89)	0.024	0.47* (0, 0.88)	0.048	0.49* (0.03, 0.93)	0.034
**Total Periventricular WMH**	ACME	-0.01 (-0.08, 0.06)	0.836	0.02 (-0.04, 0.1)	0.476	0.05 (-0.01, 0.15)	0.138	**0.08` (-0.01, 0.22)**	**0.09**	**0.08` (-0.01, 0.22)**	**0.084**	**0.09` (-0.01, 0.24)**	**0.07**
	ADE	-0.05 (-0.36, 0.25)	0.756	0 (-0.28, 0.28)	0.996	0.05 (-0.24, 0.35)	0.712	0.09 (-0.28, 0.45)	0.64	0.1 (-0.27, 0.48)	0.62	0.12 (-0.25, 0.51)	0.54
	TE	-0.06 (-0.37, 0.25)	0.748	0.03 (-0.28, 0.3)	0.858	0.1 (-0.21, 0.4)	0.5	0.17 (-0.2, 0.54)	0.356	0.19 (-0.18, 0.55)	0.328	0.2 (-0.16, 0.57)	0.298
**Total mH count**	ACME	0 (-0.03, 0.02)	0.994	0 (-0.02, 0.02)	0.938	0 (-0.03, 0.02)	0.992	0 (-0.03, 0.03)	0.98	0 (-0.03, 0.02)	0.97	0 (-0.03, 0.02)	0.938
	ADE	0.01 (-0.26, 0.29)	0.932	0.14 (-0.11, 0.39)	0.31	0.27* (0.02, 0.55)	0.028	0.41* (0.1, 0.73)	0.012	0.41* (0.07, 0.72)	0.016	0.44** (0.12, 0.75)	0.008
	TE	0.01 (-0.26, 0.3)	0.938	0.13 (-0.11, 0.39)	0.322	0.27* (0.02, 0.54)	0.028	0.41* (0.1, 0.73)	0.012	0.41* (0.07, 0.72)	0.018	0.43** (0.12, 0.76)	0.008

*P-value thresholds: `<0.1; * <0.05; ** <0.005. Significant effects are highlighted in yellow and trend in in bold. Annotations: SVD= small vessel disease; PSMD= peak-width of skeletonized mean diffusivity; WMH= whitter matter hyperintensity; mH= microhemorrhage; EYO= estimated years to symptom onset; CI= confidence interval. ACME= average causal mediation effect; ADE = average direct effect; TE = total effect.*
